# Virtual Plant Tissue: Building Blocks for Next-Generation Plant Growth Simulation

**DOI:** 10.3389/fpls.2017.00686

**Published:** 2017-05-04

**Authors:** Dirk De Vos, Abdiravuf Dzhurakhalov, Sean Stijven, Przemyslaw Klosiewicz, Gerrit T. S. Beemster, Jan Broeckhove

**Affiliations:** ^1^Integrated Molecular Plant Physiology Research, Department of Biology, University of AntwerpAntwerp, Belgium; ^2^Modeling of Systems and Internet Communication, Department of Mathematics and Computer Science, University of AntwerpAntwerp, Belgium

**Keywords:** modeling, simulation, software, growth, development, coupling, modular

## Abstract

**Motivation:** Computational modeling of plant developmental processes is becoming increasingly important. Cellular resolution plant tissue simulators have been developed, yet they are typically describing physiological processes in an isolated way, strongly delimited in space and time.

**Results:** With plant systems biology moving toward an integrative perspective on development we have built the Virtual Plant Tissue (VPTissue) package to couple functional modules or models in the same framework and across different frameworks. Multiple levels of model integration and coordination enable combining existing and new models from different sources, with diverse options in terms of input/output. Besides the core simulator the toolset also comprises a tissue editor for manipulating tissue geometry and cell, wall, and node attributes in an interactive manner. A parameter exploration tool is available to study parameter dependence of simulation results by distributing calculations over multiple systems.

**Availability:** Virtual Plant Tissue is available as open source (EUPL license) on Bitbucket (https://bitbucket.org/vptissue/vptissue). The project has a website https://vptissue.bitbucket.io.

## Introduction

Driven by experimental advances during the last decade, plant biology has witnessed the emergence of simulation models that complement and enrich our conceptual understanding of elementary processes in plant development (Mjolsness, 2006; De Vos et al., 2012; Hodgman and Ajmera, 2015). Models explaining basic characteristics of phyllotaxis, leaf venation, primary, and lateral root growth, and so forth are becoming more sophisticated but remain predominantly restricted to localized and simplified descriptions in time and space. Various plant spatial modeling frameworks or tools have been reported aimed at modeling isolated systems at the cellular scale and tissue scale such as VV (http://algorithmicbotany.org/papers/smithco.dis2006.pdf), CellModeller (Dupuy et al., 2008), VirtualLeaf (Merks et al., 2011) and CellZilla (Shapiro et al., 2013; Cellerator: https://sourceforge.net/projects/cambium). The chemical and mechanical properties of the flower bud have been modeled in 3D at cellular resolution with SOFA (Boudon et al., 2015). Other software primarily targets the organ to whole plant scale and beyond, such as L-systems (Prusinkiewicz, 2004; Allen et al., 2005), GroIMP (Hemmerling et al., 2008; Kniemeyer, 2008) OpenAlea (Pradal et al., 2008), CrossTalk (Draye and Pagès, 2007) and Organism (http://dev.thep.lu.se/organism).

Plant growth regulators are known to be subjected to long-range, inter-organ transport, and the same molecular signals play an often subtly different role during successive developmental stages (Kalve et al., 2014). A “grand unified model” approach could in principle accommodate such complexity, but presents itself as a task with a prohibitive computational and developmental cost. Future advances in high performance computing will likely improve the outlook on that issue. However, the coupled simulation of existing models, although it presents important issues with interoperability and still requires careful matching and fine-tuning of models is arguably a better strategy. With different models developed by different groups in diverse frameworks in the systems biology landscape, cross-language operability is crucial to avoid duplication of work. Despite the availability of several frameworks, with varying degrees of modularity and potential for coupling individual models, to our knowledge cellular resolution tissue models have not been coupled yet. Here, we present a software package originally based on the VirtualLeaf framework but completely re-engineered for a modular approach that conserves functional units (sub-models or models), enabling reuse of existing (sub-) models and provides sufficient performance and flexibility to allow mutual communication and coordinated time evolution of such models.

## Methods

**Figure 1:**
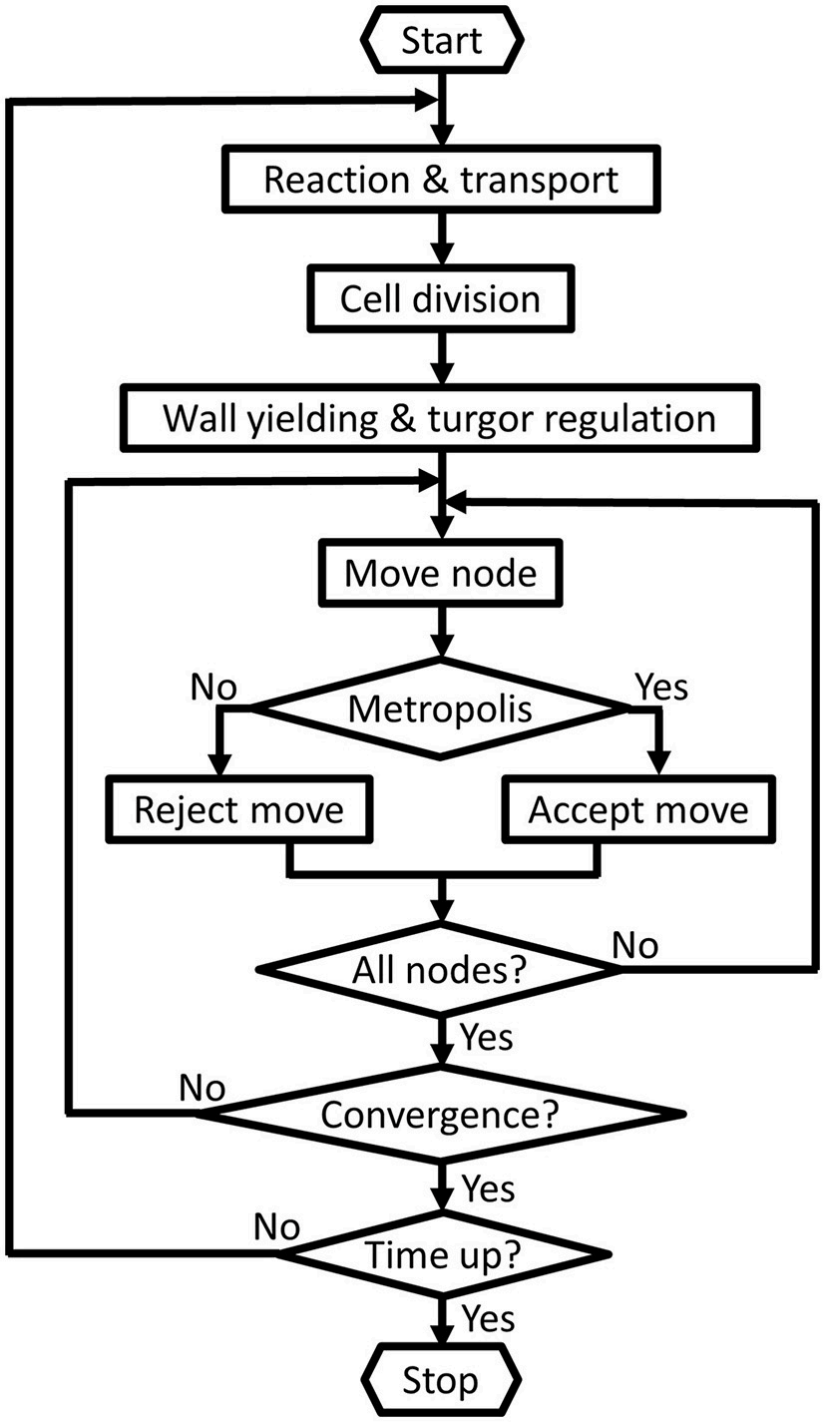
**Basic control flow diagram**. First the slower biological processes such as the reactions, transport, cell division, regulation of turgor pressure and wall yielding are performed. Then iteratively all nodes are attempted to be displaced and [this is called one Monte Carlo (MC) step]. The MC steps are repeated until the system converges to its equilibration state, i.e., a sufficient balance between the turgor pressures and cell walls' resistances. At the end of each MC step the energy change is typically evaluated by: $-\underset{nodes}{\sum }\Delta E_{i}<E_{th},$ where *E*_*th*_ represents the tolerance of the |∑ Δ*E_i_*| convergence. If the system does not satisfy this criterion the complete cycle is repeated until equilibration. Other termination options, such as a sliding window criterion (Dzhurakhalov et al., 2015a), can be specified via the input data file.

**Figure 2:**
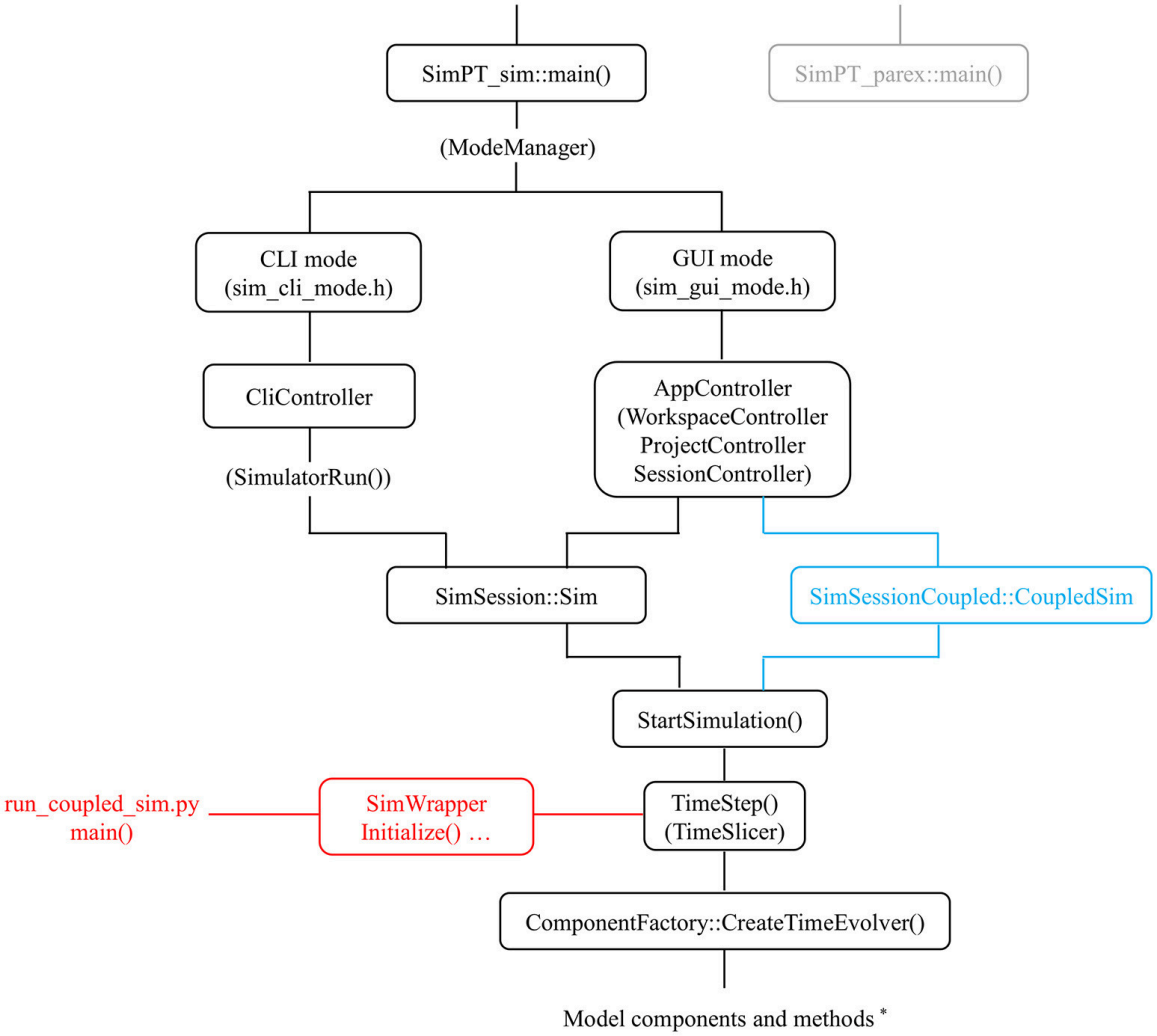
**Modularity in the code**. Some aspects of call hierarchy (main classes/methods) in relation to modularity at 3 levels: via a time_evolver class [used by CreateTimeEvolver()] which organizes model components^*^; via the internal coupler mechanism (CoupledSim; blue color); and via the (here Python) wrapper class (SimWrapper; red color) which interfaces with external models. Two main options are available for the simPT_sim module (standard simulator): command line (CLI) and graphical (GUI) mode. The simPT_parex (gray color) module allows automated parameter exploration via a compute server and uses similar functionality as the CLI mode.

Virtual Plant Tissue can be considered an offspring to VirtualLeaf (Merks et al., 2011), yet represents an entirely new codebase. Concerning language conformance, the forward perspective was taken, aiming to make the code base last as long as possible. Virtual Plant Tissue is written in C++ 11, using familiar design patterns (Gamma et al., 1995) and using the cppcheck tool (cppcheck.sourceforge.net) to analyse the code for design deficiencies. The Model-View-Controller (MVC) design was used to make the simulator more transparent. Adding and changing output features is now more flexible and extensible. Another design choice was to not only mold biological concepts in well-defined classes (Mesh, Cell, Wall, Edge, Node) but also algorithmic entities like CellDivider, NodeInserter or the various time evolution schemes. Figure 1 represents one possible scheme (see Results). Some code elements (classes and functions) crucial in determining different simulation modes/options are presented in Figure 2. Both command line and graphical runs are possible: via the CliController or AppController class, respectively. The latter can operate on a single simulation (Sim) object or on a CoupledSim object for internally coupled simulations. All running modes converge on a central TimeStep() function that organizes the selection and execution of the different model components that define one model or two coupled models. TimeStep() is also available for external C++, Python, and Java programs via a wrapper class.

**Figure 3:**
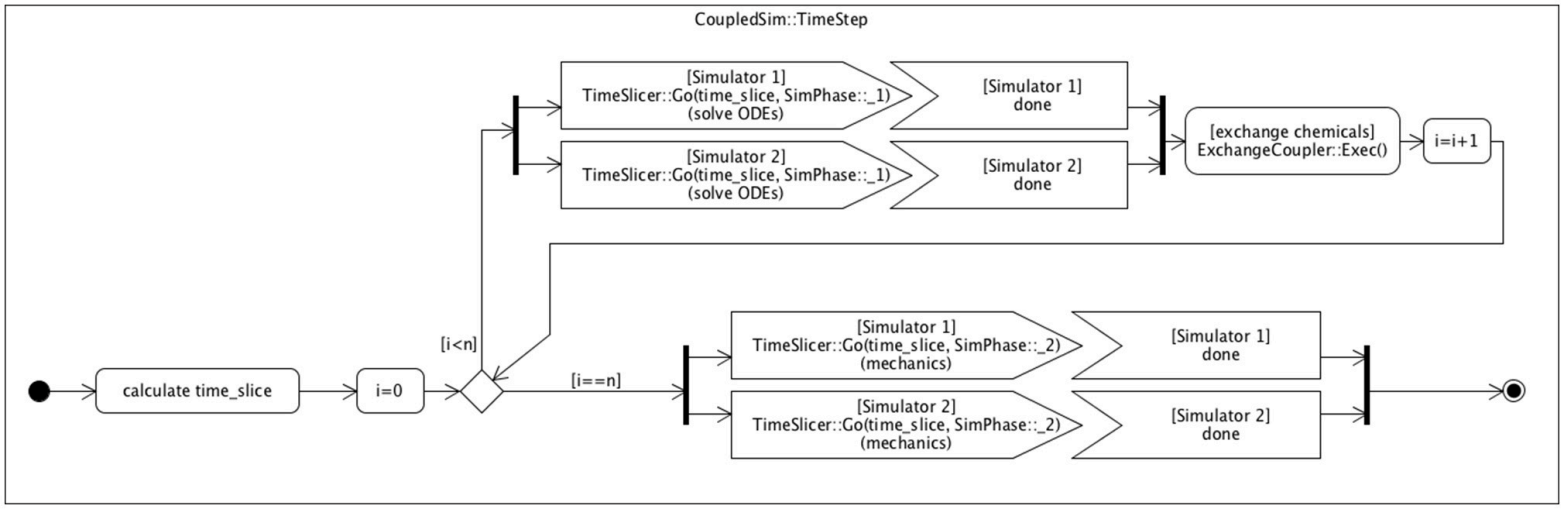
**Scheme with control flow for internal coupling**. Multiple simulation instances can be run in parallel, while interacting in an organized way through a coupler class (Figure 2). Each simulation time step is divided in n time slices (according to the sim_ODE_coupling_steps parameter) in which the chemical levels of the models evolve independently, followed by an exchange event (via the ExchangeCoupler coupler). This exchange ensures that the cells at the (coupling) boundary of the respective models can adapt their boundary conditions to the chemical concentrations of the cells of the other model directly coupled to them. A separate VPTissue data file defines which cells are specifically coupled, through which chemicals, and with which transport or permeability constant. If cell x of model 1 is coupled to cell y of model 2, then the coupler ensures that the correct chemical concentration of cell y becomes the boundary condition (concentration) of cell x, and *vice versa*. Each model has a cell2cell_transport_boundary class that determines the transport kinetics between the coupled models (cells) through the modified boundary conditions. After *n* cycles the simulation step finishes by executing the mechanical processes for each model simulation individually.

In the case of internally coupled simulations (Figure 3) each single simulation time step is subdivided into time slices in which chemical levels evolve independently (similar to the Reaction & transport step in Figure 1). After each coupling time slice interacting boundary cells of the coupled simulations exchange information on their respective chemical concentrations. This step is executed by the Coupler class. After *n* iterations the simulation time step finishes by independent evolution of both simulations in terms of cell mechanics and cell division.

**Figure 4:**
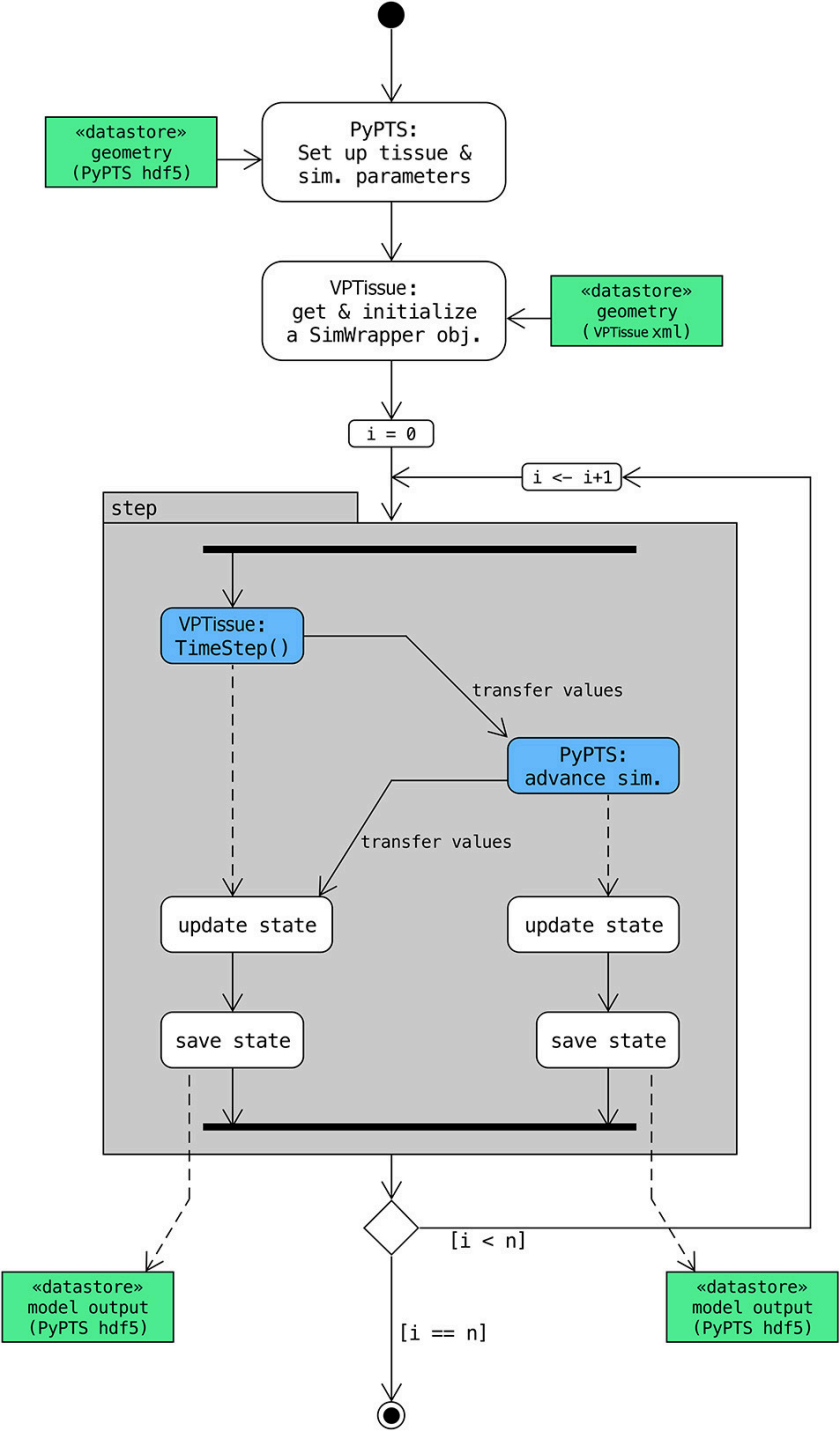
**Scheme with control flow for cross-platform coupling**. In this specific case a (simple root) model defined in Virtual Plant Tissue interacts through its SimWrapper class (Figure 2) with an external (simple leaf) model defined with the PyPTS package. A Python driver/master script (here run_coupled_sim.py) sets up the first model tissue (along with the model parameters) based on an hdf5 input file (leaf.h5) and initializes a SimWrapper object based on an XML input file (simpt_root.xml). Then *n* simulation steps are executed. In each step the master script first orders Virtual Plant Tissue to do a time step for the root model and then provide information on the chemical levels of the coupled cells (those directly interacting in the coupling). That information is used as a boundary condition for the simulation step of the leaf (PyPTS) model which is then executed (numerical integration uses SciPy.integrate). The chemical levels of the leaf model (more precisely of the coupled cells) are then transferred to the Virtual Plant Tissue simulation via the SetMeshState() method of the SimWrapper object. Finally, the I/O functionality of PyPTS is called by the master script to write out the state of both simulations at that time step in separate hdf5 files. Detailed instructions to run this example can be found in the source code (src/main/swig_sim/py_WrapperModel/README.md).

If an external program needs access to the TimeStep() it does so via a C++ SimWrapper class which defines a set of basic interaction functions. In case a Python or Java program wants to call a Virtual Plant Tissue simulation the required glue code needed for interoperability is provided as well (see src/main/swig_sim/swig_interface). In the specific case implemented here (Figure 4) a model defined in Python describing a leaf was coupled with a simple root model. More precisely the PyPTS package (https://pypi.python.org/pypi/PyPTS/0.2.4) is used to set up a leaf tissue in Python based on an hdf5 input file. In the next step the SimWrapper GetXMLState() and Initialize() functions are used to read the root input file. What then follows is an iteration of simulation steps coordinated by a Python script which operates the Python leaf model and the Virtual Plant Tissue root model in succession and forces them to exchange information via the wrapper functions.

The project directory layout is very systematic and follows the Maven convention which is aimed at increasing the build transparency (https://maven.apache.org/maven-conventions.html). Everything used to build project artifacts is placed in directoy/src. Code related files can be found in directory src/main. Documentation files are located in src/doc. Files related to automated unit and scenario testing (e.g., to verify models can be simulated for a defined number of steps) via ctest (see https://cmake.org) and googletest (https://github.com/google/googletest) can be found in src/test. The tests can be run manually by the user by using the make target “test” (for more details on the build see below). Detailed test output is generated by setting the SIMPT_VERBOSE_TESTING flag to “ON” in CMakeBuildConfig.txt. The build generated artifacts are put in a single directory target, separate from the source code, which is completely removed when the project is cleaned.

Git (https://git-scm.com) was used for version control and the Jenkins continuous integration platform (https://jenkins.io) for automatic building and testing (including performance tests). Virtual Plant Tissue builds on Linux/UNIX platforms, Mac OSX platforms and Windows/MinGW platforms. The project is built using the CMake build system (see http://www.cmake.org/), using an out of source build. For those more familiar with the Make build system, in the top level directory, a front-end Makefile is provided to trigger a build. Details on the build procedure and the macros that can be set to configure the build, can be found in the file INSTALL.txt in the top level project directory. The file PLATFORMS.txt in the same directory, lists the platforms (combinations of operating system/compiler/required libraries) on which the project has been successfully built. More information can also be found in the chapter “The Virtual Plant Tissue Software” in the User Manual (see src/doc/latex_user_man/UserManual.pdf). The chapter also highlights more elements of the Virtual Plant Tissue development process related to coding practices, to the project directory layout and to the Continuous Integration cycle of building and testing using the Jenkins tool.

The project maximizes code re-usage through third party software components (libraries and/or header files). We distinguish between non-included or external dependencies and included dependencies. The former are major software, installed on almost all systems such as Qt (https://www.qt.io/) or Boost (www.boost.org). Unlike in VirtualLeaf, Boost's ptree container is used to hold configuration data with a hierarchical structure (such as tissues). This container provides easy access and input/output to a number of file formats. Furthermore, there is no longer a need for a pre-compilation step and model parameters can now be more easily added to the data files. The latter are smaller public domain software that we have included in source format in the project and are built directly into the application by our build procedure. Some of the non-included dependencies are required to build the application (a GCC or Clang C++ compiler, Qt4 or Qt5 and Boost 1.53+), others are optional (e.g., HDF5: https://www.hdfgroup.org/HDF5, SWIG 2.0). Our build procedure, based on the CMake tool, automatically inspects the system and if the optional dependency is not present, the corresponding build steps are simply skipped. The user can also set some macros that consciously skip some build steps, even if the dependency is available (see the file INSTALL.txt in the top level project directory). A complete list of dependencies, both non-included and included, with a (very) brief description of their function is available in the file DEPENDENCIES.txt in the top level directory of the project.

## Results

We have opted to keep the simplifying, yet fast two-dimensional vertex-based representation of plant tissue of its predecessor VirtualLeaf. This approach requires relying on inherent (pseudo-)symmetries of the tissues, and at the same time it allows describing biophysical and biochemical processes in great detail. A diverse set of C++ model classes incorporated in the framework representing various functional units supports that principle (Table 1).

**Table 1:** **Main model components and brief function description**.

**Component**	**Function**
cell_chemistry	Equations for intra-cellular reaction dynamics
cell_color	Rules for output color of cells
cell_daughters	Rules for partitioning of chemicals (and other properties) to daughter cells
cell_housekeep	Equations for updating properties related to cell and wall expansion
cell_split	Rules determining cell division
cell2cell_transport	Equations for transport across cell walls
cell2cel_transport_boundary	Equations for transport across cell walls at the boundary (with external environment or with coupled model)
delta_hamiltonian	Energy function based on energy-difference upon node displacement
hamiltonian	Energy function on per cell-basis (tracks total energy)
move_generator	Generator of random node displacements
time_evolver	Defines time evolution scheme
wall_chemistry	Equations for reaction dynamics in cell walls

*The listed components represent the building blocks of all Virtual Plant Tissue models. Various options are provided in the source code (see user manual, Appendix A). The choice for each component is specified in the VPTissue data file. Several pre-defined models are available. The most noteworthy stand-alone models are: AuxinGrowth^$^ (Merks et al., 2011), Blad0032 (De Vos et al., 2015), Blad0128, Blad0512, Geometric^$^, Meinhardt^$^ (Meinhardt et al., 1998), NoGrowth^$^ (Merks et al., 2011), SmithPhyllotaxis (Smith et al., 2006), TipGrowth^$^, Wortel (De Vos et al., 2014). ^$^Indicates models originating from and cross validated with VirtualLeaf*.

### Basic geometry and simulation logic

Fundamentally, a plant tissue is described in Virtual Plant Tissue as a mesh consisting of polygonal cells that consist of nodes (vertices) connected by edges. The cells have attributes (geometric, chemical, and others) and neighboring cells share an apoplast segment or “wall” (consisting of one or more edges). The walls in fact represent two physiologically distinct cell wall (and plasma membrane) domains on either side of the middle lamella and therefore have neighbor cell specific attributes as well as others (Supplementary Figure 1). Thanks to the “transporter” array attribute, PIN-FORMED (PIN; Krecek et al., 2009) transporter levels can be assigned to the cell wall segments and therefore, like VirtualLeaf (Merks et al., 2007; van Mourik et al., 2012), Virtual Plant Tissue is suitable to study auxin transport and PIN polarization dynamics (the “AuxinGrowth” model is derived from Merks et al., 2007). However, unlike other modeling approaches such as cellular Potts models (Grieneisen et al., 2007) or vertex-based modeling with cell partitioning (Abley et al., 2013) intracellular hormone concentration gradients cannot currently be represented. On the other hand the latter types of models are typically associated with a high computational cost and have other issues (for instance cell sliding in lattice based models). Virtual Plant Tissue can efficiently simulate large tissue structures (Supplementary Table 1, Supplementary Figure 2), however, some implicit assumptions have to be considered. An implicit assumption is that intracellular gradients are small, which for auxin may not be the case for cells as long as 100 μm (Kramer, 2008). Another inherent assumption is that lateral diffusion in the apoplast is negligible which in principle needs to be assessed case by case.

The state of the tissue at a specific time is described in a single XML input file or “leaf file” (in accordance with Merks et al., 2011; the leaf files are, however, not cross-compatible). An input file contains model specific parameters (including names for the components or modules that define the model), but also lists all cells, nodes and walls together with their unique attributes (cf. user manual: Supplementary File 1). Fundamentally, the dynamic processes during a simulation time step are separated into a phase with slow biochemical and biophysical processes governed by ordinary differential equations and algebraic rules, and a phase with instantaneous (elastic) wall mechanical processes, governed by a Monte Carlo sampling strategy to approximate the equilibrium state (Figure 1). This type of time-scale “separation” and the use of a generalized energy function or Hamiltonian is an efficient and established modeling approach (Wabnik et al., 2010; De Rybel et al., 2014). Since this only excludes simulating very fast (elastic) mechanical changes (taking <1 min: see for instance Proseus et al., 1999), it does not strongly limit the scope of plant biological questions to be studied. The ordering and composition of the phases is customizable as explained below.

### Modularity at multiple levels

The Virtual Plant Tissue code structure has been designed for a modular model building and simulation approach at three principal levels: (i) combining functional units within a model, (ii) coupling models within Virtual Plant Tissue, and (iii) coupling with external models of other frameworks (Figure 1). In Virtual Plant Tissue biological as well as algorithmic concepts/modules have been molded into classes (Table 1, Supplementary File 1). Importantly, the regulation of the time evolution scheme, defining type and sequence of computational processes (cell division, wall yielding, hormone transport, …), is encapsulated in a separate time evolver class. Generally, a great flexibility to define model simulations is available to the user by editing parameters listed in the input XML file. Importantly, all parameters (including model/module selection and time evolution algorithm) can be changed dynamically, with every change taking effect at the start of the time step following that change. This can be done either interactively with the “gui” or as the result of an automated computation, i.e., when certain conditions are met such as surpassing a threshold value of number of cells or simulated time. One could for instance define a component of components, for example a cell division (cell_split) module which selects either symmetric or asymmetric division (both defined in separate modules) based on cell type (pavement cell or meristemoid cell).

**Figure 5:**
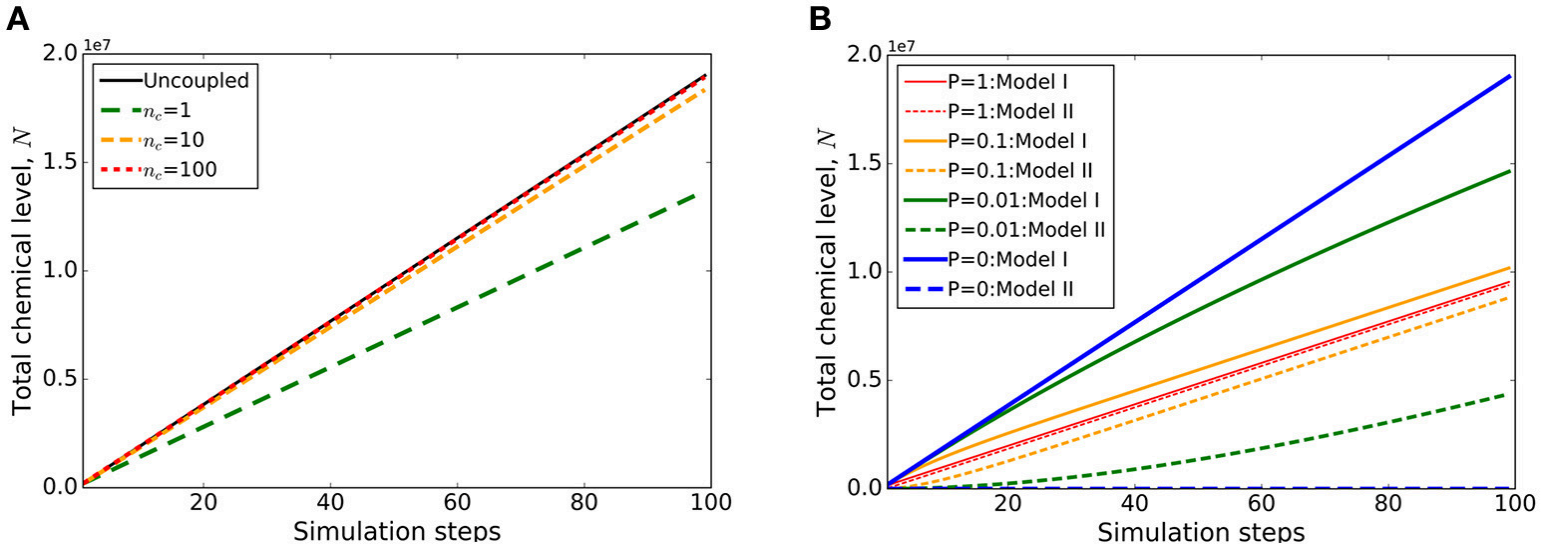
**Example output of internal coupling with Virtual Plant Tissue**. Coupled simulation output of two structurally identical tissues (from models TestCoupling_I and TestCoupling_II, see source code for details). The cells of the first tissue produce a chemical (at a constant rate) that is transported to the second tissue (first order passive transport with a permeability constant *P* = 1 (length unit)/(time unit)). **(A)** The total chemical build-up in the two coupled tissue models as a function of the number of simulation steps. The effect of varying the coupling frequency (*n*_*c*_: number of coupling steps per time step) on chemical levels summed over the two models is demonstrated. Only at a sufficiently high number of coupling steps per time step (*n*_*c*_ = 100) the total chemical levels converge to that of the uncoupled situation (“Uncoupled”). This demonstrates that for this type of coupling (ExchangeCoupler) the coupling frequency has to be set sufficiently high to ensure mass conservation. **(B)** Evolution of the chemical build-up in the respective models (labeled I and II) for different permeabilities [*P* = 1, 0.1, 0.01, 0 (length unit)/(time unit); *n*_*c*_ in all cases = 100], with higher rates leading to more equal distribution of the chemical.

The core simulator has been isolated into a single class which enables multiple instances of simulations to be run in parallel via an internal interface. This represents a higher level of modularity linking internal models/simulations. A Coupler type class defines how multiple simulations can communicate. An example coupler class is available that maximally preserves the respective models' individual character by restriction of information exchange through modified boundary conditions (ExchangeCoupler class (Figure 2). With a dedicated parameter in the VPTissue input file the coupling frequency can be optimized (Figure 5). This is a necessary feature since the coupling step (time slice) needs to be small enough to ensure mass conservation across the boundary. In the example of Figure 5A one of two basic coupled models only produces a chemical (model details in Supplementary Text 1.1). The accumulation of that chemical in the uncoupled case is only closely approximated if the coupling constant is high enough (leading to intensive updating of boundary condition). As expected increasing the permeability constant (“diffusion” in the xml file) for chemical exchange between models leads to nearly equal partitioning of the chemical (Figure 5B).

**Figure 6:**
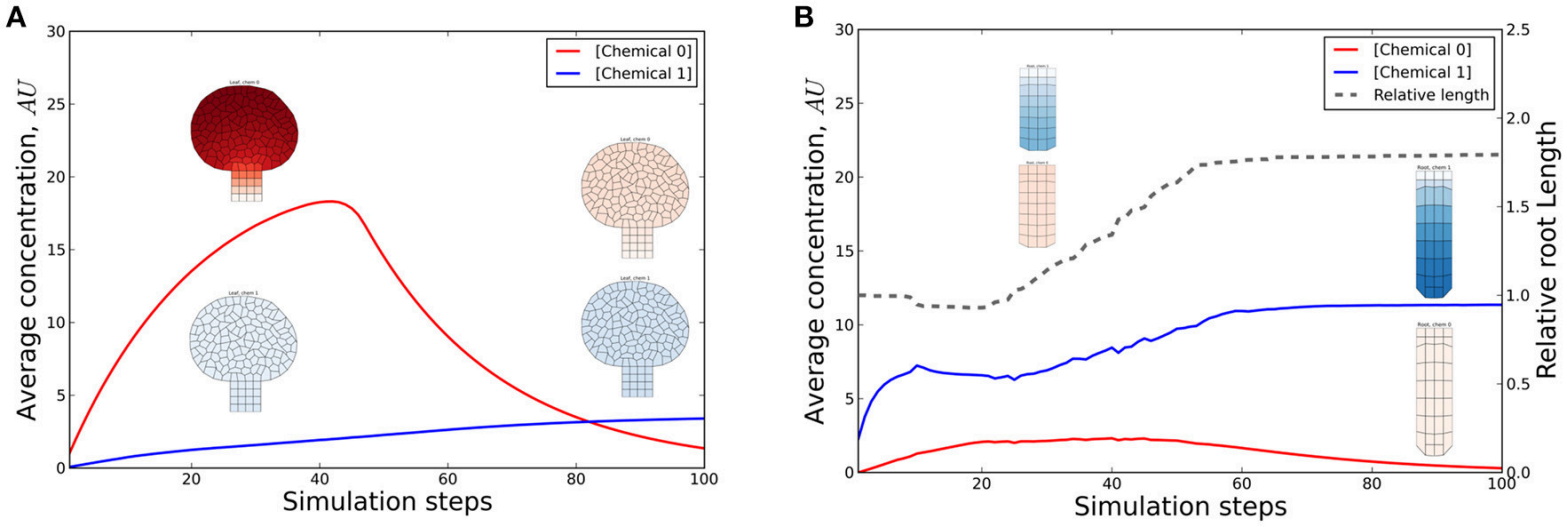
**Chemical and length dynamics in a cross-platform simulation**. In this coupled simulation a long range signal produced in the leaf is transported to the root where it accumulates and induces growth. Through reverse transport of a second signal produced in the root, the first signal is feedback inhibited which results in growth arrest. The first model (leaf) describes a static tissue with all cells producing a chemical (“0”) at a constant rate and degrading it proportional to the concentration (first order decay). Chemical 0 (depicted by red graph and red tissue coloring) is transported (described by an equation describing first order passive transport) to the tissue from the second (root) model. The root does not produce chemical 0, but accumulates it through the coupled transport. If a concentration threshold is reached the root starts growing (dashed line). Simultaneously chemical 1 (depicted by blue graph and blue tissue coloring) is produced (and degraded) by the root and is transported to the leaf (which does not produce that chemical). If a concentration threshold of chemical 1 is subsequently exceeded in the leaf, the local production of chemical 0 is stopped. This eventually leads to growth arrest of the root through the same mechanism as before. Multiple feedback mechanisms are therefore present. **(A,B)** Evolution of chemical concentrations in the leaf and root model, respectively, with snapshots of the tissues (at 30 and 90 simulation steps). The full time evolution of the tissues is shown in Supplementary Video 1. More details can be found in the source code and user manual.

A SWIG adapter has been implemented to define a clean external interface opening up the possibility for coupling multiple models implemented on different platforms. The interface has methods that are also defined in a wrapper class that allows the Virtual Plant Tissue core simulator to be accessed from an external program that can be written in a different language (Python or Java) via native wrapper objects (Figure 4). As an example a model defined in Python (with the PyPTS package,) describing a leaf was coupled with a simple root model defined in Virtual Plant Tissue (Figure 6, Supplementary Video 1, model details in Supplementary Text 1.2). In this particular case the Python model is supplemented with instructions to coordinate the coupling and simulation events (a separate driver or organizer script would work equally well). The (geometrically static) Python leaf model accumulates chemical “0,” which is transported to the Virtual Plant Tissue root model. The root responds above a threshold by growing and increasing chemical “1” production, which will be transported to the leaf and eventually will shut down chemical “0” production and subsequently root growth.

**Figure 7:**
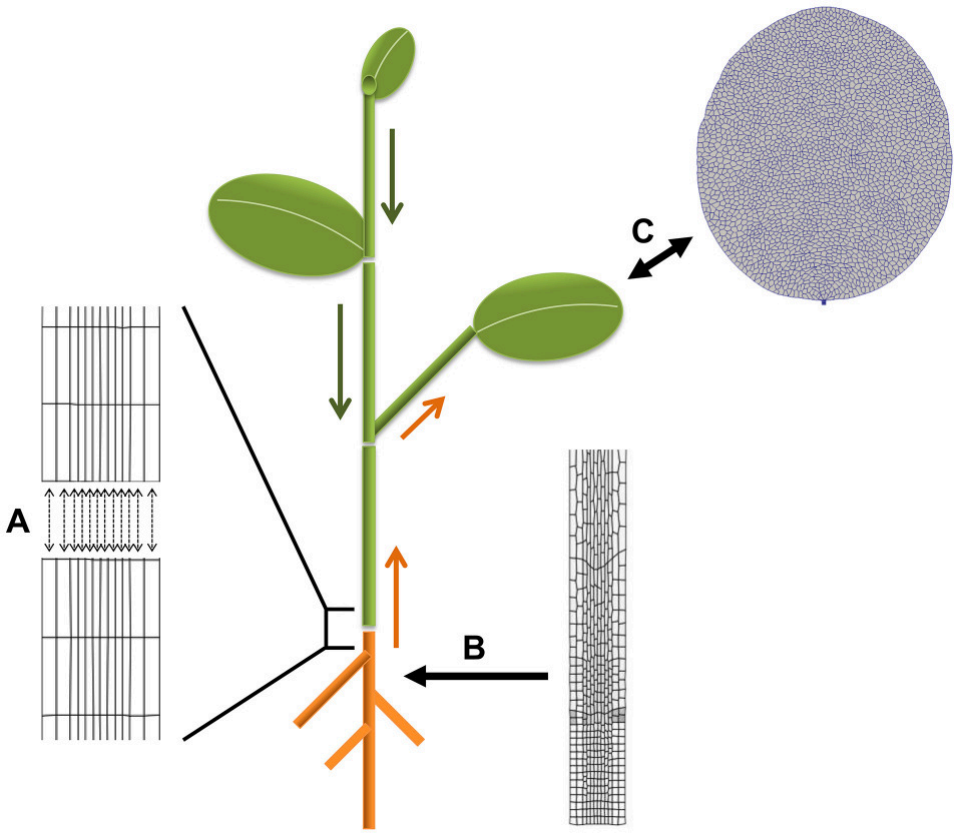
**Biological applications for coupled simulations**. Plants can be considered as modular organisms developing as repetitions of constructional units (Bell, 1991). This is utilized in functional-structural modeling frameworks to simulate plant growth dynamics. The segment or growth units typically have their own descriptive (non-mechanistic) growth equations. Combining them can produce a realistic picture of whole plants which can be set to grow in specific environmental conditions. **(A–C)** Illustrate different options to benefit from this principle for coupled simulations with Virtual Plant Tissue. **(A)** VPTissue's internal interface enables pairwise chemical exchange as represented by the arrows. For instance a root-stem coupling could involve exchange of nutrients and hormones such as auxin and cytokinin. **(B)** The external wrapper interface could regularly feed an architectural model of the root with information on the size of the primary root from a cellular root model growing in VPTissue. **(C)** A VPTissue model of the growing leaf could have bidirectional exchange through the external (wrapper) interface with an architectural model of the shoot simulated in an FSPM framework. Depending on the developmental stage of the shoot and the leaf transport of sugars, auxin or other signals could preferentially be directed in or out of the VPTissue leaf. From the standpoint of the vertex-based leaf model, the FSPM shoot model effectively becomes a dynamic boundary condition. Shoot apical meristem models could also benefit from such an approach.

Concrete biological questions through the described approaches for coupled simulations are diverse. For instance: how does inter-organ transport of assimilates and hormones affect leaf growth (e.g., Bhalerao et al., 2002; Ljung et al., 2015; Notaguchi and Okamoto, 2015)? This could be tackled by coupling an architectural plant model (functional-structural plant model; Vos et al., 2010) of the shoot with a vertex-based model of leaf growth in our framework (a starting leaf model is already available). The most likely candidates for exchange through the external (wrapper) interface are sugars and hormones with the fluxes depending on the developmental state of the respective organs. A direct internal coupling of VPTissue based root and shoot models is feasible too (with minerals and hormones as candidates for exchange). Another possibility for internal coupling is to study the influence of plant architecture (branching) on polar auxin transport (Bennett et al., 2016). Stem segments could be coupled in a cell-based way to exchange auxin. Some of these ideas are illustrated in Figure 7.

### Programming with virtual plant tissue

Despite the extensive efforts to make the framework readable and minimizing dependencies in the code, developers adopting the platform will still need to spend some time familiarizing with it. Basic tasks to master are adding models, model components and model attributes to the code base. Adding a model requires defining an input file as well as some preferences files (in src/main/models/'model family'/resources). One can copy an existing model as the template, use an xml editor or use the Virtual Plant Tissue Editor (see below) for that. New model component files need to be added to src/main/models/'model family'/components/'component type'. Each new component also needs to be added to the local factories.h and factories.cpp files and to the CMakeLists.txt file one level higher. A convenient feature made possible through the Virtual Plant Tissue code modularity is the possibility to define a (meta-)component that uses other model components from the same component type. For instance, one could define a leaf growth model that contains different cell types such as pavement or meristemoid cells. A cell division meta-component could then simply refer to separate dedicated pavement and meristemoid division modules without the need to combine them and duplicate a lot of code. The steps for adding an attribute depend on the type (belonging to simulation, cell, wall, tissue, or node). Detailed help on programming with Virtual Plant Tissue can be found in the user manual (Chapter 7).

### Models, components, and algorithmic choices

Diverse models are provided in the current Virtual Plant Tissue distribution (some originally from VirtualLeaf). Several of them are meant for demonstration or as a starting point for building more advanced models, for instance to study phyllotactic patterning (model based on Smith et al., 2006) and leaf venation (with Meinhardt and AuxinGrowth models). Other models correspond exactly to what was published, including root and leaf models. They can also be used to study regulatory networks involved in leaf and root growth. Some model simulation snapshots can be seen in Supplementary Figure 2. The main components defining a model are listed in Table 1 and can be divided into biological and Monte Carlo mechanics modules. Thanks to the modularity of the Virtual Plant Tissue code a new component type can be easily added and used by different models or model versions. For the biological component types cell_chemistry, wall_chemistry and cell2cell_transport the time organization within each simulation time step is determined by a differential equation solver. On the other hand, components like cell_housekeep, cell_split and cell_daughters contain rules that are evaluated only once per simulation step. For cell division (cell_split) the options currently available are still the same as in VirtualLeaf: cells divide into equal daughter cell areas based on a pre-specified orientation of the division axis or a division axis perpendicular to the cell's axis of inertia (Merks et al., 2011). For the mechanical components different options are now available. The original Hamiltonian of VirtualLeaf is now called “PlainGC” and uses the difference of absolute target cell areas and actual cell areas (Merks et al., 2011). In the “ModifiedGC” the relative differences of the cell areas are used which avoids terms of larger cells to dominate the Hamiltonian (Supplementary Figure 3).

$$\begin{array}{l} \text{Original}:H=\lambda_{A}\underset{i}{\sum }{\left(a\left(i\right)-A_{T}\left(i\right)\right)}^{2}+\lambda_{M}\underset{j}{\sum }{\left(l\left(j\right)-L_{T}\left(j\right)\right)}^{2},\\ \text{Modified}:H=\lambda_{A}\underset{i}{\sum }{\left(\frac{a\left(i\right)-A_{T}\left(i\right)}{a\left(i\right)}\right)}^{2}+\lambda_{M}\underset{j}{\sum }{\left(l\left(j\right)-L_{T}\left(j\right)\right)}^{2}, \end{array}$$

where indices *i* and *j* sum over all cells and polygon edges, respectively, λ_*A*_ is a parameter setting the cells' resistance to compression or expansion, and λ_*M*_ is a spring constant. *A*_*T*_ is the cell's target area, *L*_*T*_ the wall element target length. The target length is constant and the same for all wall elements (edges) and new nodes can be inserted as soon as an edge reaches a critical length (“target_node_distance”). In the Hamiltonian of the “ElasticWall” model the edge length constraint is replaced by an elastic energy term and each wall has its individual wall strength and its variable rest length (specified in the xml data file). This provides more flexibility to describe wall mechanics. The “Maxwell” Hamiltonian additionally replaces the target area constraint by a turgor pressure term in analogy with Corson et al. (2009):

$$\begin{array}{l} \text{Maxwell}:H=-\underset{cells}{\sum }PS\left(i\right)+\frac{Eh}{2}\underset{walls}{\sum }l_{0}\left(j\right){\left(\frac{l\left(j\right)}{l_{0}\left(j\right)}-1\right)}^{2}. \end{array}$$

With the elastic modulus, *h* the uniform wall thickness, *l(j)* and *l*_0_(j) the actual and rest lengths of wall *j*, respectively, the turgor pressure, and *S(i)* the area of cell *i*.

Every time step each cell's solute quantity and each wall's rest length are updated through the corresponding cell_housekeep module. For the ElasticWall model irreversible wall extension happens by increasing wall rest length by a constant fractional amount if the actual wall length exceeds a threshold (for instance if 50% higher than the rest length). In the Maxwell model the wall rest length and solute concentration are updated according to the solution of differential equations (Equations 2, 8) described in Corson et al. (2009) as applied to one Virtual Plant Tissue simulation time step. The repertoire of mechanical modules is useful to investigate the influence of wall mechanics on organ growth. A more concrete question is how differences in wall mechanical properties and osmoregulation of different sections of the maize leaf affect growth under abiotic stress conditions such as drought (e.g., Dzhurakhalov et al., 2015b). The Maxwell module is particularly suitable since, instead of the artificial representation of wall yielding and turgor in VirtualLeaf, real biophysical properties are used such as elasticity and viscosity.

Whereas, Virtual Plant Tissue offers the speed and flexibility to develop more advanced models for cell wall mechanics, it currently does not allow describing intracellular interactions which potentially play a role in determining cell shape such as those involving the cytoskeleton (Sampathkumar et al., 2014). To describe such processes at high resolution Virtual Plant Tissue is less suitable than finite element methods (e.g., Yanagisawa et al., 2015) or a three-dimensional modeling framework (Boudon et al., 2015).

In line with the modular simulation set-up diverse new algorithmic options are available, like choices for the ODE solver (www.boost.org), random number generators and distributions, and Monte Carlo energy evaluation criteria. It is for instance important for a (stochastic) modeling framework to evaluate the influence of those choices on simulation output and to ensure convergence of the Monte Carlo equilibration (Dzhurakhalov et al., 2015a). For an overview of algorithmic options the reader is referred to the user manual (Chapter 3). In the end all selected model components are organized in a time evolution scheme (time_evolver class). Virtual Plant Tissue provides different readymade choices to the user. For instance selecting the “VLeaf” evolver instead of the “VPTissue” evolver results in each simulation time step terminating with the reaction and transport steps instead of the fast elastic equilibration step.

### Additional features and toolset

**Figure 8:**
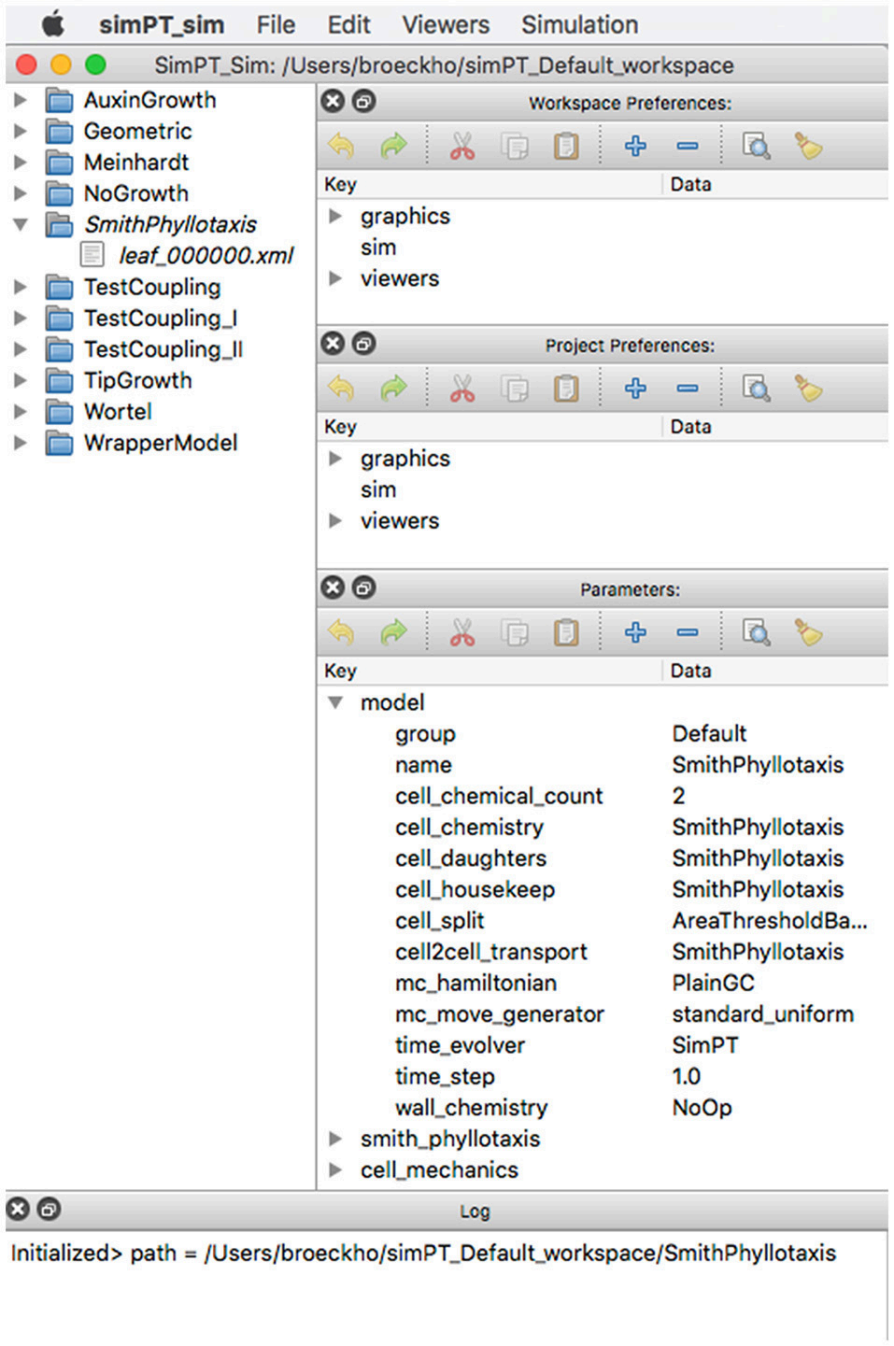
**Screenshot of the Virtual Plant Tissue simulator started with the simPT_Default_workspace containing 11 projects**. Project SmithPhyllotaxis is opened.

**Figure 9:**
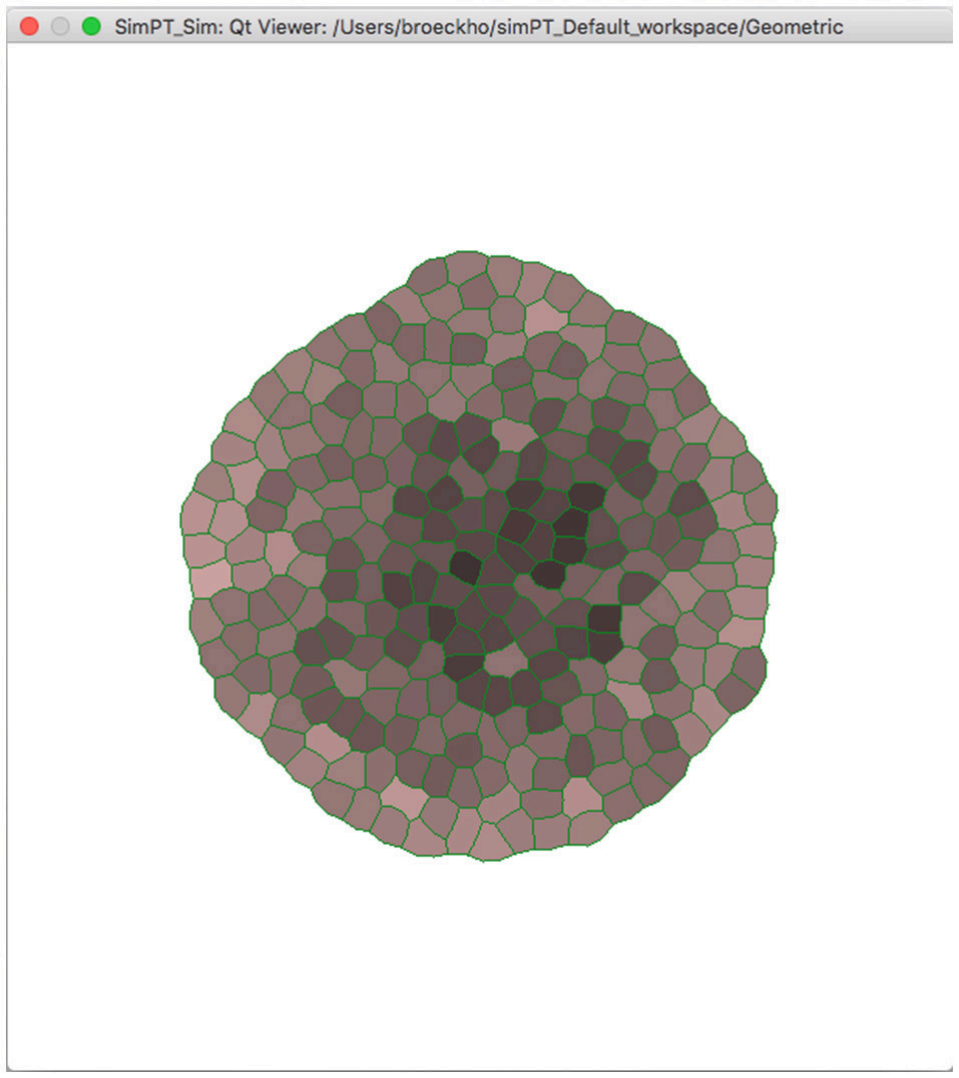
**Screenshot of the Virtual Plant Tissue Qt viewer for the Geometric project in the workspace of Figure 8**.

Simulations are organized into workspaces which consist of projects (directories) comprising the initial data file, simulation output preferences, and accumulated output files. Besides running Virtual Plant Tissue via the command line a graphical user interface is available for users. Figure 8 shows a Virtual Plant Tissue screen shot open at a workspace with several projects (left panel). The top right panel is used to access the workspace preferences determining features such as which I/O viewers need to be enabled, which color scheme should be used, etc. The Parameters panel allows viewing and editing all configuration parameters from the simulation. The Project Preferences panel enables to overrule workspace preferences for a particular project. The bottom panel provides a running log of a project that is open. By virtue of a rigorous Model-View-Controller design it is possible to attach and remove multiple viewers during a running simulation to economize on computational resources. Figure 9 shows a screenshot of the Qt viewer for the Geometric project in Figure 8.

The simulator itself has functionality for converting between XML, compressed XML or HDF5 data formats (Supplementary File 1) and for post-processing simulation output in various graphic (PLY, pdf, png, …) or text (csv) formats. The HDF5 format allows data arrays (practically unlimited in size) to be easily accessed, exchanged and analyzed (a plugin for the Paraview visualization software is included in the source code, a dedicated VPTissue HDF5 file reader for Python, is available on https://pypi.python.org/pypi/PyPTS/0.2.4).

**Figure 10:**
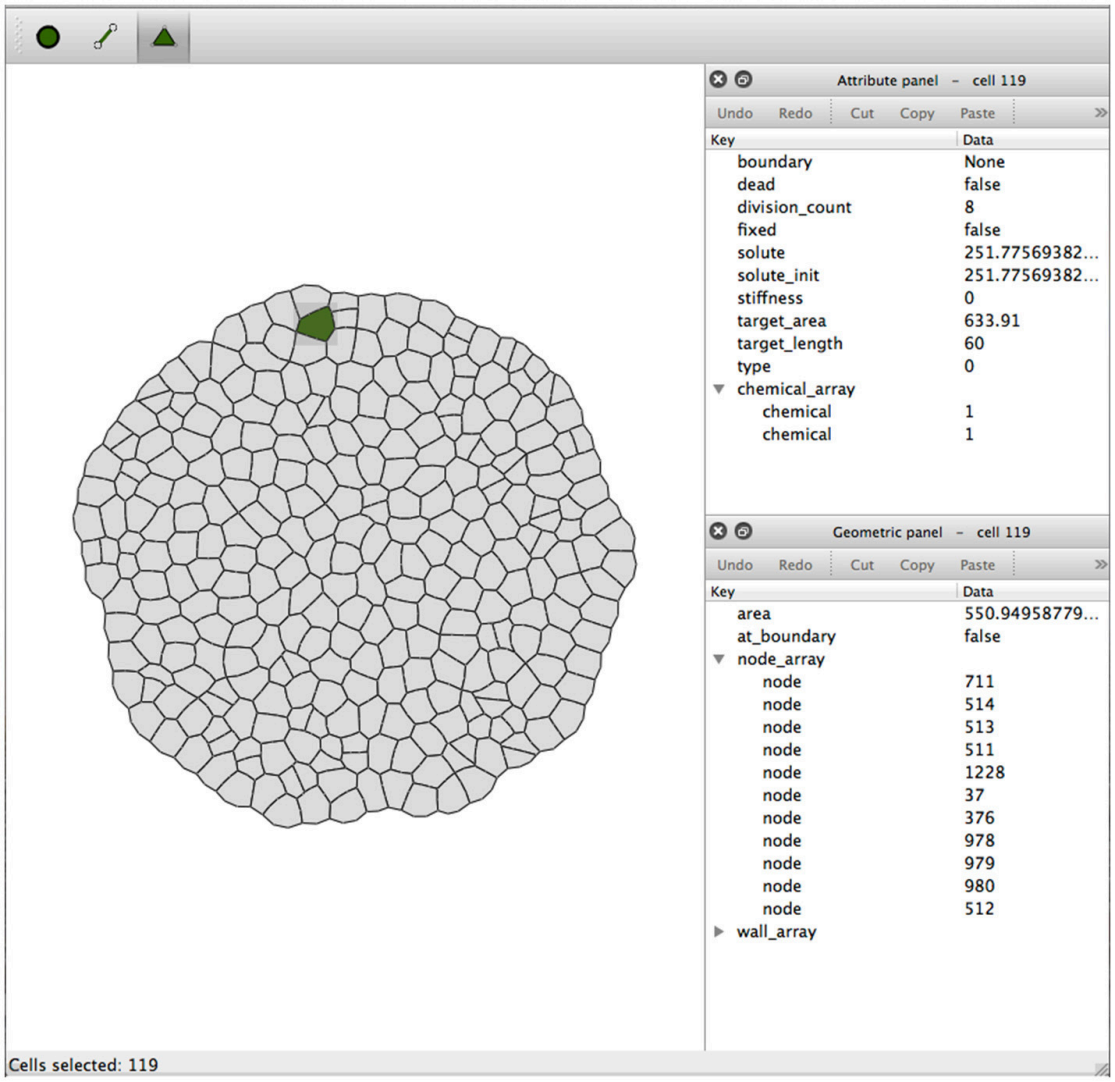
**Screenshot of tissue editor window with the attribute panel and geometric panel to the right**. The Node, Edge, and Cell mode selection buttons are on the top left of the window. These buttons enable type-specific operations like node movement, edge insertion, cell creation, etc. (details can be found in the user manual).

The Virtual Plant Tissue package comes with a toolset comprising a graphical editor (simPT_editor) for XML files. The Tissue Editor is a graphical editor for the Virtual Plant Tissue mesh geometry, the cell, wall, and node attributes and the model and simulation parameters (Figure 10). The application constructs, reads and writes a full XML file, including simulation parameters and mesh data. It is possible to load an image that can be used as a template to draw cell meshed based on microscopic images of plant tissue. Detailed information on the graphical interface can be found in the user manual (src/doc/latex_user_man/UserManual.pdf, Chapter 3).

Virtual Plant Tissue also comes with a parameter exploration tool (simPT_parex) which allows running and monitoring a parameter sweep calculation (with different sampling strategies) on a compute server. After connecting to a server one can start a new exploration or get details on a running task. Several types of exploration can be started: in sweep based explorations one parameter is varied based on a range of values (e.g., Supplementary Figure 4), template based exploration allows varying several parameters at once with a csv file that specifies the parameter combinations. More detailed information can be found in the user manual (src/doc/latex_user_man/UserManual.pdf, Chapter 4, Data Sheet 2).

The build and installation process is tailored to rapid identification of compilation/installation/run-time errors with a high level of abstraction and platform independence allowing easy compilation on Linux, Windows, and MacOS systems. Code documentation is partly automated [the Application Programming Interface (API) documentation in the reference manual is generated via doxygen (www.doxygen.org)].

## Discussion

The Virtual Plant Tissue project grew out of contacts with the authors of the established plant modeling framework VirtualLeaf (Merks et al., 2011). Whereas, in many ways it is an offspring of VirtualLeaf, in other ways it is new and state-of-the-art. It has a totally new codebase (C++11), takes advantage of the multi-core architecture of present day systems and is current in its use of libraries (e.g., Qt4 and Qt5). Moreover, it offers new, biologically relevant features: new models, dynamic models, coupled simulations, and a practical toolset with a tissue editor and parameter exploration tool, which should especially appeal to the aspirant modeler. Thanks to the third party Boost library the ptree data structure was introduced which comes with improved input-output capabilities like the use of HDF5 data files which for instance allows data processing with the open source high performance data analysis and visualization application Paraview or with the *in house* PyPTS toolset. A flexible post-processing feature is also available through the graphical user interface.

A multiscale perspective that focuses on the interplay between cellular and macroscopic studies is expected to become increasingly important for plant biology. Virtual Plant Tissue was specifically developed aiming at modularity, and inter-operability, features which are considered to be important to support modeling efforts in that respect (Baldazzi et al., 2012). With modularity we mean the structuring of code such that it consists of conceptually and functionally coherent entities which have clean and minimal interfaces. This increases readability, facilitates troubleshooting and improves extensibility. In the case of Virtual Plant Tissue this modularity makes it is easier to add new models or model components, as well as to re-use them. It also promotes standardization of models within the framework and facilitates unit testing strategies. Moreover, since the model components are themselves parameters in the input data files, which can be altered during simulation, the model composition can be dynamic in time. Furthermore, any model component can also be built up by reference to other components (a meta-component so to speak) in which case model building does start to look like brick laying. This feature is not available in most other open source plant modeling frameworks. Even in modular frameworks such as the Python based OpenAlea this is less straightforward due to the absence of clean interfaces. Virtual Plant Tissue, like VV or Organism, is written in C++ which remains the standard for scientific computing software due to its performance, robustness to run-time errors, and parallelizability (Barton and Nackman, 1994; Stroustrup, 2013). The easy (parameter dependent) availability of the Boost repertoire of random number generators is especially useful for a (hybrid) stochastic simulator if multiple (parallel) independent runs are required. If we take inter-operability to be the ability for different applications to exchange data and interact, then a number of issues occur. First of all, the diversity of software for plant simulation leads to models implemented in different languages, with different conceptual designs and data representations. Cross-compatibility of software in different environments can also be an issue. Virtual Plant Tissue represents two novel types of solutions. Firstly, an internal interface allows model simulations defined in Virtual Plant Tissue to be coupled in a direct way. A so called boundary condition coupler is provided which uses an extra input file specifying which cells of the respective models are interacting via the internal memory. This solution avoids the above issues, at the cost of possible reimplementation in Virtual Plant Tissue. In this way static boundary conditions, as found in many models, can be replaced by dynamic models. The second solution consists of a so called wrapper class with a limited set of interaction methods which provides an external coupling interface for other applications developed in C++, Java, or Python. The glue code is already present in the Virtual Plant Tissue code base. In this case, only some light re-engineering of both models will be required but not a full re-implementation. A concrete and potentially useful application could be to couple Virtual Plant Tissue based mechanistic tissue growth models (like a root tip or a leaf) to the corresponding parts of a functional-structural plant model (for instance from OpenAlea).

We have already discussed above some limitations of Virtual Plant Tissue. Unlike lattice based models (Grieneisen et al., 2007; Mähönen et al., 2014) or vertex-based models with cell partitioning (Abley et al., 2013) intracellular hormone gradients or other subcellular phenomena (like cell compartments which can affect hormone availability; e.g., Band et al., 2012) cannot be described yet with Virtual Plant Tissue. Unlike OpenAlea which can offer a so called visual programming environment (VPE), model implementation in Virtual Plant Tissue requires C++ programming skills. Obviously, no three-dimensional tissue representations can be done. At this point parallelisation of the simulator is not available as an option yet, but this is envisaged for the next release.

## Author contributions

All authors made substantial contributions to the conception, design or analysis of the work. All authors contributed to drafting and revising the work, final approval of the version to be published, and are accountable for all aspects of the work, ensuring that questions related to the accuracy or integrity of any part of the work are appropriately investigated and resolved.

## Funding

This work was supported by a concerted research activity grant (GOA) “A Systems Biology Approach of Leaf Morphogenesis” of the University of Antwerp. DDV was supported by the Belgian Science Policy Office by a MARS Inter University Attraction Poles project (IAP7/29). SS was supported by an IWT grant.

### Conflict of interest statement

The authors declare that the research was conducted in the absence of any commercial or financial relationships that could be construed as a potential conflict of interest.
