# Supplementary material for: Virtual Plant Tissue: Building Blocks for Next-Generation Plant Growth Simulation
Source: Front Plant Sci. 2017 May 4;8:686. doi: 10.3389/fpls.2017.00686 (PMC5415617; doi:10.3389/fpls.2017.00686)

# *Supplementary Material*

# Virtual Plant Tissue: building blocks for next-generation plant growth simulation

**Dirk De Vos*, Abdiravuf Dzhurakhalov, Sean Stijven, Przemyslaw Klosiewicz, Gerrit T.S. Beemster*, Jan Broeckhove**

*** Correspondence:** Corresponding Authors: dirk.devos@uantwerpen.be; gerrit.beemster@uantwerpen.be

## 1 Supplementary Data

**Supplementary File 1.** LateX generated pdf of user manual with hyperlinks (cf. Data Sheet 2)

**Supplementary Text 1.** Descriptions of the coupled models

Two sets of models were defined and implemented to demonstrate internal (VPTissue-VPTissue) coupling and external (VPTissue-python/PyPTS) coupling. This text complements the model descriptions in the legends of Figures 5 and 6. It should be noted that in the implementation of these models the ‘chemical’ attributes (as found in the input/output files) represent molecule numbers per cell and the differential equations describe changes in those attributes. In the text below the differential equations are transformed to a more conventional and readable form representing changes in the number of molecules per unit area (concentrations).

*1. VPTissue –VPTissue coupling: models TestCoupling_I – TestCoupling_II (Fig. 5)*

All model specific code (components and input files) can be found in src/main/models/Default/ (the files referring to TestCoupling, TestCoupling_I, and TestCoupling_II)

TestCoupling_I and TestCoupling_II are static models so there are no Monte Carlo equilibration and growth or division rules required. Arbitrary units of time (*T*) and length (*L*) were used for the relevant processes. The simulation time step is 60 time units.

Intracellular reactions are described as:

$$\frac{d[chem_0]}{dt} = \frac{1}{A} \cdot k_{prod,0} \cdot$$

The left hand side of the equation represents the change in the concentration (number of molecules per area unit, $N/L^2$) of chemical 0 per time unit. $A$ is the cell size (here area, $L^2$). The production rate

is expressed on a per cell basis (*N*/*T*). Only the TestCoupling_I model produces chemical 0 ($k_{prod,0}$ = 100 *N*/*T*).

To describe cell to cell transport we use the expression $J_{2\rightarrow1} = -P(c_1 - c_2)$ which describes flux $J_{2\rightarrow1}$ from cell 2 to cell 1, with $P$ a permeability constant, $c_i$ the chemical concentration of cell *i*. Intracellular diffusion is neglected in the models.

$$\frac{d[chem_0]}{dt} = \frac{1}{A} \cdot \sum_{walls} l \cdot P_0 \cdot \Delta[chem_0],$$

$$\frac{d[chem_1]}{dt} = \frac{1}{A} \cdot \sum_{walls} l \cdot P_1 \cdot \Delta[chem_1].$$

These equations describe the change in the chemical concentrations of each cell based on the summation of all passive transport flows over all its walls. These flow terms depend on a permeability constant of the chemical *i* $P_i$ (arbitrary high values were given that lead to quasi uniform concentrations of chemicals over the tissues: $P_0$= 300, 30 *L*/*T* and $P_1$=1000, 100 *L*/*T* for TestCoupling_I and TestCoupling_II, resp.), the concentration difference over the wall ($\Delta[chem_i]$), multiplied with the wall segment length $l$. The summed flow terms are divided by the cell's area to determine the chemical concentration change. To demonstrate flexibility of the coupling algorithm the identity of the transported chemical was defined differently in TestCoupling_I (chemical 0), and TestCoupling_II (chemical 1).

The coupling is defined in the input file of the TestCoupling model which identifies the two coupled models, the type of coupler (ExchangeCoupler), an arbitrary direction for the coupling (from TestCoupling_I to TestCoupling_II) the identity of the transported chemical in both models and the coupled cells (cell 28 to cell 0, cell 29 to cell 1, cell 30 to cell 2, cell 31 to cell 3). Each of the cell to cell couplings also has an associated transport constant ('diffusion'). The same equations are used as for transport in the individual tissues, but now using coupled cell concentrations, which are fixed during the coupling time interval.

*2. VPTissue – python (PyPTS): models 'leaf' and 'root' (Fig. 6)*

The following equations describe the chemicals' concentration (number of molecules per unit area, $N/L^2$) dynamics through production, degradation, and transport in the individual models as well as transport between the models. As above (1) the units are arbitrary and the same expression for passive transport is used. Unlike for the previous models, the chemical production for these models is not on a per cell basis, but on a per area basis (this enables a smooth increase of chemical production with root growth). The time step is 1 time unit (*T*).

$$\frac{d[chem_0]}{dt} = \frac{1}{A} \cdot \sum_{walls} l \cdot P_0 \cdot \Delta[chem_0],$$

$$\frac{d[chem_1]}{dt} = \frac{1}{A} \cdot \sum_{walls} l \cdot P_1 \cdot \Delta[chem_1].$$

$$\frac{d[chem_0]}{dt} = k_{prod,0} - k_{\deg,0} \cdot [chem_0],$$

$$\frac{d[chem_1]}{dt} = k_{prod,1} - k_{\deg,1} \cdot [chem_1].$$

Kinetic constants for the leaf model: $k_{prod,0}$ = 1 $N/(L^2T)$, $k_{prod,1}$ = 0 $N/(L^2T)$, $k_{\deg,0}$ = 0.01 $T^{-1}$, $k_{\deg,1}$ = 0.002 $T^{-1}$, $P_0$ = 100 $L/T$, $P_1$ = 100 $L/T$. An important condition is that chemical 0 production is equal to zero if the concentration of chemical 1 is higher than 2 $N/L^2$. The leaf model is not growing and does not undergo cell division.

Kinetic constants for the root model: $k_{prod,0}$ = 0 $N/(L^2T)$, $k_{prod,1}$ = 3 $N/(L^2T)$, $k_{\deg,0}$ = 0.01 $T^{-1}$, $k_{\deg,1}$ = 0.1 $T^{-1}$, $P_0$ = 100 $L/T$, $P_1$ = 100 L/$T$. Cells grow by changing their target area (5% per simulation step) if the concentration of chemical 0 is above 2 $N/L^2$.

Intracellular diffusion was neglected in both models.

During the tissue growth phase, concentrations change due to dilution. The following Hamiltonian ('ModifiedGC') was used for mechanical equilibration:

$$H = \lambda_A \sum_i \left( \frac{a(i) - A_T(i)}{a(i)} \right)^2 + \lambda_M \sum_j \left( l(j) - L_T(j) \right)^2$$

Where indices $i$ and $j$ sum over all cells and polygon edges, respectively, $\lambda_A$ is a parameter setting the cells' resistance to compression or expansion, and $\lambda_M$ is a spring constant. $A_T$ is the cell's target area, $L_T$ the wall element target length. The VPTissue type of time evolution scheme was used (as in Figure 1), with node movement restricted to the y axis (mc_move_generator: 'directed_uniform').

Cells divide if their size is above 400 $L^2$ (with +/- 10 % uniform noise added to avoid synchronous cell division).

The model specific code (components and input files) with a more complete listing of all parameters can be found in src/main/swig_sim/py_WrapperModel for the leaf model and in src/main/models/Default/ (files referring to WrapperModel). The boundary condition coupling is implemented in a different way than above with the lower cell row of the leaf model (cells 28-31) and the upper cell row of the root model (cells 0-3) used as (static) cells that contain the boundary concentration values (fixed during each coupling time interval). In the exchange step the changed concentration values of cells 24-27 of the leaf model are passed to the root boundary cells (cells 0-3), and the values of cells 4-7 of the root model are passed to the leaf boundary cells (cell 28-31). Cells involved in chemical exchange were kept from growing or dividing to keep the cell indices fixed.

**Supplementary Video 1.** Movie of cross-platform root-leaf simulation.

The colour intensities represent chemical concentrations: chemical ’0’ in red, chemical ‘1’ in blue. For more details see the legend of Figure 6 of the main text.

# 2 Supplementary Figures and Tables

## 2.1 Supplementary Table

Supplementary Table 1.

| **Model** | Numer of cells-start | Number of cells-end | CPU1-time (ms) | CPU2-time (ms) |
|---|---|---|---|---|
| Blad | 32 | 1058 | 300627 | 275594 |
| Geometric | 2 | 1005 | 39008 | 26585 |
| TipGrowth | 2 | 1003 | 689004 | 459061 |
| SmithPhyllotaxis | 4 | 1004 | 69894 | 35237 |
| Meinhardt | 16 | 1000 | 144497 | 72432 |

CPU-1: Intel Core i5-5300U CPU @ 2.30GHz, with 8 GB RAM

CPU-2: 4X AMD Opteron Processor 6274, 2200 MHz base frequency, 64 bits, 16 cores, with 8 GB RAM

## 2.2 Supplementary Figures

**Supplementary Figure 1.** Virtual Plant Tissue tissue geometry description

Virtual Plant Tissue is a vertex-based modelling framework which describes plant tissue as a mesh of polygonal cells consisting of vertices connected by (directed) edges that represent cell wall segments. Cells and walls (which consist of one or more wall segments) are endowed with biochemical properties. Cell walls have elastic (reversible) and plastic (irreversible) properties. Central to the algorithm is a generalized energy function or Hamiltonian *H* which is minimized through random displacements of the vertices of the tissue to converge to mechanical equilibrium (details in Merks et al., 2011).

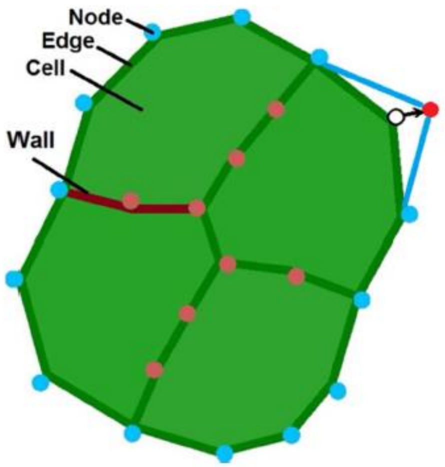

**Supplementary Figure 2.** Screenshots of (A) the ‘SmithPhyllotaxis’ model (B) a leaf model (‘Blad’) and (C) of a (detail of a) root tip model (‘Wortel’) simulation.

The colouring represents increasing morphogen concentrations by increasing saturation of the yellow versus the red colour.  Uncoloured cells have a morphogen concentration below an expansion threshold. The snapshot in (A) corresponds to 668 simulation steps. Cells of the root model contain an arrow representing the PIN transporter polarization. Model simulations with over 1000 cells like the leaf simulation here (snapshot with 1058 cells after 67 simulation steps) can take less than 10 minutes with Virtual Plant Tissue (depending on model and on a standard desktop computer with an Intel Core i5-5300U CPU @ 2.30GHz, with 8 GB RAM). The models are part of the Virtual Plant Tissue distribution (for details see user manual). More precise run time comparisons for specific models and cpu’s can be found in Suppl. Table 1.

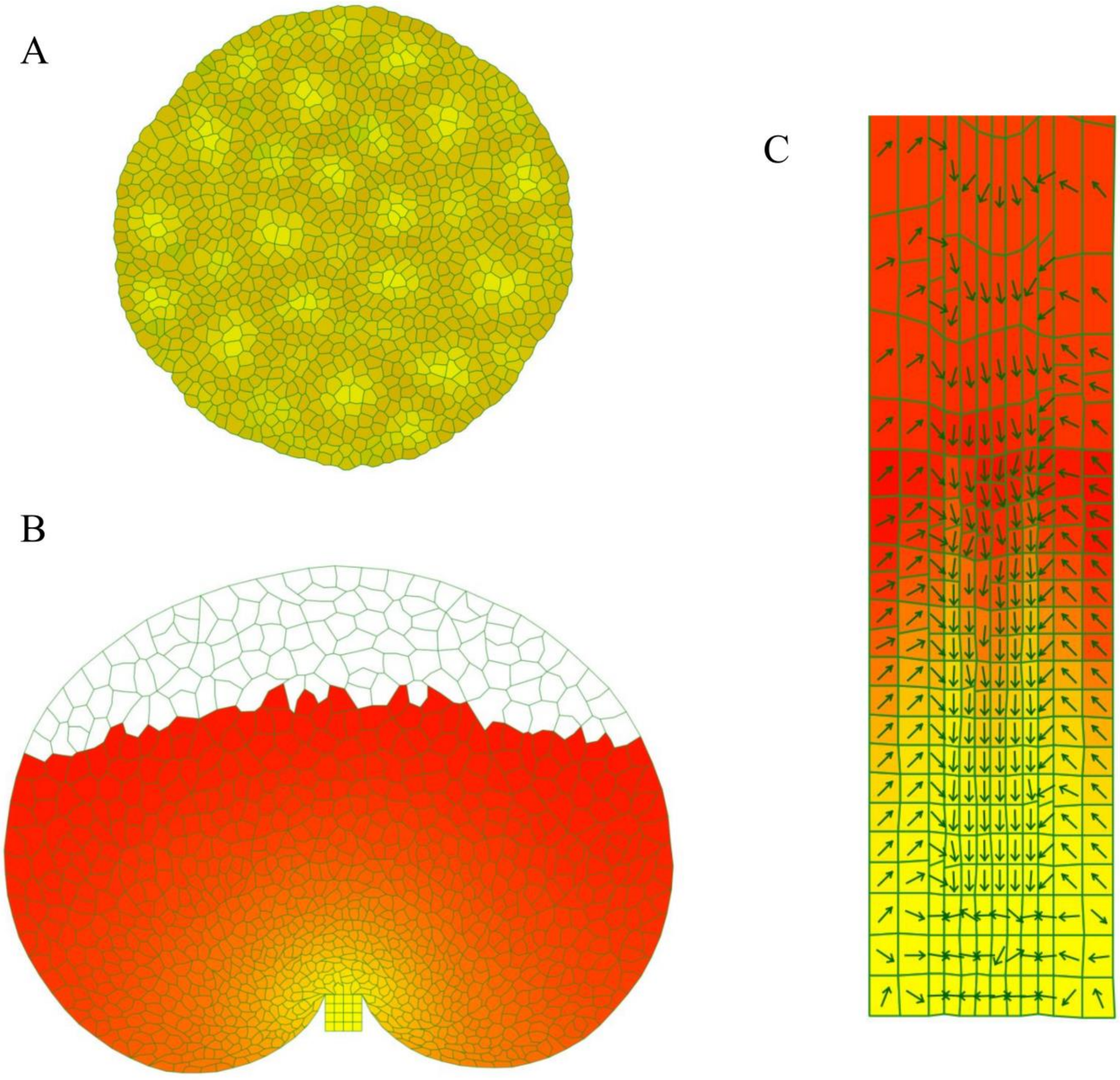

**Supplementary Figure 3.**

Figures A-D illustrate the fundamental differences between the Hamiltonians, in a very simple and direct way, based on the mechanical equilibration of the Geometric model with one small cell and one larger cell which are connected by a wall (A; start of simulation). Only the internal nodes in the connecting wall can move during simulation. After 150 time steps with the original Hamiltonian ('PlainGC') the larger cell already strongly penetrates the smaller cell (B). The same is true for the 'ElasticWall' Hamiltonian (C; the effect is not very pronounced yet due to stronger elastic counterforces). This is no longer the case for the 'ModifiedGC' (D) and the 'Maxwell' (E) Hamiltonians where the smaller cell converges to a stable convex appearance as seen experimentally [Corson et al., 2009]. The more subtle shape difference in D shows that, unlike for the ModifiedGC, through the more realistic biophysical properties of this Hamiltonian it is possible to fine-tune the convexity of the small cell.

Corson, F., Hamant, O., Bohn, S., Traas, J., Boudaoud, A., Couder, Y. (2009) Turning a plant tissue into a living cell froth through isotropic growth. *Proc Natl Acad Sci U S A* 106, 8453-8458.

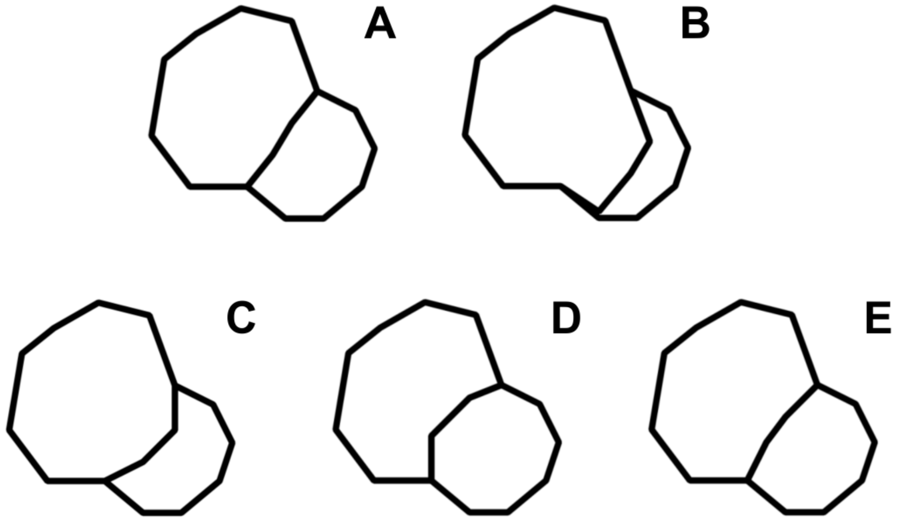

**Supplementary Figure 4.** Screenshot of Parex (Parameter Explorer) dialog for parameter selection with a sweep based range of values (detailed instructions can be found in the user manual).

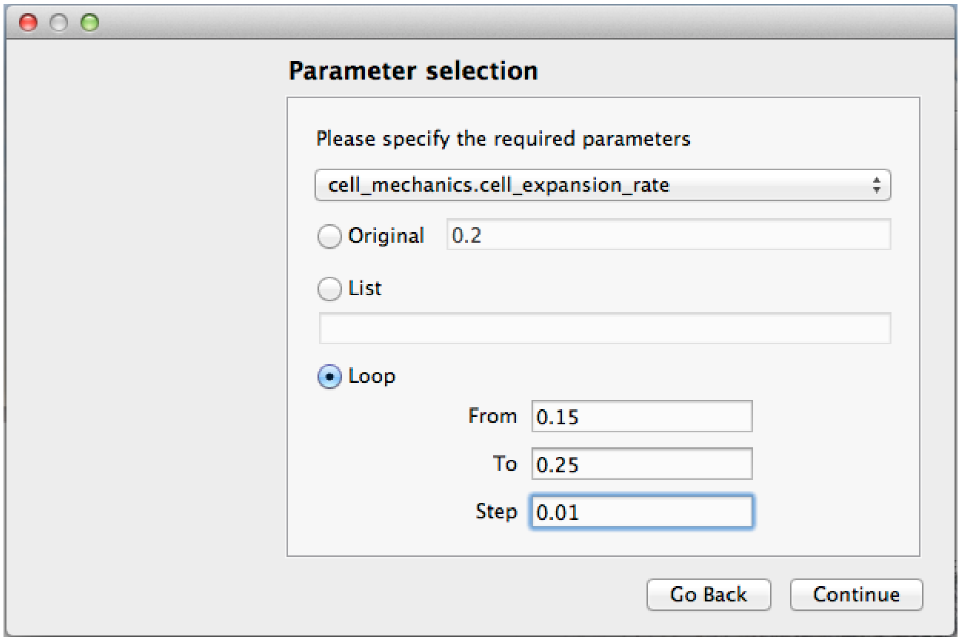

Supplement: Supplementary file 2 [file DataSheet1.pdf]
