# Supplementary material for: Virtual Plant Tissue: Building Blocks for Next-Generation Plant Growth Simulation
Source: Front Plant Sci. 2017 May 4;8:686. doi: 10.3389/fpls.2017.00686 (PMC5415617; doi:10.3389/fpls.2017.00686)
Supplement: Supplementary file 3 [file DataSheet2.pdf]

Universiteit Antwerpen

Virtual Plant Tissue
User Manual

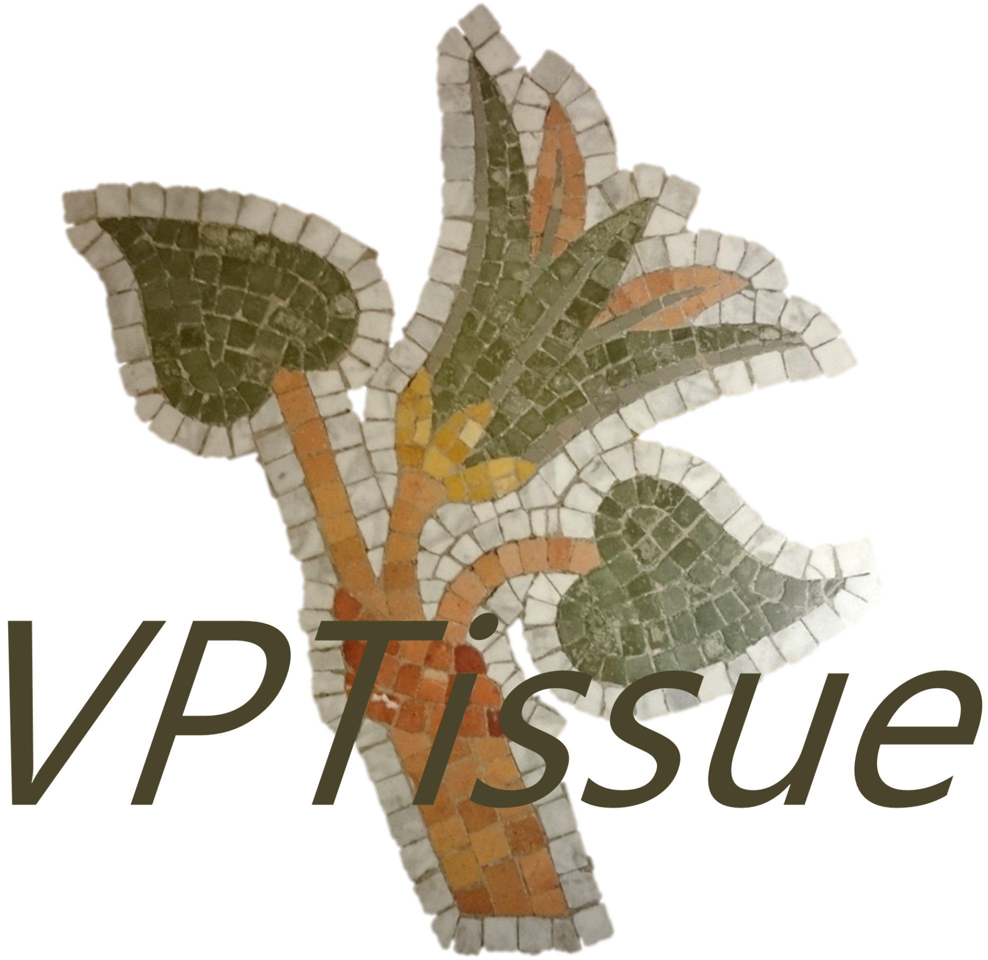

User manual generated on April 22, 2017.

Contents

1 Introduction **2**
- 1.1 The VPTissue toolset . . . 2
- 1.2 Project background . . . 3
- 1.3 Documentation . . . 3
- 1.4 Installation . . . 3
- 1.5 Known Issues . . . 3

2 VPTissue operation **5**
- 2.1 The work shell . . . 5
- 2.2 Running in command line mode . . . 7
- 2.3 Running the simulator interactively . . . 9
- 2.4 Viewers . . . 12
- 2.5 Post-processors . . . 14
- 2.6 Exporters . . . 14

3 VPTissue features **16**
- 3.1 File formats . . . 16
- 3.2 Customizability . . . 17
- 3.3 Dynamic parameters . . . 18
- 3.4 Algorithmic components . . . 18
- 3.5 Models and model families . . . 20
- 3.6 Pre-defined models . . . 21
- 3.7 Language interoperability . . . 23
- 3.8 Coupled simulations . . . 23

4 Tissue Editor **27**
- 4.1 Overview . . . 27
- 4.2 Modes of the editor . . . 28
- 4.3 Selecting items . . . 28
- 4.4 Panels . . . 29

4.5 Graphical editing 29
4.6 Graphical Settings 30
4.7 Toolbar 31

5 Parameter Exploration **32**
5.1 Client 32
5.2 Node and server 35

6 The VPTissue Software **36**
6.1 Code base 36
6.2 Directory layout 37
6.3 Building and testing in Continuous Integration 39
6.4 Platforms 39

7 Programming with the VPTissue framework **43**
7.1 Adding models and components 43
7.2 Adding an attribute 45

A Parameter Dictionary **47**
A.1 Parameters in model 47
A.2 Parameters in auxin_transport 49
A.3 Parameters in Blad0032, Blad0128, Blad0512 (leaf models) 50
A.4 Parameters in cell_mechanics 51
A.5 Parameters in ode_integration 53
A.6 Parameters in random_engine 53
A.7 Parameters in smith_phyllotaxis 54
A.8 Parameters in termination 55
A.9 Parameters in TestCoupling, TestCoupling_I, TestCoupling_II 55
A.10 Parameters in Wortel (root) 56
A.11 Parameters in WrapperModel 57

B Preferences Dictionary **59**
B.1 Introduction 59
B.2 Preferences for graphics: colors_sizes 60
B.3 Preferences for graphics: visualization 60
B.4 Preferences for file format: bitmap_graphics 61
B.5 Preferences for viewer: hdf5 62
B.6 Preferences for viewer: log 62
B.7 Preferences for viewer: logwindow 62
B.8 Preferences for viewer: qt 63
B.9 Preferences for file format: vector_graphics 64
B.10 Preferences for viewer: xml 64

C VPTissue HDF5 file specification **65**
C.1 HDF5 file stucture 66
C.2 Tools 68

CHAPTER 1

Introduction

This manual provides a brief description of the Virtual Plant Tissue a.k.a. VPTissue. VPTissue is a cell based computer modeling framework for plant tissue morphogenesis. It provides a means for plant researchers to analyze the biophysics of growth and patterning.

1.1 The VPTissue toolset

The Virtual Plant Tissue toolset consists of:

Simulator (`simPT_sim`)
: The actual simulator that evolves the organ using the model specified in the input file. It includes capability for conversion between output file formats and for post-processing the simulation output. It can be used interactively, with a graphical user interface, or non-interactively with a command line interface.

Tissue Editor (`simPT_editor`)
: A graphical editor capable of editing the geometry of the mesh representing the plant organ. It can also be used to edit the attributes of cells and walls. The Editor can only be used interactively with a graphical user interface.

Parameter Explorer (`simPT_parex`)
: Facilitates the study of the parameter dependence of the simulation results by distributing calculations over multiple systems. It consists of a client program that functions through a graphical user interface and a server and a worker programs that operate via a command line interface.

Each of these tools is discussed in some detail in the following chapters.

1.2 Project background

The Simulation of Plant Tissue project grew out out of extensive contacts between the Antwerp group of VPTissue authors and the authors of VirtualLeaf ([1], [2], [3]). In many ways, VPTissue is an offspring of VitualLeaf, in other ways it is completely new and state-of-the-art. It has a totally new code base, takes advantage of the multi-core architecture of present day systems and is current in its use of libraries. More importantly, it introduces new features that are biologically relevant: new models, dynamic models, coupled models.

1.3 Documentation

The VPTissue documentation consists of (a) a user manual in `pdf` format (i.e. this document), and (b) a developer's reference manual in `html` format and (c) inline comments in the source code. The user manual has been written in latex (see `www.latex-project.org`) and is generated with hyperlinks for easy navigation. The Application Programmer Interface (API) documentation in the reference manual is generated automatically from documentation instructions embedded in the code using the Doxygen tool (see `www.doxygen.org`). Additional developer documentation has been written in the doxygen syntax and is included in the reference manual. Figure 1.1 presents the starting page of the API documentation.

1.4 Installation

For instructions on how to install VPTissue, see the file INSTALL.txt in the root directory. To check dependence on external resources, see DEPENDENCIES.txt in the root directory. To check issues specific to your platform, see PLATFORMS.txt in the root directory.

1.5 Known Issues

As with almost any software application the size of VPTissue there are a number of known issues where the combination of operating systems and third party library and application software has an issue that cannot be addressed. There are a few such issues in VPTissue and they are listed in the file KNOWN_ISSUES.txt in the top level directory of the projects. When it is available a fix or workaround is suggested.

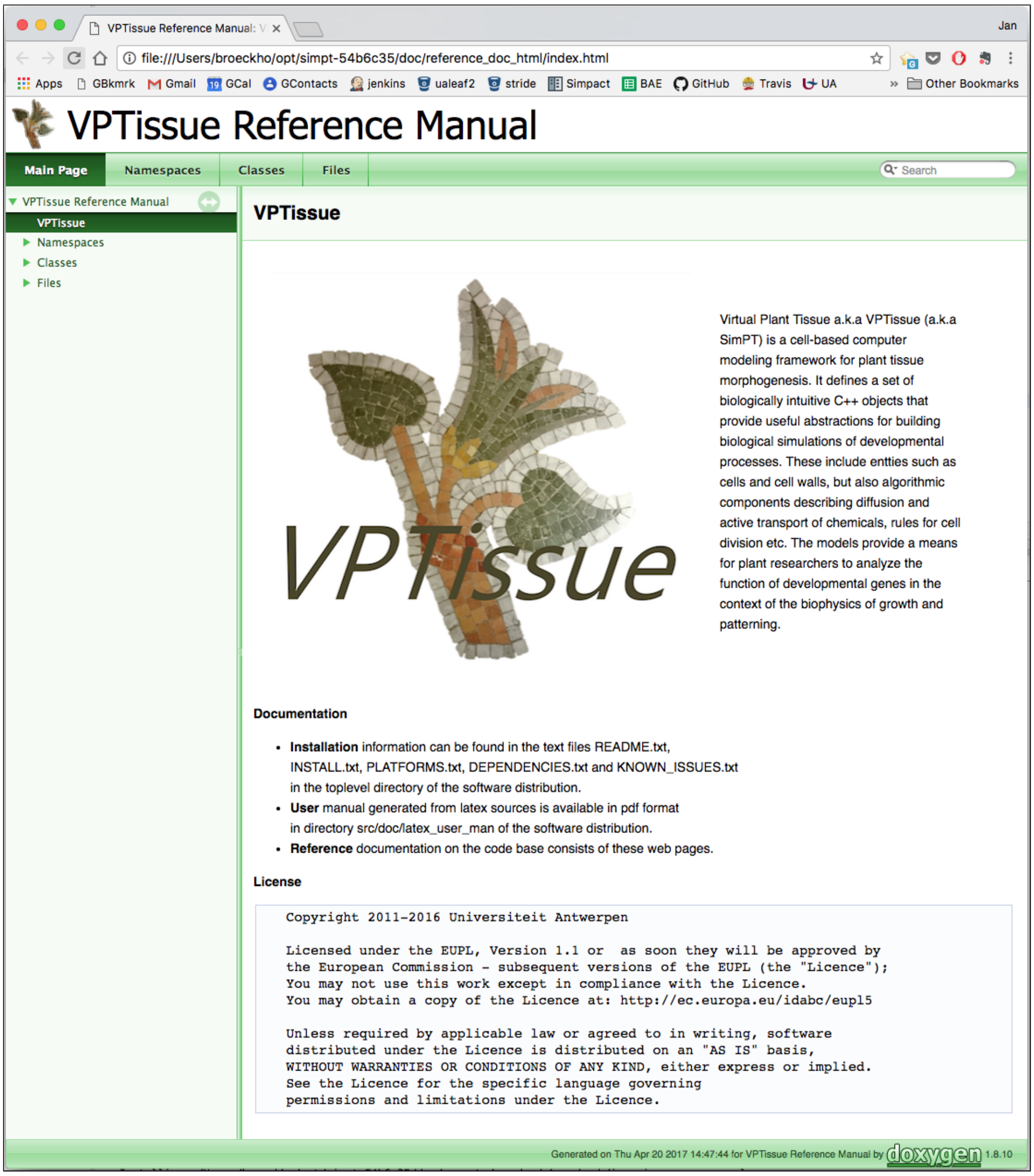

Figure 1.1: Screen shot of the main page of the API documentation. The tabs at the top of page provide access to documentation for namespaces, classes and files.

CHAPTER 2

VPTissue operation

The VPTissue simulator can be used with two different modes of operation:

- via the command line for long running simulations; in this case all simulation parameters defined in the input data are fixed during the program run.
- in an interactive fashion, using a graphical user interface; in this case parameters are initially read from the input data file but can be modified by the user at any time during the simulation through the user interface.

These modes of operation can be mixed as convenient. The simulator also has restart capability, i.e. simulations can be extended starting where the previous simulation run ended. In what follows, we describe the main elements required for setting up the basic work flow.

2.1 The work shell

The core VPTissue simulator (and to a large extent also the parameter explorer and the editor) comes wrapped in a work shell to facilitate organization of the simulation work. The operation of that shell refers to a number of concepts:

- workspaces
- projects
- sessions
- data files
- viewers
- post-processors

These concepts are explained briefly here and in the following sections of this chapter.

Workspace

A workspace is a directory on disk that holds all the resources that you interact with via the simulator. They are:

- The subdirectories, which each define a project.
- The defaults for graphical (`.simPT-gui-preferences.xml`) and command line (`.simPT-cli-preferences.xml`) preferences.
- A descriptor (`.simPT-workspace.xml`) of the workspace state (list of projects) and application state at the time of closing the application. It is used to restore that state when opening the application again.

These resources are all managed via the application.

Project

A project is a directory whose contents represent a single simulation history, that may have been built by multiple runs of the simulator. It contains all resources associated with that history:

- It has states of the simulated system at various times in a number of files (e.g., with the `xml.gz` output format), one for each time point, or in a single file (e.g., with the `hdf5` output format).
- It may contain various post processing files, i.e., files that do not serve as input for a future simulator run, e.g., `png` images of the system.
- Project graphical (`.simPT-gui-preferences.xml`) and command line (`.simPT-cli-preferences.xml`) preferences that override the workspace default preferences. Such preferences customize some simulator actions (e.g., the visualization of results, file format when saving results, at what time intervals to produce output, etc).
- A descriptor (`.simPT-project.xml`) of the project specific application state at the time of closing the application. It is used to restore that state when opening the application again.

A project is either open or closed. The open project is the focus of the simulator actions. Only one project can be open in a workspace at any given time.

Session

A session represents the activation of the simulator core within an open project. Within a session, the simulator can do a single time step, or run, pause and run some more. The project is the exclusive owner of the session and closing the project closes its session. The session only has in-memory structures, an important one being a descriptor of the state of the viewers that observe the simulator and produce output whenever it has taken a time step.

VPTissue data file

A simPT data file contains a full description of the simulation state. If one is using `xml` or `xml.gz` format, the this state information refers to a single point in time and the simulation history is a sequence of such file, one for each time. If one is using `HDF5` format, all states are stored in a single binary file. More information on the formats can be found later on in this annual. The

state information is complete: it contains all relevant data to start or restart a simulation run.

Viewers

We have implemented the Model-View-Controller (MVC) design pattern, a well known design in computer science. This means that code for generating simulator state output to file, output to screen, output to logging files etc. is node built into the simulator. This makes code of the simulator more transparent and adding or changing output feature more flexible and extensible. Instead the simulator connects via signals (e.g indicating a time step has been completed) to (multiple) viewers. Whenever a viewer receives a signal it processes the signal according to its own logic. A file log viewer will post a message to a log file. A graphical viewer will update the on-screen image of the plant system being simulated.

Post-processors

As the name indicates the post processor are to be used after the simulation has been run. The produce post-processing output, e.g. a sequence of images of the simulated system in `png` format. The reason for making this a post-processing activity is twofold. Firstly, it simplifies programming logic. Secondly, when generating images of a growing organ, a scale must be set and often one knows the appropriate scale only at the last simulated time step.

2.2 Running in command line mode

The operation of the simulator from the command line is fairly self-documented. When one starts the executable with 'simPT_sim -h' or 'simPT_sim --help', one obtains the following explanation:

```
USAGE:
   ./simPT_sim  [-h] [-l] [-m <cli|gui>] [--] <> ...

e.g.
./simPT_sim --mode cli -w /path/to/workspace -p project_name

Where:

   -h,  --help
     Displays usage information and exits.

   -l,  --list-modes
     List the available modes

   -m <cli|gui>,  --mode <cli|gui>
     The application mode

   --,  --ignore_rest
     Ignores the rest of the labeled arguments following this flag.
```

```
  <>  (accepted multiple times)
    application arguments
```

It indicates you should use a 'simPT_sim -m cli' or 'simPT_sim --mode cli' to activate the command line mode of the simulator. If one subsequently executes with the help option, namely 'simPT_sim -m cli -h', one obtains the following information:

```
USAGE:
   ./simPT_sim -m cli  [-z <HDF5|XML|XML.GZ>] [-o <BMP|CSV|CSV.GZ|JPEG|PLY
                       |PDF|PNG>] [-c <HDF5|XML|XML.GZ>] [-t <>] [-w
                       <WORKSPACE PATH>] [-p <PROJECT NAME>] [-f <TISSUE
                       FILE>] [-s <NUMBER OF STEPS>] [-q] [-r] [--]
                       [--version] [-h]

Where:

   -z <HDF5|XML|XML.GZ>,  --input-format-filter <HDF5|XML|XML.GZ>
     Only use a specific input format

   -o <BMP|CSV|CSV.GZ|JPEG|PLY|PDF|PNG>,  --postprocess <BMP|CSV|CSV.GZ
      |JPEG|PLY|PDF|PNG>
     Postprocess mode (no simulation): Postprocess existing files in
     workspace.

   -c <HDF5|XML|XML.GZ>,  --convert <HDF5|XML|XML.GZ>
     Convert mode (no simulation): Convert existing files in workspace.

   -t <>,  --timestep-filter <>
     Filter timesteps to convert, (list of) ranges are accepted, e.g.
     "200-300,600".

   -w <WORKSPACE PATH>,  --workspace <WORKSPACE PATH>
     Path to workspace

   -p <PROJECT NAME>,  --project <PROJECT NAME>
     Name of project

   -f <TISSUE FILE>,  --file <TISSUE FILE>
     Tissue file in project

   -s <NUMBER OF STEPS>,  --stepcount <NUMBER OF STEPS>
     number of steps

   -q,  --quiet
     Quit mode (no output)

   -r,  --revision
     Revision identification

   --,  --ignore_rest
     Ignores the rest of the labeled arguments following this flag.

   --version
     Displays version information and exits.
```

```
-h,  --help
  Displays usage information and exits.
```

2.3 Running the simulator interactively

Figure 2.1 shows a screen shot of VPTissue open at a workspace containing 21 projects. The interface presents a number of panels. The panel on the left shows the projects in the workspace and their simulation data files. The top right panel is used to access the workspace preferences determining features such as which i/o viewers need to be enabled, what color scheme must be used and so on. This panel must be opened explicitly via the `Edit` pull down menu. The panel titled "Parameters" allows you to view and edit all configuration parameters of the simulation. The changes you make take effect in the time step following the edit. The panel "Project Preferences" allows you to overrule workspace preferences for a particular project. The bottom panel, which appears only when a project is currently open, provides a running log.

To start a simulation in interactive mode, using the graphical user interface, one has to double-click the simulator icon (or execute './simPT_sim' at the command prompt). Following this one can select a workspace, or if one had in a previous session accepted a particular workspace as default, opens that workspace. Then simply open a project in your current workspace and select an input file among those listed. Double-click the file to initiate a simulation session with this file as its starting point. Click 'run' in the *Simulation* dropdown menu or press the 'R' key, to let the simulator start the time propagation. To stop the time propagation one can again press the 'R' key or select stop from the drop down menu. It is also possible to execute just a single step of the simulation by clicking 'single step' in the same drop down menu or by pressing the 'S' key.

2.3.1 Dynamic parameters

All simulation parameters can be changed dynamically, i.e. every change takes effect at the start of time step following the change. This includes the selection of the model and of the time evolution algorithm. This change can be effected interactively at the discretion of the user or it can be a computed change, i.e., effected by the simulator when certain conditions are met such as number of cells exceeds a threshold or simulated time reaches a value.

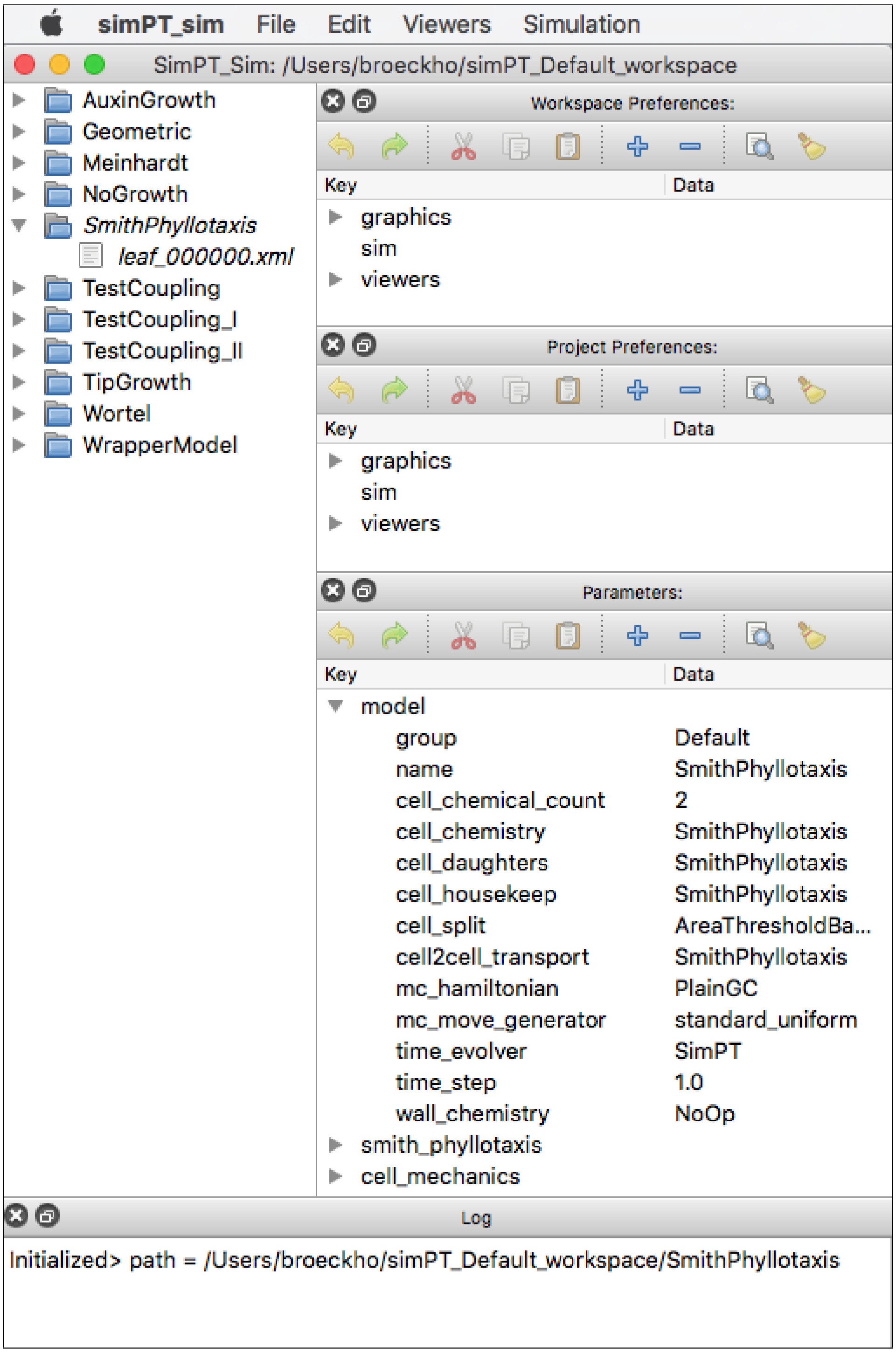

Figure 2.1: Screen shot of the simulator started with the simPT_Default_workspace, that contains nine projects (left pane). Project SmithPhyllotaxis has been opened and the editor panes for workspace and project preferences and for simulation parameters have been opened. The pane at the bottom logs the important events.

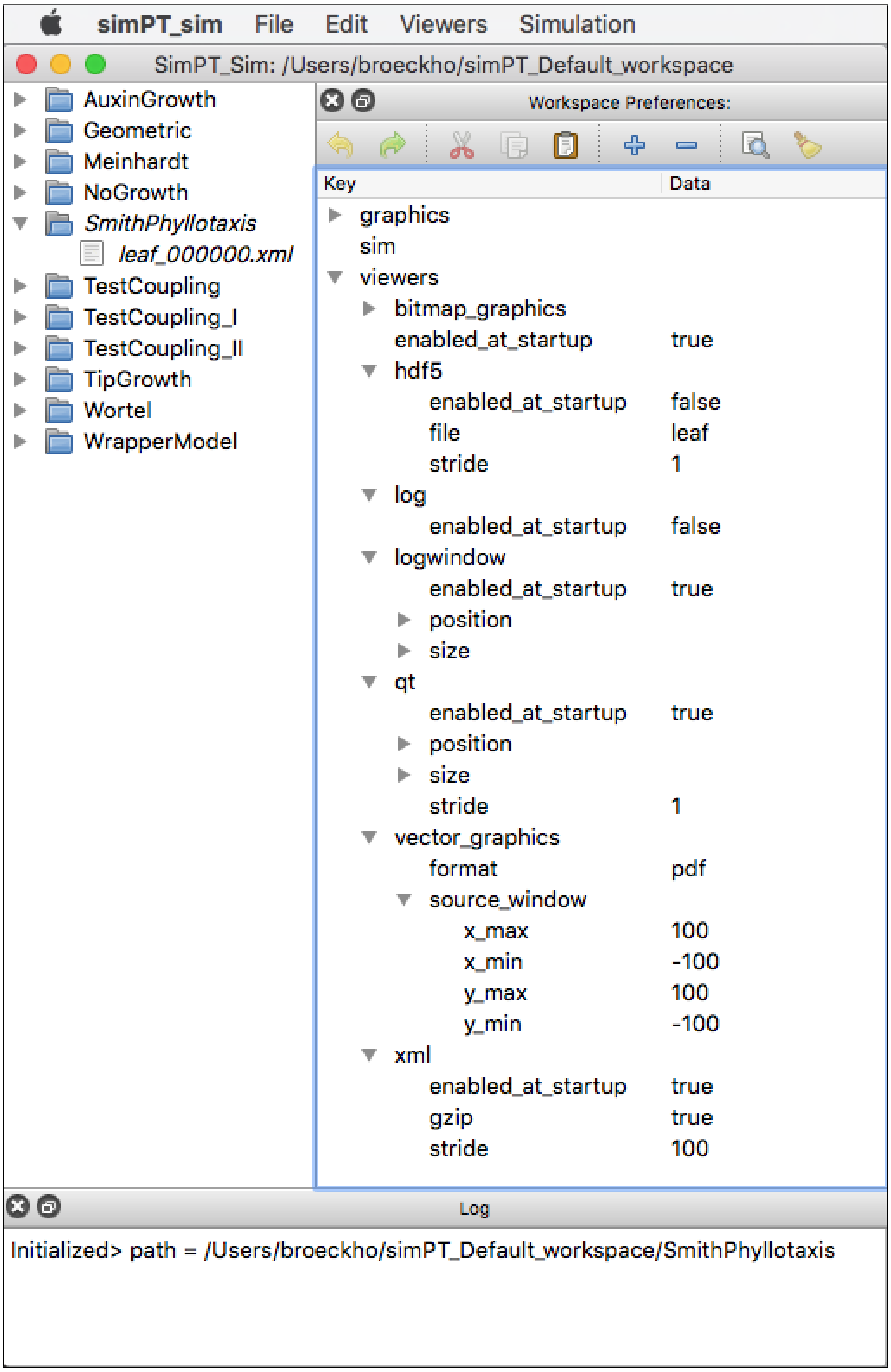

Figure 2.2: Screen shot of the simulator started with the simPT_Default_workspace. Project SmithPhyllotaxis has been opened and the editor pane for workspace preferences is open. The viewers item and most of its sub items have been expanded.

2.4 Viewers

2.4.1 Multiple viewers

A viewer is a component that produces output at every simulation step, while the simulation is running (as opposed to post-processors, that produce output *after* the simulation run. It is possible to attach multiple viewers to a running simulation and allow them to be individually enabled or disabled and to have individual preferences for stride and so on. Enabling and disabling viewers is dynamical, i.e., can be done during simulation. This is not only convenient but also relevant to performance, as image generation and output to file are computationally intensive activities.

2.4.2 Viewers

The viewers allow one to present a view of the simulation log, the tissue images and the simulation data files. Viewers can be activated and deactivated via the `Viewers` pull down menu. The following Viewers are currently available for selection:

- XML File viewer: writes simulation output to separate xml simulation data file for each relevant time step.
- HDF5 File viewer: writes simulation output for all relevant time steps to the same HDF5 file.
- QT window: allows the user to observe the tissue image in a separate QT Viewer window, as shown in Figure 2.3, in this case with a color coding that reflects the relative size of the cell.
- Log dock window: lets user observe a running log in the bottom 'Log' panel, which appears only when a project is currently opened. It shows the path of the running project, running steps, time and the current number of cells.
- Log console: logs extensively to the console from which the application was started.

2.4.3 Preferences for viewers

Preferences that customize the action of a viewer can be edited in the panel "Workspace Preferences" (the edits will apply to preferences of all projects) or in the panel "Project Preferences" (the edits will apply to the open project only). Project preferences may have a special value `$WORKSPACE$` that indicates the corresponding workspace preference is to be used.

An example is shown in Figure 2.2 for workspace preferences. It indicates for instance that in this case the output file will be compressed ("`gzip`" is `true`) and XML output is written to file at every hundred time steps ("`stride`" is `100`).

Every viewer and the set of viewers as awhole has an "`enabled_at_startup`" attribute that does what the name suggests: activating the viewer at start up if set to `true` or not if set to `false`. Activating or deactivating a viewer during a simulation run can be done via the '`Viewers`" pull down menu.

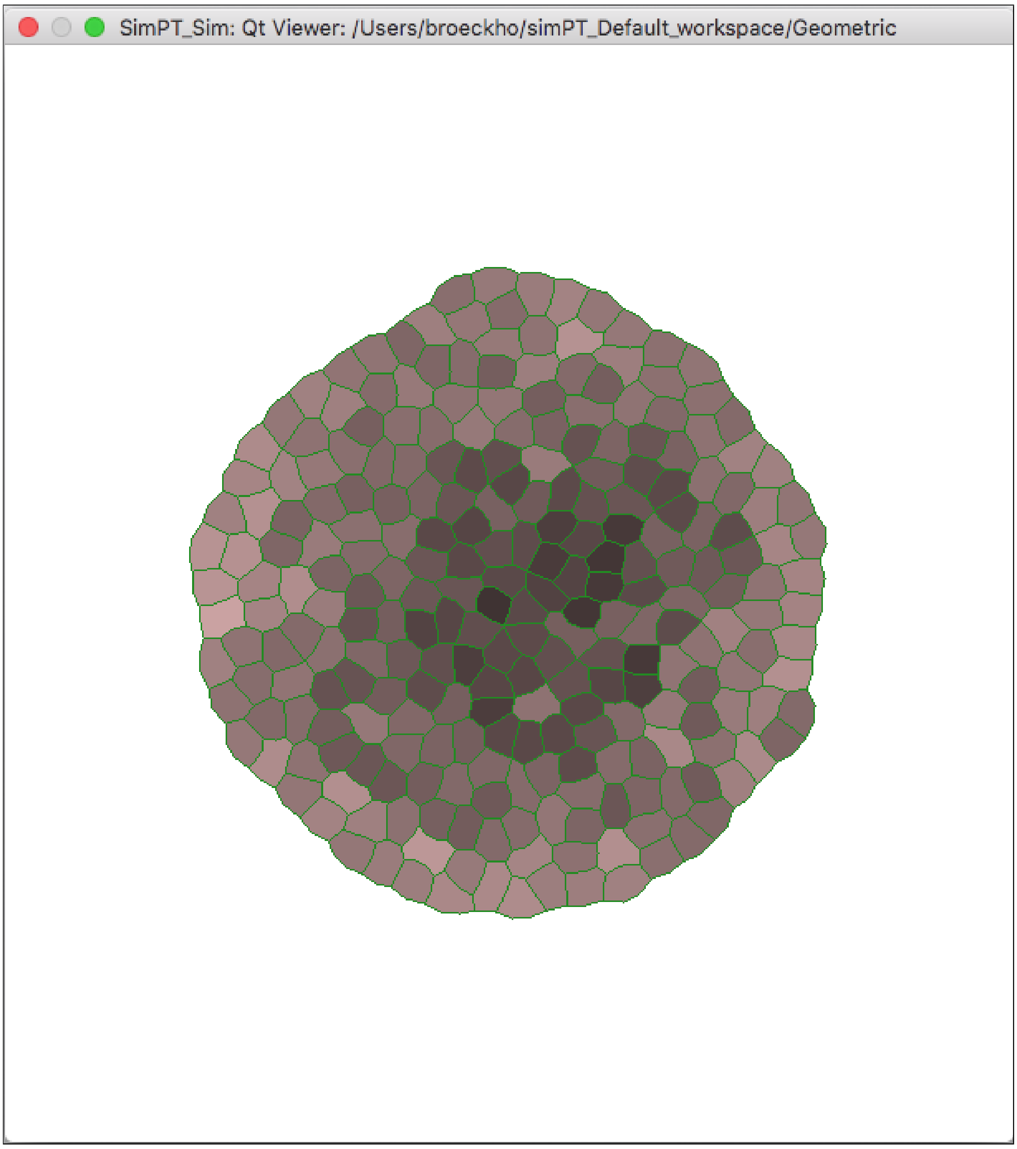

Figure 2.3: Screen shot of Qt screen viewer for the Geometric project in the workspace of 2.1. The color scheme is user defined and reflects the relative size of the cell.

2.5 Post-processors

2.5.1 Post-processing

Files can be postprocessed after simulation by right-clicking on a (closed) project folder and choosing the “Postprocess...” option, after which a dialog will pop up. You can choose different steps included in different files by marking them in the list. For convenience, you can filter steps as well, by specifying a series of comma-separated ranges of the format ‘start-stop:step’ in the regex search field. The files in which to search can be specified as well in the file search field. Once the desired steps have been marked, you can select the export format (BMP, CSV, JPEG, PDF, PLY or PNG), choose a path where the exported files should be stored, and specify a prefix for the files that will be generated. The filenames of the exported files will have the format `<prefix>_<timestep>.<extension>`.

2.5.2 Preferences for viewers

Preferences that customize the action of a post-processor can be edited in the panel “Workspace Preferences” (the edits will apply to preferences of all projects) or in the panel “Project Preferences” (the edits will apply to the open project only). Project preferences may have a special value `$WORKSPACE$` that indicates the corresponding workspace preference is to be used.

For graphical export (BMP, JPEG, PDF and PNG), the preferences subtree `graphics` will be used. Additionally, the preferences sub trees `viewer.bitmap_graphics` and `viewers.vector_graphics` are used for bitmap graphics export (BMP, JPEG and PNG) and vector graphics export (PDF), respectively. See Appendix B for an overview of those preferences.

It is advisable to match the width and height of the exported graphics to the dimensions of the simulated tissue at the last timestep (assuming that the tissue has it’s largest size at that time). The width and height for the images in the graphical post-processing can then be specified in the qt preferences subtree. If you leave these parameters unspecified, the images that are generated in the post-processing will modify the scale as the simulated tissue grows to make the tissue fit into the standard output width and height. To start the conversion, just click the convert button.

2.6 Exporters

During interactive running of the project some interesting states or dynamic processes in the tissue can be exported/saved for further use or analysis. Use the “Export” option in the “File” menu to export a single file of the current state of the tissue to the desirable format. A dialog will pop up to ask the user a path and file name for the exported file. The exporters support the file formats for viewers (XML, XML.gz and HDF5), as well as those for post-processors (BMP, CSV, JPEG,

PLY, PDF and PNG). The XML or HDF5 files visible in the project panel also can be exported by double clicking them at first, then using the “Export” option.

CHAPTER 3

VPTissue features

The design of Virtual Plant Tissue intends to make it a rich, flexible and extensible environment for developing simulations of Plat Tissue processes. In this chapter we present a brief overview of of the key feature of VPTissue that serve to realize those objectives.

A number of those features relate to the overall design of the interaction between the simulation work shell and have already been discussed in the previous chapter.

3.1 File formats

The use of the MVC pattern in the design of the work shell makes it straightforward to extend the work shell with viewers or post-processors for new file formats. At present recognizes a number of file formats for input and output of the simulation data and a number of graphical formats for the post-processing of simulation data.

3.1.1 Formats for input-output

The simulator supports three file formats for input and output of full simulation data (i.e. a dataset that can be used to restart and extend the simulation):

XML a well-known human readable or text based markup format (see http://en.wikipedia.org/wiki/XML)

XML.GZ compressed file of XML format format (see http://en.wikipedia.org/wiki/Gzip); provides typically 80-90 percent reduction in file size

HDF5 a machine readable or binary hierarchical data format often used in data-intensive scientific applications (see www.hdfgroup.org/HDF5/)

HDF5 is a widely used file format in scientific visualisation. It is a portable file format that comes with a high-performance software library that is available across platforms from laptops to supercomputers, with API a.o. for C/C++. It is a free, open source software. On www.hdfgroup.org/HDF5/doc/index.html one finds documentation, specifications and examples. HDF5 provides a significant enhancement in functionality because it enables the use of Paraview (see www.paraview.org), a state-of-the-art scientific visualisation tool. This requires the use of a Paraview plug-in to make the VPTissue HDF5 file structure available to Paraview. This plug-in has been developed. The specification of the HDF5 file can be found in appendix C.

3.1.2 Formats for post-processing

VPTissue supports the following formats for numeric or graphical (both vector and bitmap) output:

BMP a graphics format that provides a bitmap graphics representation of the mesh (see http://en.wikipedia.org/wiki/BMP_file_format).

CSV a human readable format for storing data in a table (see http://en.wikipedia.org/wiki/Comma-separated_values); used to provide numeric output .

JPEG a graphics format (see http://en.wikipedia.org/wiki/JPEG); used to provide a bitmap graphic representation of the mesh.

PLY a human readable format for storing graphical objects that are described as a collection of polygons (see http://paulbourke.net/dataformats/ply/)

PDF a graphics format (see http://en.wikipedia.org/wiki/Portable_Document_Format); used to provide a vector graphic representation of the mesh.

PNG a graphics format (see http://en.wikipedia.org/wiki/Portable_Network_Graphics); used to provide a bitmap graphic representation of the mesh.

3.2 Customizability

A number of choices, represented by parameters in the input data file, can be made to customize aspects of the simulation.

- The set of ODE solvers for the transport equations includes fixed step and adaptive step solvers. The full set of solvers provided by the Odeint package of the Boost library is available (see http://www.boost.org/doc/libs/1_61_0/libs/numeric/odeint/doc/html/index.html. The choice of ODE solver is specified in the input file in the section `parameters.ode_integration` with the parameter `ode_solver`. The tolerances and solver time increments are specified in the same section.

- Tina's Random Number Generators Library developed by Heiko Bauke (see http://numbercrunch.de/trng/ are available. Tina's Random Number Generator Library (TRNG) is a state of the art C++ pseudo-random number generator library for sequential and parallel Monte Carlo simulations. Its design principles are based on a proposal for an extensible random number generator facility, that has become part of the C++11 standard. Contary to the standard C++ RNG's, the TRNG implementation allows for use in the context of parallel calculations. The simulator correctly tracks the state of random generators across restarts to have consistent time evolution, irrespective of the number of restarts. The choice of random generator and seed is specified in section `parameters.random_engine` in the input file.

3.3 Dynamic parameters

The use of event mechanisms in the interaction between the graphical user interface and the simulation core make it possible that all parameters in the simulation input file are dynamic. That is: if a used edits parameters via the parameter edit panel in the GUI, the an event is triggered signalling this to the simulation core. The core in turn will take those changed values into account starting at the next time step and in turn trigger an event that causes the viewers to be aware of these changes and store the changed parameters on file (to be available for a simulation restart or post processing analysis). A parameter change can also be a computed change, i.e., effected by the simulator when certain conditions are met such as number of cells exceeds a threshold or simulated time reaches a pre-set value.

All simulation parameters can be changed dynamically. This includes the selection of the model or of model components and of the time evolution algorithm.

3.4 Algorithmic components

The code for biological processes such as cell division or cell-to-cell transport or the time evolution scheme, has a well-defined set of variation points. These are point in the code where different simulation models require different algorithmic steps.

We use algorithmic components, implemented as function objects (see [4, 5]) to insert code at these points (see more on this in the section on Models).This approach creates a lot possibilities and flexibility to customize and extend the simulator without having convoluted code full of control statements to distinguish the execution flow for each model. The algorithmic components also have well-defined interfaces, make it straightforward for third parties to write their own components.

```

using CellChemistryComponent
= std::function<void (Cell*, double*)>;

using CellColorComponent
= std::function<std::array<double, 3> (Cell*)>;

using CellDaughtersComponent
= std::function<void (Cell*, Cell*)>;

using CellHousekeepComponent
= std::function<void (Cell*)>;

using CellSplitComponent
= std::function<std::tuple<bool, bool,
                           std::array<double, 3>> (Cell*)>;

using CellToCellTransportComponent
= std::function<void (Wall*, double*, double*)>;

using CellToCellTransportBoundaryComponent
= std::function<void (Wall* w,
                      double* dchem_c1, double* dchem_c2)>;

using DeltaHamiltonianComponent
= std::function<double (const NeighborNodes&,
                        Node*, std::array<double, 3>)>;

using HamiltonianComponent
= std::function<double (Cell*)>;

using  MoveGeneratorComponent
= std::function<std::array<double,3>()>;

using TimeEvolverComponent
= std::function<std::tuple<SimTimingTraits::CumulativeTimings,
                           bool>(double, SimPhase)>;

using WallChemistryComponent
= std::function<void (Wall*, double*, double*)>;
```

Listing 3.1: “Code excerpt (edited for inclusion in this manual) of the component interface definitions.” (ComponentInterfaces.h).

3.5 Models and model families

Models are defined by the attributes that are assigned to the cell tissue, to cells, walls, and so on, and by the algorithmic steps taken in the biological processes. The former are data members in the corresponding classes (see section 7.2 for details. The latter are encapsulated in algorithmic components (see the section above and section 7.1). These components deal with cell splitting, cell to cell transport of chemicals, wall chemistry, the time evolution scheme and so forth. A full list of model components in the current VPTissue version with a short description and the range of choices for each component type is provided in the Parameter Dictionary in 5.

VPTissue organizes models into model families. Models within the same family may share components with one another, so as to avoid code duplication. Otherwise models have no component code in common. The model family called "`Default`" plays a special role. It is always built into the VPTissue distribution and if a model definition specifies the use of a component e.g. for the cell_split that is not present within its family of components, then the simulator will look for a cell_split component of that name in the default family. This makes sense because for some component type e.g. the cell_split type or the time_evolver type, many models will use the fairly generic component that is available in the `Default` family. Again we want to avoid copy-paste code duplication with this mechanism.

Model definition is simply a specification of all the names of the components of each type that are to be used by the simulator in executing the model. The names are listed in xml format in the model section of the VPTissue input file. Figure 3.1 shows an example. The section contains the name of the model family and model, the number of chemicals the model deals with, names for the model components of each of the types and the time step to be used.

As the names in the `Wortel` model definition 3.1 suggests, it uses a fair number of components specifically written for this model, but it reuses the `ModifiedGC`, `directed_uniform` and `VPTissue` components that it shares with other models. Though there are two components related the hamiltonian used in the MonteCarlo algorithm (mc_hamiltonian and delta_hamiltonian), these come in pairs and are defined by a single common name.

When one introduces a new model it is a judgement call as to whether it is best categorized as new model of an existing family or whether it warrants defining a new model family. This depends on the amount of component reuse within the family at present and in the future development of the new model.

All of the model specifications are dynamic parameters in the sense of section 3.3. If for instance tissue growth proceeds in distinct phases, then modularity in the model definition allows one to simply specify the change of the relevant component(s) during the simulation.

```
<model>
    <group>Default</group>
    <name>Wortel</name>
    <cell_chemical_count>9</cell_chemical_count>
    <cell_chemistry>Wortel</cell_chemistry>
    <cell_daughters>Wortel</cell_daughters>
    <cell_housekeep>Wortel</cell_housekeep>
    <cell_split>Wortel</cell_split>
    <cell2cell_transport>Wortel</cell2cell_transport>
    <mc_hamiltonian>ModifiedGC</mc_hamiltonian>
    <mc_move_generator>directed_uniform</mc_move_generator>
    <time_evolver>VPTissue</time_evolver>
    <time_step>30</time_step>
    <wall_chemistry>NoOp</wall_chemistry>
</model>
```

Figure 3.1: Model definition for the `Wortel` model of the `Default` model family.

3.6 Pre-defined models

Some of the models in the Default model family serve only for demonstration and have been borrowed from VirtualLeaf ([2, 6]) while others have been used in the context of research. We review the main models briefly:

Geometric
: This primitive model involves cell growth and division and simulates a simple model of callus growth, i.e. an isotropic growth with rapid cell division. The simulation starts with a single cell and cells expand at a constant rate. Cells divide in two equal parts according to a division axis perpendicular to the long axis of the cell (axis of inertia).

TipGrowth
: This model illustrates the interaction between a diffusive morphogen and tissue growth. One of the cells is a continuous morphogen production source. The morphogen diffuses passively through Fick's law. If its concentration exceeds some threshold, cells expand and divide once their area has doubled. At lower concentrations only expansion is possible, at even lower concentrations growth stops altogether.

AuxinGrowth
: This model illustrates an interaction of tissue growth with auxin-driven patterning. Auxin is produced in this model along the perimeter of the tissue. Cell to cell transport is based on the auxin transport model ([1]) and auxin concentrations drive cell expansion inducing localized growth.

Meinhardt

This model is based on the Meinhardt reaction-diffusion model ([7]) and demonstrates leaf venation patterning in growing tissue. Cells differentiate into vascular tissue in response to activator, which is formed by autocatalysis and lateral inhibition.

SmithPhyllotaxis

This model is derived from the Smith phyllotaxis model ([8]) but based on a 2D tissue that is similar to the Geometric model above. Details can be found in [9].

Blad

This is a family of three leaf models which differ in the number of starting cells (32, 128, and 512 cells, resp.). A diffusive morphogen produced in the static leaf stem is crucial for regulating cell proliferation and cell expansion phases. Output data of these models have been fitted to experimental leaf growth data of *Arabidopsis* ([10]).

Wortel

This model describes primary root growth of *Arabidopsis*. The interaction between morphogens auxin and cytokinin is central to formation and regulation of a stable growth zone ([11]).

The next cases are rather model components or combinations thereof. They specify the cell wall mechanics and as such can be used in other models.

PlainGC

As it was based on some geometric constraints, e.g. the cell area constraint, the edge length constraint, etc, it is now called "PlainGC" (Plain Geometric Constraint) model. In PlainGC for the constraint expressions the absolute difference of parameters (areas, lengths) is used and it causes the roles of larger cells to be more dominant than the smaller ones. As a result the smaller cells become less and less significant during equilibration and growth cycle.

ModifiedGC

In the Modified Geometric Constraint ("ModifiedGC") model the relative difference of cell areas is used in the cell area constraint expression, which makes for the equal contribution of both large and small cells.

ElasticWall

The "ElasticWall" model avoids the edge length constraints in Hamiltonian, replacing them by elastic wall term making it additive at wall splitting. Additionally, in this model each wall has its individual and variable rest length given in the XML file ("*rest_length*" in wall attributes), instead of the common and constant rest length of edges ("*target_node_distance*") in "PlainGC" and "ModifiedGC".

Maxwell

The "Maxwell" model represents the viscoelasticity of the cells and cell walls. In this model the turgor pressure term and the elastic wall term are used in Hamiltonian instead of cell area and edge length constraints. The turgor pressure in each cell is represented by the quantity of solute given in XML file ("*solute*" in cell attributes). Contrary to all three previous models, this model is time-dependent: at each time step the quantity of solute in each cell and the rest length of the wall are updated.

Two new models were added to the wall relaxation/yielding model. The first of them is based on the wall length threshold. In this model if the wall length exceeds the threshold value during the wall extension, its rest length is updated by the some rate at each time step until this rest length reaches some value. In fact, this model represents the case when an external force is applied to the tissue. The second wall yielding model is based on the pressure threshold. In this model if the pressure in the cell exceeds the threshold, the rest lengths of cell walls are updated by the some rate at each time step until these rest lengths reach some values. This is a more advanced model for wall yielding in plant cells during their growth.

3.7 Language interoperability

The core simulator has been isolated into a single package. In addition to its internal interface, an adapter has been used to define a clean external interface for the simulator with just five methods, see listing 3.2 . This has made it possible to build wrappers for the simulator in Java and Python. These wrappers, as the name suggests, are native Java or Python classes that can be used in regular Java or Python programming. A wrapper object has the same five methods, but now the methods will forward the calls to the similarly named methods of a corresponding C++ object. Thus, the VPTissue core simulator can be accessed from within Java or Python programs. This makes it possible to develop coupled simulations with VPTissue where one (or more) instance of a VPTissue simulator is coupled to simulators written in Java or in Python.

3.8 Coupled simulations

Multiple instances of simulations can be run in parallel via an internal (coupler) interface thanks to the isolation of the core simulator into a single class (cf. Figure 3.2). Via the ExchangeCoupler class information exchange is restricted to modification of the boundary conditions of a specified set of (boundary) cells. This proceeds after the coupled models have evolved individually in terms of the respective local chemical processes. In the coupling step the chemical concentration of the boundary cells is exchanged (to serve as the boundary conditions in the following simulation

```
namespace SimPT_Sim {

class Sim;
class SimState;

/// Shows whether a time step was a success or not.
enum SimWrapperStatus { SUCCESS, FAILURE };

/// Exceptions are dealt with internally, methods return messages.
template <typename T>
struct SimWrapperResult {
    SimWrapperStatus  status;
    std::string       message;
    T                 value;
};

/// Specialization of SimWrapperResult template for type void.
template<>
struct SimWrapperResult<void> {
    SimWrapperStatus  status;
    std::string       message;
};


/// Interface exposing the simulator to Java, Python, and C++.
class SimWrapper {
public:
    SimWrapper();

    /// Provide sim state in format suitable for i/o.
    SimWrapperResult<SimState> GetState() const;

    /// Provide sim state in XML format serialized to string.
    SimWrapperResult<std::string> GetXMLState() const;

    /// Set sim state.
    SimWrapperResult<void> Initialize(SimState state);

    /// Initialize (path to the input file). This refers
    /// to the one-time setup prior to first use.
    SimWrapperResult<void> Initialize(const std::string& path);

    /// Let simulator take a time step.
    SimWrapperResult<void> TimeStep();

private:
    std::shared_ptr<Sim> m_sim;
};
} // namespace
```

Listing 3.2: "External simulator interface." (SimWrapper.h).

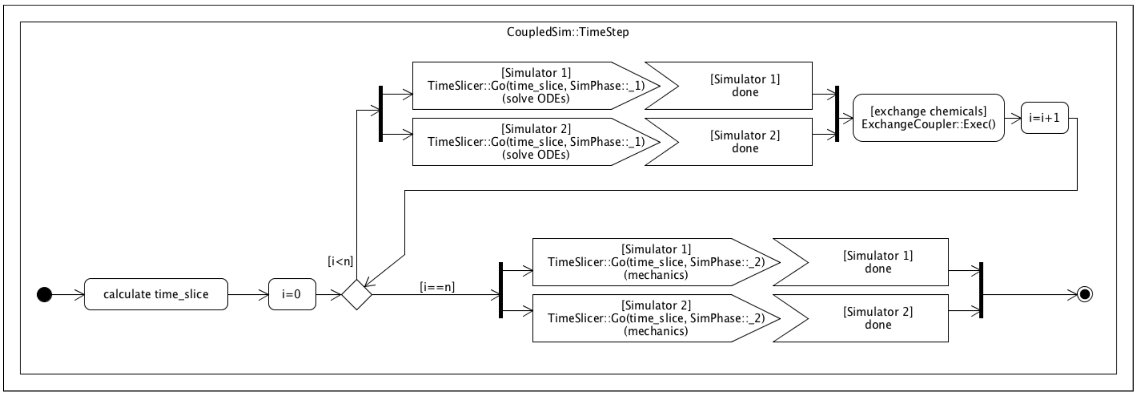

Figure 3.2: Execution flow of coupled VPTissue simulations.

step) and finally the mechanical equilibration is executed for the respective models. To run coupled simulations with VPTissue the interactive (gui) mode has to be used to set up the individual model simulations (projects). A separate project is then initialized which refers to the individual project names and precisely defines the coupling: i.e. which are the boundary cells and how they communicate (coupler type, pairing, transfer kinetics). The parameter `sim_ODE_coupling_steps` determines how tight the coupling is by specifying the number of coupling steps per simulation time step. A working version of such a data file is included in the source code (see 'Test-Coupling' in the resources directory of the Default models) and defines the coupling between models TestCoupling_I and TestCoupling_II.

It is also possible to couple models implemented in a different modelling framework. By means of the Simplified Wrapper and Interface Generator tool (SWIG: see www.swig.org) an interface is created which allows for models coded in other languages such as Python or Java to interact with VPTissue. For that purpose a wrapper class (SimWrapper) is integrated into VPTissue which contains methods that can be called from an external program in order to exchange information as well as coordinate a VPTissue simulation. Figure 3.3 shows an exampe of the interaction of a model defined in Python using the PyPTS toolbox (https://pypi.python.org/pypi/PyPTS) with a model defined in VPTissue. This coupled simulation runs via the command line interface driven by a Python scripts. Precise instructions are given in the source code (src/main/swig_sim/Py_WrapperModel/README.md).

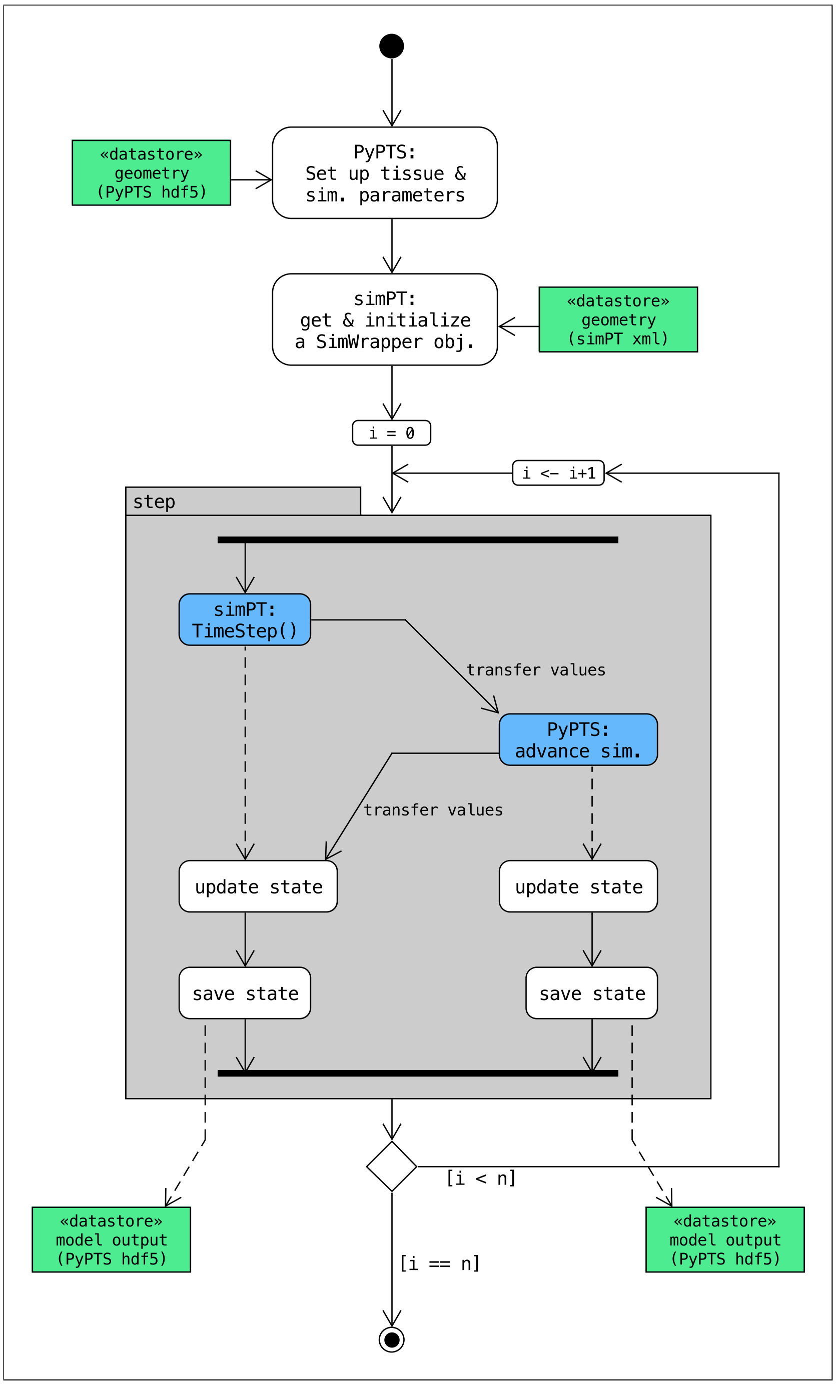

Figure 3.3: Execution flow of coupled VPTissue ('SimPT') and PyPTS simulations.

CHAPTER 4

Tissue Editor

The VPTissue Tissue editor is a graphical editor for the VPTissue mesh geometry and the cell, wall and node attributes. The application constructs, reads and writes a full XML file that includes simulation parameters and mesh data. The parameters are stored on input, can be edited and written on output. The Tissue Editor lets you edit the mesh: geometry and attributes of nodes, cells and walls.

WARNING: the tissue editor cannot process single cell meshes due to a data structure issue forced by backward compatibility. The current implementation of the tissue editor does not have any saveguards against reading a single cell file or making a mesh single cell by deleting all cells but one.

4.1 Overview

When the editor starts up you will notice a menu bar with three pull down menus: *Project*, *Edit* en *View*.

The *Project* menu provides following actions:

- ***New*** lets you initialize a new configuration. It creates a new tissue borrowing the tissue preamble, the parameters and (in the current implementation) the mesh.cells.chemical_count from a template file that you specify in a file dialog. The mesh (taking chemical_count into account) is generated (in the current implementation it is a mesh of two square cells with all nodes, cells and walls having default values for the attributes) and substituted into the tissue. That tissue is then available for editing.
- ***Open*** lets you read sim data (preamble, parameters and mesh) of an existing XML sim dat file. The simulation parameters are stored and included in the output when the edited mesh is written to file.

- ***Save*** lets you write the current tissue to an XML sim data file.
- ***Close*** lets you close the mesh you are currently editing. If you have not saved the sim data data yet, the application will ask you whether it needs to do so.

Depending on your operating system, you will have a ***Quit*** option in the *Project* menu or in the menu with the application name.

The actions accessible through the *Edit* and *View* menus will be addressed in the following sections.

4.2 Modes of the editor

4.2.1 Selection Modes

The mesh display area allows you to edit the mesh graphically. An important feature are the graphical selection modes: *Cell*, *Edge* and *Node*. Note that the mode relates to the type of item you can select to define an operation, rather than the type of item you will operate on. For example, to split a cell you need to select two nodes that define the division axis. Thus the "split cell" action is available in the *Node* selection mode.

One activates a particular selection mode by clicking the appropriate icon in the top left corner of the mesh display area or using the *Mode* option of the *Edit* pull down menu. The icon of the current mode is highlighted in the top left corner. The effect of a mode is that it enables the subset of operations that pertain to that particular type of entity. For more information on the actions available in each mode, see section 4.5.

4.2.2 Display Mode

When you initialize or open a data file, you start in the *Display* mode. If you deactivate the current selection mode by clicking its icon in the top left corner or deselect it in the *Mode* option of the *Edit* pull down menu, you will also revert to *Display* mode. In this mode the mesh is displayed using one of the pre-defined color schemes. A different color scheme (the default is size dependent coloring) can be selected with the *Set color scheme ...* option of the *View* pull down menu. These are the same color scheme options as available in the simulator.

4.3 Selecting items

- You can select an item (node, cell, wall - depending on the current selection mode) by clicking it.
- You can also perform a *bulk selection* by holding down the Shift key and selecting multiple items.
- Finally, you can use the search box present in the toolbar. Specifying a combination (separated by a comma) of ranges of the format 'from-to:step' (with

‘to’ and ‘step’ optional) allows the selection of multiple items based on their ids.

- It is also possible to combine the above selection methods.

4.4 Panels

After you have initialized a new tissue or opened an existing one, you will notice that the application window has an area displaying the mesh and three panels named *Parameters panel*, *Attribute panel* and *Geometric panel*. You can zoom in or out in the mesh display area, for instance by scrolling.

- The first panel allows editing the model and simulation parameters.
- When no item (node, cell, wall) has been selected, the latter two panels display the mesh data.
- When an item has been selected, the *Attribute panel* lets you view and edit the attributes of that item, while the *Geometric panel* lets you view its geometric data. The x, y coordinates of a node can also be edited in the panel; other geometric data cannot.
- When multiple items have been selected, the attribute panel will show all the attributes with the same value for all selected items. Attributes with a different value across the selected items have a question mark in the value area in the panel.
- If you edit any of the attributes in the panel, the new value will apply to all selected items.

4.5 Graphical editing

The first two actions (repositioning nodes and slicing the mesh) are entirely graphical. The other actions are available by using an option in the *Edit* pull down menu.

Reposition node [Node]

A node can be repositioned by selecting it and dragging it to its new position or by editing its coordinates in the geometric panel. The new position is only accepted if it is allowed, i.e., if it does not cause two cells to overlap.

Slicing mesh [Cell]

One can slice the mesh, i.e., draw a straight line and eliminate all cells completely on one side and cut the cells intersected by the line. When in *Cell* selection mode, right click outside the mesh, draw the line by moving the mouse to another point outside the mesh and right click again. Select the part of the mesh you wish to keep. The part on the other side of the line will disappear.

Split edge [Edge]

This action, available in *Edge* selection mode, splits an edge in two halves. This is

the method you use to insert additional nodes or edges in a wall: you split an edge of the wall and then reposition the node to where you need it to be.

Split cell [**Node**]
This action, available in *Node* selection mode, splits a cell. Select two nodes that belong to the cell and that are positioned such that a straight line through the nodes divides the cell in two parts. Choosing the *Split cell* option in the *Edit* menu executes the split. It is of course perfectly all right to first insert nodes with the *Split edge* action for the specific purpose of defining the axis of division.

Create cell [**Node**]
This action, available in *Node* selection mode, creates a new cell at the boundary of the mesh. Select two nodes at the boundary of the mesh, choose the *Create cell* option of the *Edit* menu and position the third node of the new cell by clicking outside the mesh. If you position the third node where it would lead to an illegal construction of the new cell, that position is disregarded. The application forces you to retry until you position that third node at an appropriate position or cancel the action. Of course, you can afterwards format the shape of the new cell by adding a new node (the "Split Edge" action) and repositioning them.

Delete item [**Node / Cell**]
This action removes the selected node or cell.

- When deleting a node, this node has to have a degree of two and can only belong to cells that have at least three other nodes.
- When deleting a cell, this cell has to be located at the boundary of the cell complex and cannot violate the consistency of the mesh upon deletion (e.g., the mesh cannot be split into two separate parts).

Copy attributes [**Node / Edge / Cell**]
This action allows you to copy attributes from a node, edge or cell to another one. First, all target items must be selected. Next, the *Copy attributes* action can be executed, after which the source item must be selected (this can also be an item among the targets). Finally, a dialog will appear where you can specify which attributes should be copied from the source to the targets.

Undo [**Node / Edge / Cell**]
This option, available in all three selection modes, allows you to undo previous actions.

Redo [**Node / Edge / Cell**]
This option, available in all three selection modes, allows you to redo actions you have undone.

4.6 Graphical Settings

In the *View* menu, you will find four graphical settings. The first three actions toggle the visibility of the *Parameters panel*, the *Attribute panel* and *Geometric panel*, allowing a better view on the cell complex. It also will increase performance when handling a large number of nodes, edges or cells, as these panels will not

be updated when invisible. Next, *Transparent cells* allows you to make the cells transparent. The functionality to create a background for the cell complex is also available which allows to draw cell meshes with microscopic images of plant tissues as a template. You can specify an image-file and rotate, scale or translate it as you wish. Of course, this background can be hidden whenever desired. Finally, you can also alter the color model of the *Display* mode.

4.7 Toolbar

A toolbar has been added to allow fast transitions between modes (first three pictograms, respectively modes *Node*, *Edge* and *Cell*) and to quickly cancel an action (fourth pictogram), e.g., splitting a cell. Next to that, the toolbar contains a search option to select items based on their identifiers, by entering a combination of ranges of the form ‘from-to:step’. Toggling the lock button right of this search box prevents discarding previously selected items.

CHAPTER 5

Parameter Exploration

The VPTissue parameter exploration tool allows you to start and monitor a parameter sweep calculation on a compute server. Before starting a parameter exploration, make sure a parameter exploration server and a sufficient amount of nodes are running.

5.1 Client

Before an exploration can be sent or its progress can be shown, it is necessary to first connect to a server. This can be done by clicking “Connect”, after which you can enter a name, ip address and port number. To make this process more convenient, it is possible to save a server (by clicking “Save Server”), which means you can reconnect to a previously configured server. When the right information has been submitted, a connection will be set up.

The central part of the client is the exploration overview, which shows the status of a running exploration to which the user is subscribed. Subscribing to an exploration can be done by clicking “Subscribe” and choosing an exploration you want updates about. Note that when a new exploration is created, you are automatically subscribed to it. You can only be subscribed to one exploration at a time.

A more detailed overview for all tasks that are part of an exploration can be found under “Task Overview”. Here, you can see how many tasks there are, what their status is, the run time for running tasks and the total time taken to finish for completed tasks. It is also possible to cancel a task by clicking “Stop”. In case you want to resend a cancelled task, you can click “Restart” to add this task to the queue. **Note that cancelled tasks are executed *completely* again when they are resent.**

Via “Start Exploration” it is possible to send a new exploration to the server.

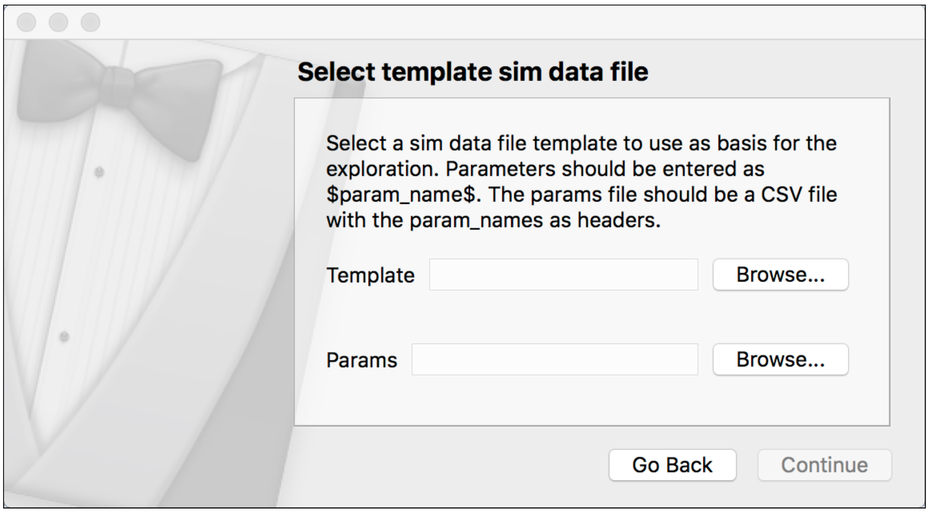

Figure 5.1: The Parex dialog which can be used to start a template based exploration. See listing 5.1 and table 5.1.1 for examples on how to design the "Template" and "Params" file.

The wizard that pops up, gives you the choice to start an exploration based on parameters or based on tissue files. You can also retrieve and edit the last created exploration. By clicking "Delete Exploration", it is possible to remove an exploration from the server, which consequently also removes all its results. This action can not be undone.

5.1.1 Choosing exploration options

There are several types of parameter explorations that can be started.

Sweep based exploration

In a sweep based exploration, a single parameter is varied. You can either choose to vary a parameter based on a range of values (with a 'from' value, a 'to' value and a stepsize) or you can specify the values over which to iterate in a list.

Template based exploration

When you want to vary several parameters at once, it can be useful to create an 'experiment design', to avoid superfluous or redundant calculations. When starting a template based exploration (see figure 5.1), you can specify a tissue file (see listing 5.1 for an example) where the parameters that should be varied are marked and a csv file (see table 5.1.1) in which the combinations of parameters that should be simulated are laid out.

```
...
<parameters>
    <model>
    </model>
    <auxin_transport>
        <aux_breakdown>$param1$</aux_breakdown>
        <k1>$param2$</k1>
        <k2>$param3$</k2>
        <aux_cons>0</aux_cons>
        ....
```

Listing 5.1: An example of how to incorporate the parameters in the tissue XML file. Each parameter name should be enclosed by dollar signs.

param1	param2	param3
0.0001	1	0.1
0.0002	1	0.2
0.0003	1	0.3
...	...	...

Table 5.1: An example of how to design the “params” csv file. The header row should contain the names of the parameters that were defined between the dollar signs in the template file. Each row is a separate experiment that will be distributed to the nodes.

5.2 Node and server

You can start a server by executing `./SimPT_parex -server`. By providing the extra arguments `-n` and `-p`, you can configure the server. `-n` defines the minimum number of nodes the server should have (which by default is 0). Every 30 seconds, the server will check whether this condition is fulfilled. If not, the server will start up a couple of nodes until the condition is satisfied. The argument `-p` allows you to choose a port to communicate with clients (by default this is 8888).

Nodes can be started by running `./SimPT_parex -node`. The node will automatically connect to the server. Nodes write the results of a simulation to the path "vleafspace*NAMEyyyy-MM-dd-HH:mm:ss:zzz*/Simulations*X*/", where *NAME* is the name of the exploration, *yyyy-MM-ddTHH:mm:ss:zzz* the date and time, and *X* the id of the task.

CHAPTER 6

The VPTissue Software

6.1 Code base

At present (spring of 2016) the software stands at approximately 49K lines of C++ application code plus 25K lines of comment (see figure 6.1). About 3.K lines of C++ code are test code in the `src/test` directory. The line counts were gathered with the `cloc` tool (see http://cloc.sourceforge.net/).

In addition to the source code there are a significant number of other artefacts that are involved in building, testing and executing VPTissue. Foremost among them are a number of simulation input files (sometimes referred to as “tissue” files in jargon). These are used for test runs and for populating a workspace template that provides the default project to the users. Other artefacts are configuration files and input files involved in the generation of documentation.

Concerning language conformance, we have taken a forward perspective, aiming to make the code base last for as long as possible. We have used C++11 language constructs (in particular lambdas, range based for loop, auto keyword (see [5, 12]) wherever possible and have continuously refactored the code to use them.

We have taken great care in designing classes, using familiar design patterns ([13]) and using the cppcheck tool (see cppcheck.sourceforge.net) to analyse the code and flag design deficiencies. We have also maximized code reuse by using libraries. A major example is the use of Boost’s (see section 6.3) `ptree` container. This container is tailor made to hold configuration data with a hierarchical structure and provides easy access and input-output to a number of file formats, among others xml.

At the level of program design we have taken great pains to represent domain concepts in well-defined classes. Not only biological concepts (e.g., mesh, cell, wall, edge, node, . . .) but also algorithmic entities such as CellDivider, NodeInserter or

Language	files	blank	comment	code
XML	48	4	25	160507
C++	339	7593	9330	33702
C/C++ Header	419	6179	15141	15609
CMake	56	535	1906	2786
Python	6	117	184	430
Java	4	57	60	245
Bourne Shell	4	39	154	134
make	2	41	27	72
XSLT	1	8	0	36
HTML	2	0	2	26
DOS Batch	1	0	0	2
SUM:	883	14578	26829	213562

Figure 6.1: Line count of source text, documentation, build files, etc. in the `src` directory (excluding external software). Situation of late March 2016.

the various time evolution scheme have been moulded into classes. The use of such algorithmic objects represents current practice in computational programming ([4]).

6.2 Directory layout

The project directory structure is very systematic and is represented in list 1.

Everything used to generate project artefacts is placed in directory `src`:

- code related files (sources, third party libraries and headers, ...) in directory `src/main`
  - for each language the sources in `src/main/"language"...`
  - third party resources in `src/main/resources`.
- documentation files (api, manual, html, pdf and text ...) in directory `src/doc`
  - for each document processing tool a sub directory `src/doc/"tool"...`
- test related files (description, scripts, regression files, ...) in directory `src/test`

Every artefact is generated in directory `target` or its sub directories during the build procedure (see section 6.3). This directory is completely removed when the project is cleaned.

The directory structure is reflective of the large-scale package structure of the VPTissue software, outlined in 6.3.

List 1 The layout of the VPTissue main directory (some directories have been elided to their first level subdirectories). Situation of late March 2016.

```
main
    |-cpp_execs             <--- Main programs to build executables
    |---modes               <--- Modes (GUI, command line, ...) for executables
    |-cpp_parex             <--- Parameter exploration tool
    |---parex_client        <--- Client side of the parex tool
    |---parex_node          <--- Compute node for the parex tool
    |---parex_protocol      <--- Client-server protocol used with parex
    |---parex_server        <--- Server for parex
    |-cpp_sim               <--- Core simulator: biological concepts, algorithms, time evolution
    |---algo                <--- Algorithms for expansion, division, node insertion, ...
    |---bio                 <--- Biological concepts: mesh, cell, wall, edge, node, ...
    |---coupler             <--- Coupled simulations code
    |---fileformats         <--- File formats for use by simulator
    |---math                <--- Miscellaneous mathematical constructs
    |---model               <--- Interfaces to model dependent extension points of the algorithms
    |---sim                 <--- Simulator proper i.e. the simulation driver
    |---util                <--- Miscellaneous utilities
    |-cpp_simptshell        <--- Components that build the user interface
    |---cli                 <--- Command line interface components
    |---converter           <--- Converter between file formats
    |---exporters           <--- Exporters to formatted files
    |---gui                 <--- Graphical user interface
    |---mesh_drawer         <--- Drawing of tissue image
    |---session             <--- SimPT specific features of session creation
    |---viewer              <--- Root viewer construct
    |---viewers             <--- The viewers that are available
    |---workspace           <--- VPTissue specific features of workspace construction
    |-cpp_simshell          <--- Components that build the simulator user interface
    |---common              <--- Common code for user interface
    |---gui                 <--- Graphical user interface for the simulator
    |---ptree               <--- Utilities for dealing with ptrees
    |---session             <--- Simulator session management
    |---viewer              <--- Viewers for simulator
    |---workspace           <--- Simulation workspace management
    |-cpp_tissue_edit       <--- Gui tissue editor tool
    |---generator           <--- Generator for meshes (regular, Voronoi)
    |---editor              <--- Core editor components
    |---slicer              <--- Component with slicing capability
    |-models                <--- Components and resources for each of the model groups
    |---Blad                <--- Components and resources for each of the model groups
    |---Default             <--- Components and resources the Default model group
    |--- ........           <--- Components and resources for model groups you are developing
    |-resources             <--- Resources i.e. non source code artefacts used in build
    |---cmake               <--- CMake modules used for building
    |---data                <--- Third party icons
    |---icons               <--- Icons for desktop use
    |---lib                 <--- External software included at source level in build
    |---make                <--- Makefile template
    |---paraview            <--- Integration of HDF5 data files with Paraview
    |---txt                 <--- Some text files
    |-swig_sim              <--- Java and Python wrapper sources
```

6.3 Building and testing in Continuous Integration

The build system for VPTissue has been engineered with the CMake tool that describes the build steps and build and install artefacts at a high level of abstraction. This leads to build files that are to a high degree platform independent (see http://www.cmake.org/). For those users that do not have a working knowledge of CMake, a front end Makefile has been provided that invokes the appropriate CMake commands. VPTissue builds on Linux/UNIX platforms, on Mac OSX platforms and Windows/MinGW platforms in a number of configuration (see the list in the next section).

From the outset automated tests, including unit tests and scenario tests, have been part of the VPTissue software. We use both ctest, companion to cmake (see https://cmake.org/), and Google's googletest (see https://github.com/google/googletest).

The tests are part of the Continuous Integration develop-build-test cycle ([14], [15]). It is executed with the Jenkins CI server (see jenkins-ci.org). Figure (6.4) shows the status of various CI jobs managed by the jenkins server. Only when all build activities (including generation of documentation artefacts) and tests have been successful, does the new revision of the software get pushed to the project's public repository.

6.4 Platforms

The reference platforms on which the software has been developed and tested through continuous integration are:

- Linux Ubuntu
- Mac OSX
- Windows 7 with MinGW 4.8 (4.8.1-posix-sjlj-rev5.7 available at http://sourceforge.net/projects/mingwbuilds/files/host-windows/releases/4.8.1/32-bit/threads-posix/sjlj/) or MinGW 4.9 embedded in the qt5 distribution.

The compilers and libraries are:

required GCC compiler C++ 4.8 or higher or Clang C++ compiler 3.5 or higher.

required Boost library release 1.53 or higher; only header components are required, libraries are optional

required Qt4 or Qt5 library, specifically Core, GUI and Network components

optional HDF5 1.8 library or higher to have the HDF5 functionality

optional SWIG 2.0 interface compiler or higher to generate the Java and Python wrappers for the core simulator

These are all highly regarded and active C++ library projects. These libraries provide support in a number of domains (input-output, data structures, math, templates, ...). All other third party software has been included in source form and is injected in the build process at source level.

MAC OSX 10.9	CMake 3.3	GCC 5.2	Boost 1.59	Qt 5.4	HDF5 1.8	swig 3.0
MAC OSX 10.9	CMake 3.3	Clang 3.9	Boost 1.59	Qt 5.4	HDF5 1.8	swig 3.0
MAC OSX 10.11	CMake 3.4	GCC 5.3	Boost 1.59	Qt 5.5	HDF5 1.8	swig 3.0
MAC OSX 10.11	CMake 3.4	Clang 3.9	Boost 1.59	Qt 5.5	HDF5 1.8	swig 3.0
MAC OSX 10.11	CMake 3.5	GCC 6.1	Boost 1.59	Qt 5.5	HDF5 1.10	swig 3.0
MAC OSX 10.11	CMake 3.5	Clang 3.9	Boost 1.59	Qt 5.5	HDF5 1.10	swig 3.0
MAC OSX 10.11	CMake 3.5	AppleClang 7.3	Boost 1.59	Qt 5.5	HDF5 1.10	swig 3.0
Ubuntu 12.04	CMake 2.8	GCC 4.8	Boost 1.54	Qt 4.8	HDF5 1.8	swig 2.0
Ubuntu 12.04	CMake 2.8	GCC 5.1	Boost 1.53	Qt 4.8	HDF5 1.8	swig 2.0
Ubuntu 14.04	CMake 2.8	GCC 5.1	Boost 1.55	Qt 5.2	HDF5 1.8	swig 2.0
Ubuntu 14.04	CMake 2.8	Clang 3.6	Boost 1.55	Qt 5.2	HDF5 1.8	swig 2.0
Ubuntu 14.04	CMake 2.8	GCC 5.1	Boost 1.54	Qt 5.2	HDF5 1.8	swig 2.0
Ubuntu 16.04	CMake 3.5	GCC 5.3	Boost 1.58	Qt 5.5	HDF5 1.8	swig 3.0
Win7/MinGW 4.8	CMake 2.8	GCC 4.8	Boost 1.53	Qt 4.8	HDF5 1.8	swig 2.0
Win7/MinGW 4.9	CMake 2.8	GCC 4.9	Boost 1.53	Qt 5.6	HDF5 1.8	swig 2.0

Figure 6.2: Full list of build-and-test platforms. Situation of late June 2016.

The build-and-test process takes from a few minutes to up to an hour depending on the hardware platform. It benefits significantly from the parallel Make features if executed on a multi-core platform.

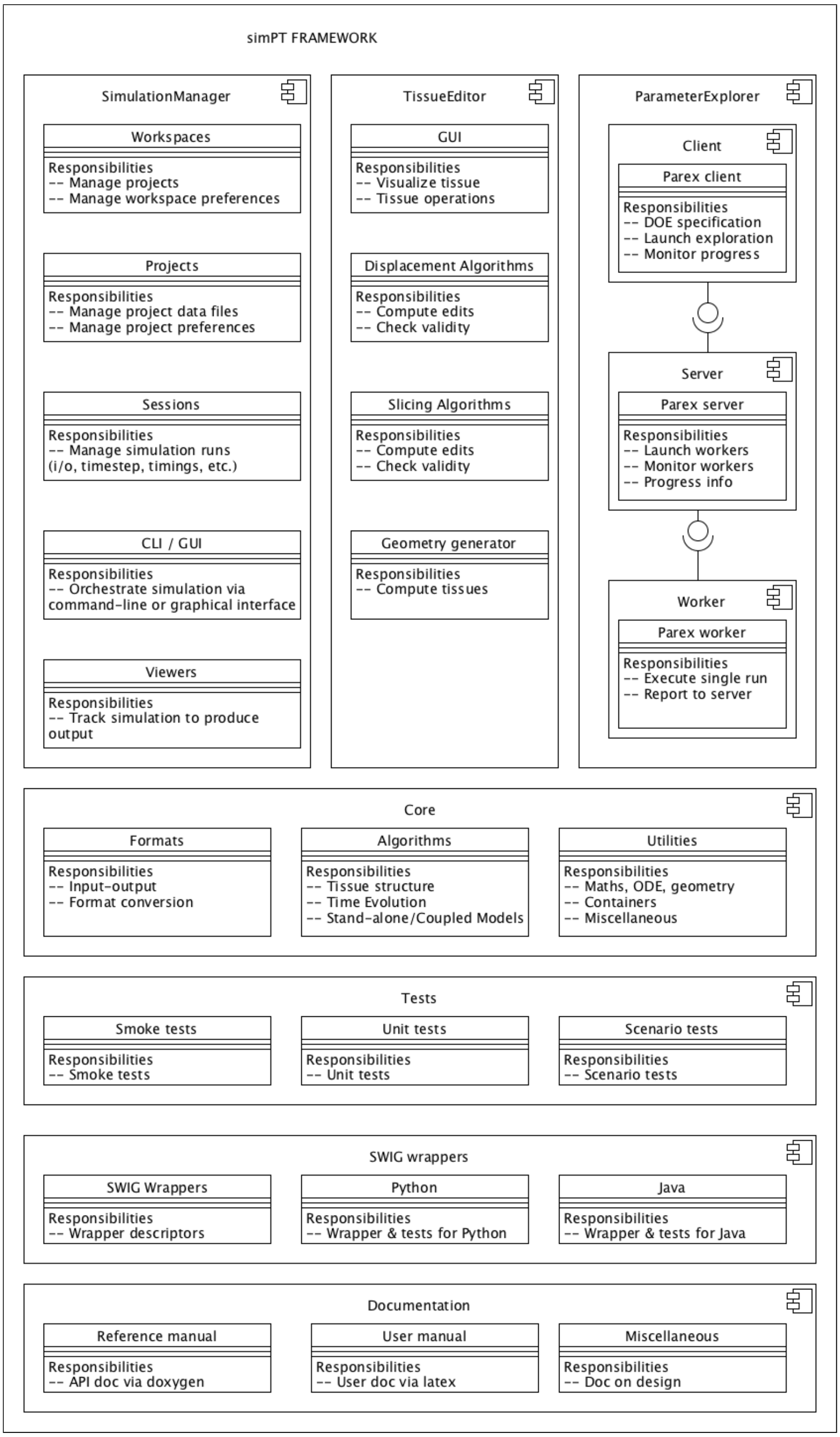

Figure 6.3: VPTissue framework.

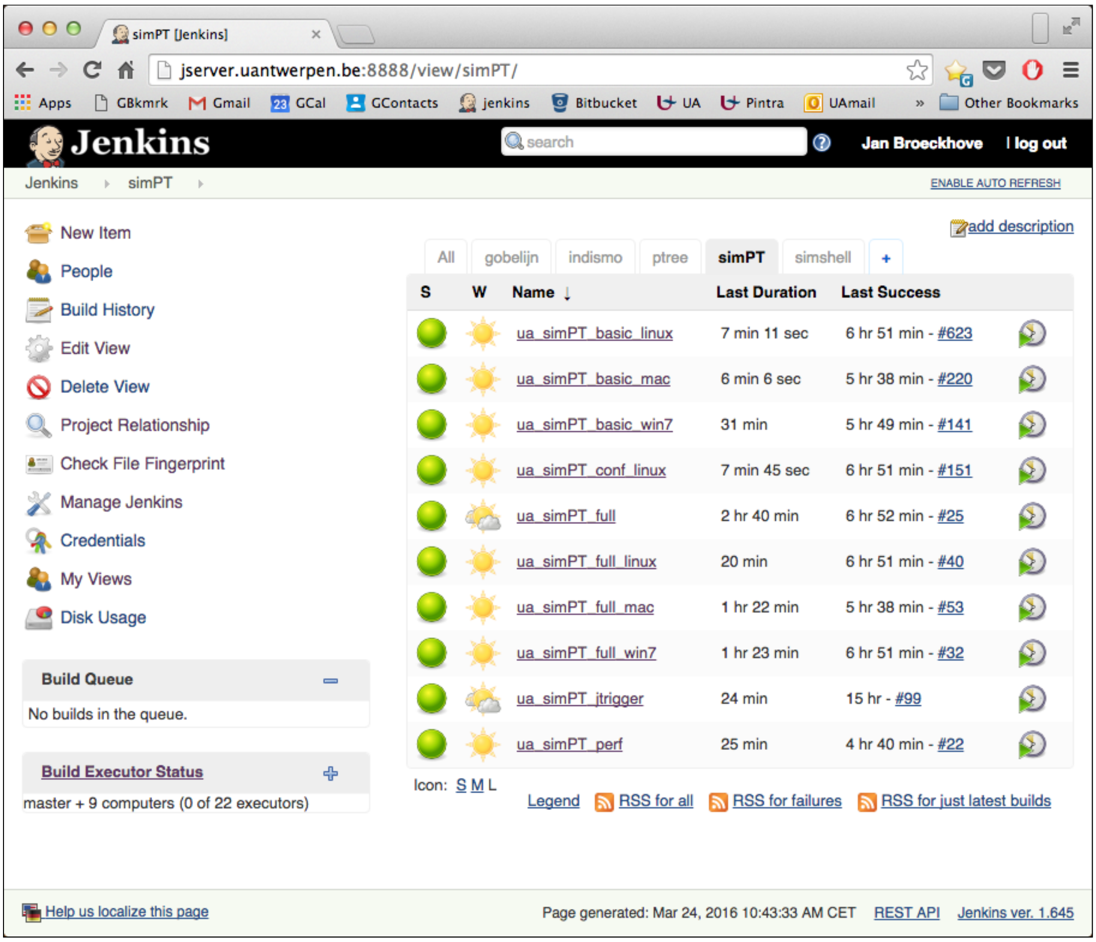

Figure 6.4: Screen shot of the overview page of the Jenkins server showing (in this instance) perfect status for all jenkins jobs.

CHAPTER 7

Programming with the VPTissue framework

VPTissue is a simulation framework with C++ as its development language. The code is up-to-date with the C++11 standard ([5, 12] and adheres to current coding practices.

We have done our utmost to facilitate the job of extending or modifying the simulator and or the models it simulates through separation into layers and packages, minimization dependencies, classes with clear cut responsibilities, naming etcetera.

Developers adopting the platform will of course need to spend some time inspecting the code and familiarizing themselves with it. The first task will most probably be adding models which means adding components and or attributes to the code base. in the section below we describe briefly how to go about these tasks.

7.1 Adding models and components

The code to deal with models is to be found in two locations:

src/main/cpp_sim/model
: This code specifies the model interfaces and the factory construction provides a component, given its name. The code need only be extended if and when the user decides to introduce a new component type. In that case a copy-paste-modify approach starting from an existing component is the easy way to make the new code.

src/main/models/"model family name"/components/"component type"
: This code contains the components for each of the model families, separated into a sub directory per component type. If and when the user intends to add a specific component of an existing model family, the code for that component

has to be put into the sub directory of the corresponding type and the file `factories.cpp` in the sub directory has to be edited to register the new component and its name and the `CMakeLists.txt` file has to be edited to include the new component. Again, the copy-paste-modify approach is the easiest. To add a new model all specific components need to be added.

If and when the user wants to introduce a new model family, he has to remember that it is possible to "borrow" components for the Default model family, simply by not providing your own implementation – see section 7.1. Thus a new sub directory `src/main/models/''model family name''/components/''component type''` has to be created only for each of the component types for which one has a specific implementation. The file implementing the component(s) and files `factories.h` and `factories.cpp` need to be added to the subdirectory. The code for the component and for the factories is again straightforward to make with the copy-paste-modify approach. Finally the `CMakeLists.txt` file has to be copied and edited to reflect the names of the new component files.

In addition to generating the code for new models one needs to provide input data file. The can be built from scratch using the VPTissue Tissue editor (see 4) or by copy-paste-modify of an existing input file. The modification can be done either with a text editor, an xml editor or the VPTissue Tissue editor, depending on your preference and the number of changes that need to be made.

To implement a so called meta-component that combines the code of different (sub-)components, a new component class (*e.g.* MetaSplit) has to be created and the .h files of the sub-components need to be included in the header. Both the Initialize and operator functions need to be adapted similar to the example below.

```
#include MetaSplit.h
#include Split1.h
#include Split2.h

void MetaSplit::Initialize(const CoreData& cd)
{
    m_cd =cd;
    ...
    m_splitter1 = (g_component_factories.at("Split1"))(m_cd);
    m_splitter2 = (g_component_factories.at("Split2"))(m_cd);
}

std::tuple<bool, bool, std::array<double>, 3>> MetaSplit::operator()\
(Cell* cell)
{
    ...
    return ('some condition') ? m_splitter1(cell): m_splitter2(cell);
}
```

7.2 Adding an attribute

When you want to add a new model or simulation parameter (like a kinetic constant) you simply need to add the text field with its value to the data input file ('leaf file'). Its value is then accessible to the component classes via the CoreData data structure, like other parameters. When you want to add an attribute to the tissue, cell, wall or node, you need to take a number of steps. We detail them here, by way of example, for the addition of an attribute name `solute` to cells. The cases for wall and node are similar

- Write the attribute into the `CellAttributes.h` header file and add getter and setter methods for the attribute if needed.

```
public:
    double GetSolute() const {return m_solute;}
    void   SetSolute(double s) {m_solute = s;}
protected:
    double m_solute;
```

- In the `CellAttributes.cpp` implementation file initialize the new attribute in the constructors and assignment operator.

```
CellAttributes::CellAttributes(...)
    , m_solute (0.0);

CellAttributes&CellAttributes::operator=(...)
m_solute = src.m_solute;
```

- In the `CellAttributes.cpp` implementation file extend the `ReadPtree` and `ToPtree` methods to include processing of the new attribute.

```
void CellAttributes::ReadPtree (...)
m_solute = cell_pt.get<double>("solute");

ptree CellAttributes::ToPtree() const
ret.put("solute", m_solute);
```

- In the `MBMBuilder.cpp` implementation file extend the `BuildCells` method to include processing of the new attribute.

```
void MBMBDirector::BuildCells(...)
attributes_pt.put("solute",
    mesh_state.GetCellAttribute<double>(cell_idx, "solute"));
```

- In the `Mesh.cpp` implementation file extend the `GetState` method to include processing of the new attribute.

```
MeshState Mesh::GetState() const
// Define the attribute
state.AddCellAttribute<double>("solute");
// Set cell attribute value in state
state.SetCellAttribute<double>(cell_idx, "solute", c->GetSolute());
```

- Add the new attribute to the input xml files. Do this for the template workspace xml files and for the files in the test directories.
- Edit the tooltips code if you want the new attribute to show up in the tooltip.

APPENDIX A

Parameter Dictionary

An overview of all parameters used in the simulator, specifying the defaults (if any), the choices that are currently available and a (very) brief indication of the role the parameter plays.

The parameter are subdivided in sections. These correspond to sub-nodes in the xml tree representation of the parameter set with the same name. Most of the parameters in the model section (cell_chemical_count and time_step being the exceptions) refer to model components.

A.1 Parameters in model

group
: The name of the model group that this model belongs to. *choices:* Default, plus any groups you yourself have developed.

name
: The name of the model to be simulated.
choices: AuxinGrowth, Geometric, Meinhardt, SmithPyllotaxis, NoGrowth, TipGrowth, TestCoupling, TestCoupling_I, TestCoupling_II, Wortel, WrapperModel

cell_chemical_count
: The number of chemicals in each cell.
choices: any positive integer number

cell_chemistry
: The method defining the dynamics of chemicals inside cells (e.g. auxin, cytokinin, ...).
choices: AuxinGrowth, Meinhardt, NoOp, PINFlux, PINFlux2, SmithPyllotaxis, Source, TestCoupling, Wortel, WrapperModel

cell_daughters
The method defining the partitioning of chemicals between parent and daughter cells upon division.
choices: Auxin, BasicPIN, NoOp, Perimeter, PIN, SmithPyllotaxis, Wortel, WrapperModel

cell_housekeep
The method for updating parameters related to cell and cell wall growth.
choices: Auxin, AuxinGrowth, BasicAuxin, Geometric, Meinhardt, NoOp, SmithPyllotaxis, Wortel, WrapperModel

cell_split
The method defining the cell division rules (e.g. cell divides, when its area is two times larger than the base area of the cell).
choices: AreaThresholdBased, AuxinGrowth, Geometric, NoOp, Wortel, WrapperModel

cell2cell_transport
The method defining transport dynamics (influx/efflux) across from cell to cell across cell walls.
choices: AuxinGrowth, Basic, Meinhardt, NoOp, Plain, SmithPyllotaxis, Source, TestCoupling, Wortel, WrapperModel

cell2cell_transport_boundary
Boundary conditions for cell to cell transport in coupled systems.
choices: TestCoupling_I, TestCoupling_II

mc_delta_hamiltonian
The DeltaHamiltonian used in the Metropolis method for the equilibration of cell and wall mechanics.
choices: ElasticWall, Maxwell, ModifiedGC, PlainGC

mc_hamiltonian
The Hamiltonian used in the Metropolis method for the equilibration of cell and wall mechanics.
choices: ElasticWall, Maxwell, ModifiedGC, PlainGC

mc_move_generator
Algorithm to generate random displacements for nodes in the Metropolis sweep.
choices: directed_normal, directed_uniform, standard_normal, standard_uniform

time_evolver
The name of the time evolution scheme to be used.
choices: Grow, Housekeep, HousekeepGrow, VPTissue, Transport, VLeaf

time_step
The time step used in the time evolution in the simulator.
choices: any real number

wall_chemistry
The method defining the dynamics of chemicals in cell walls (e.g. PIN transporters).
choices: AuxinGrowth, Basic, Meinhardt, NoOp

A.2 Parameters in auxin_transport

aux_breakdown
Rate constant of auxin breakdown.
choices: any real number

aux_cons
Auxin production rate outside shoot apical meristem.
choices: any real number

aux1prod
Rate of auxin production.
choices: any real number

D
Vector of diffusion coefficients.
choices: any real number

initval
Vector specifying standard starting values for cellular levels of chemicals after, for instance, division.
choices: any real number

k1
Rate constant for PIN translocation to the cell membrane (cfr. [1]).
choices: any real number

k2
Rate constant for return from cell membrane to endosome (cfr. [1]).
choices: any real number

ka
Saturation constant for PIN mediated transport of auxin (cfr. [1]).
choices: any real number

km
Saturation constant for exocytosis (cfr. [1].
choices: any real number

kr
Saturation constant for auxin dependence of PIN translocation (cfr. [1].
choices: any real number

leaf_tip_source
Auxin flux at the tissue boundary.
choices: any real number

pin_breakdown
Intracellular PIN breakdown rate constant.
choices: any real number

pin_production
Cellular PIN production rate.
choices: any real number

pin_production_in_epidermis

Cellular PIN production rate in epidermal cells (at boundary).
choices: any real number

r
Total level of surface auxin receptors.
choices: any real number

sam_auxin
Auxin level at the boundary with the apical meristem (sink).
choices: any real number

sam_auxin_breakdown
Rate constant for auxin breakdown in shoot apical meristem.
choices: any real number

sam_efflux
Maximum auxin efflux rate to shoot apical meristem.
choices: any real number

transport
Active (mediated) transport rate constant.
choices: any real number

A.3 Parameters in Blad0032, Blad0128, Blad0512 (leaf models)

These parameters are applicable only in the Blad (leaf) type models.

grid_size
Number of cells in the starting grid.
choices: integer: 32, 128, or 512.

M_degradation
Morphogen degradation rate constant.
choices: real number

M_diffusion_constant
Morphogen diffusion constant.
choices: real number

M_duration
Duration of morphogen production.
choices: real number

M_production
Morphogen production rate.
choices: real number

M_threshold_expansion
Minimum morphogen concentration for cell expansion.
choices: real number

M_threshold_division
Minimum morphogen concentration for cell division.
choices: real number

relative_growth
Rate of target area change (per housekeeping step).
choices: real number

size_threshold_division
Minimum cell area for division.
choices: real number

A.4 Parameters in cell_mechanics

auxin_dependent_growth
Dependence of growth on auxin.
choices: true, false

base_area
Base area of the cell used in the cell division condition.
default: 293.893
choices: any positive real number

cell_expansion_rate
Rate of target area change (per housekeeping step).
choices: any real number

collapse_node_threshold
Parameter defining the minimal distance between existing node and new node: if the new node is far enough (e.g. 5% of edge length) from one of the two existing nodes, the new node is inserted, else the existing node is used.
default: 0.05
choices: any real number

copy_wall
Copying wall elements to appropriate cells during cell division.
choices: true, false

division_ratio
Cell division factor: multiplier of base area.
default: 2.0
choices: any real number

elastic_modulus
Young elastic modulus in Elastic Wall and Maxwell models.
choices: any real number

lambda_alignment
Strength of cell alignment along given direction.
choices: any real number

lambda_bend
Strength of vertex bending in cell polygon.
choices: any real number

lambda_celllength
Strength of the cell length constraint.

choices: any real number

lambda_length

Spring constant of the edge (wall element): strength of the edge length constraint.

choices: any real number

mc_abs_tolerance

Absolute tolerance used in decision to continue Metropolis Monte Carlo sweeping: once below this tolerance we stop.

choices: any real number

mc_do_not_use_delta

Parameter for using the calculation of total energy (hamiltonian) or just energy difference (delta_hamiltonian).

choices: false, true

mc_rel_tolerance

Relative tolerance used in Metropolis Monte Carlo sweeping: once below tolerance (relative to total mesh energy) we stop.

choices: any real number

mc_retry_limit

How many times we do additional Metropolis Monte Carlo sweeps when the current sweep increases energy instead of lowering it.

choices: unsigned integer

mc_sliding_window

Metropolis Monte Carlo sweeping averages energy changes over a number of mc_sliding_window sweeps to decide to stop or continue.

choices: unsigned integer

mc_stepsize

Maximum node displacement distance: multiplier of mc_move_generator(). It is used in the displacements of the nodes in the Metropolis Monte Carlo algorithm for finding the equilibrated state.

choices: any real number

mc_step_x_scale

For move generator of directed type, this number scales steps in the x direction.

choices: any real number

mc_step_y_scale

For move generator of directed type, this number scales steps in the y direction.

choices: any real number

mc_sweep_limit

Maximum number of sweeps that can be executed in a single time step: to avoid infinite sweeps.

choices: unsigned integer

mc_temperature

Amount of noise; extent to which the system accepts energetically unfavourable moves.

choices: any positive real number

relative_perimeter_stiffness

Ratio between spring constants (lambda_length) of perimeter edges (wall elements) and internal edges (wall elements).
choices: any positive real number

response_time
Response time needed for turgor regulation used in Maxwell model.
default: 0.1
choices: any positive real number

target_node_distance
Rest length of edges (wall elements): constant and the same for all edges.
default: 3.09017
choices: any positive real number

viscosity_const
Viscosity used in Maxwell model.
choices: any positive real number

yielding_threshold
Parameter used in edge (wall element) yielding threshold: multiplier of target_node_distance.
choices: any positive real number

A.5 Parameters in ode_integration

abs_tolerance
Absolute tolerance used in the ODE solver algorithms.
choices: any positive real number

ode_solver
ODE solver algorithm to be used for reaction, diffusion and active transport equations.
default: runge_kutta_dopri5
choices: adams_bashforth, bulirsch_stoer, euler, modified_midpoint, runge_kutta4, runge_kutta_cash_karp54, runge_kutta_dopri5, runge_kutta_fehlberg78

rel_tolerance
Relative tolerance used in de ODE solver algorithms.
choices: any positive real number

small_time_increment
Time increment used in ode_solver.
choices: any positive real number

A.6 Parameters in random_engine

type
The random number engines available for use (see the C++11

Standard Library documentation).
default: mt19937_64 *choices:* minstd_rand0, minstd_rand, mt19937, mt19937_64, ranlux24_base, ranlux48_base, knuth_b

seed
The seed for the random engine (the default makes the simulation results non-reproducible).
default: time reading generates random seed
choices: unsigned integer

A.7 Parameters in smith_phyllotaxis

These parameters are applicable only in the SmithPhyllotaxis model.

k_IAA
IAA production limiting coefficient (saturation).
choices: any real number

k_PIN
PIN1 production limiting coefficient (saturation).
choices: any real number

k_T
Coefficient controlling the saturation of IAA transport.
choices: any real number

mu_IAA
IAA decay.
choices: any real number

mu_PIN
PIN1 decay.
choices: any real number

rho_IAA
IAA production.
choices: any real number

rho_PIN0
Auxin independent PIN1 production.
choices: any real number

rho_PIN
PIN1 production.
choices: any real number

c
Controls PIN1 distribution.
choices: any real number

D
IAA diffusion coefficient.
choices: any real number

T

IAA active transport coefficient.
choices: any real number

A.8 Parameters in termination

max_cell_count

Maximal number of cells in the mesh to be reached for the pause (in GUI mode) or termination (in CLI mode) of the simulator.
default: no limit
choices: unsigned integer

max_sim_time

Maximal number of time steps to be reached for the pause (in GUI mode) or termination (in CLI mode) of the simulator.
default: no limit
choices: unsigned integer

stationarity_check

Stationarity of ODE solution within abs_tolerance to be reached for the termination of the simulator.
choices: true, false

A.9 Parameters in TestCoupling, TestCoupling_I, TestCoupling_II

These parameters are applicable only in the TestCoupling type models.

ch0_breakdown

Chemical 0 degradation rate constant.
choices: real number

ch0_production

Chemical 0 production rate.
choices: real number

ch1_breakdown

Chemical 1 degradation rate constant.
choices: real number

ch1_production

Chemical 1 production rate.
choices: real number

D

Vector of transport coefficients for individual models.
choices: any real number

diffusion
Transport coefficient for coupled cells in coupled simulation (one value per cell couple).
choices: any real number

sim_ODE_coupling_steps
Number of coupling steps per simulation time step.
choices: real number

A.10 Parameters in Wortel (root)

These parameters are applicable only in the Wortel (root) model.

apoplast_thickness
Assumed thickness of the apoplast compartment separating two cells.
choices: real number

aux_breakdown
Cellular auxin breakdown rate constant.
choices: real number

aux_production
Cellular auxin production rate constant.
choices: real number

aux_shy2_breakdown
Auxin-dependent Shy2 breakdown rate constant.
choices: real number

aux_sink
Rate constant for outgoing auxin flux.
choices: real number

aux_source
Rate constant for incoming auxin flux.
choices: real number

ck_breakdown
Cellular cytokinin breakdown rate constant.
choices: real number

ck_shy2_production
Cytokinin dependent production rate constant.
choices: real number

ck_sink
Rate constant for outgoing cytokinin flux.
choices: real number

ck_source
Rate constant for incoming cytokinin flux.
choices: real number

D

Vector of diffusion coefficients (index '0' for auxin; index '1' for cytokinin).
choices: any real number

ga_breakdown
Cellular gibberellin breakdown rate constant.
choices: any real number

ga_production
Cellular gibberellin production rate constant.
choices: any real number

ga_threshold
Minimum gibberellin concentration for cell division and growth.
choices: any real number

k_export
Auxin export permeability.
choices: any real number

k_import
Auxin import permeability.
choices: any real number

km_aux_ck
Auxin-dependent cytokinin inhibition constant.
choices: any real number

km_aux_shy2
$S_{0.5}$ for auxin-dependent Shy2 breakdown .
choices: any real number

km_shy
Shy2-dependent auxin export inhibition constant.
choices: any real number

shy2_breakdown
Cellular Shy2 breakdown rate constant.
choices: any real number

shy2_production
Cellular Shy2 production rate constant.
choices: any real number

shy2_threshold
Maximum Shy2 concentration for cell division.
choices: any real number

vm_aux_ck
Auxin-dependent cytokinin production rate constant.
choices: any real number

A.11 Parameters in WrapperModel

These parameters are applicable only in the WrapperModel model.

cell_division_threshold
Minimum cell area for division.
choices: real number

ch0_breakdown
Chemical 0 degradation rate constant.
choices: real number

ch0_production
Chemical 0 production rate constant.
choices: real number

ch0_threshold
Minimal chemical 0 concentration for cell expansion.
choices: real number

ch1_breakdown
Chemical 1 degradation rate constant.
choices: real number

ch1_production
Chemical 1 production rate constant.
choices: real number

D
Vector of transport coefficients.
choices: any real number

expansion_rate
Rate of target area change (per housekeeping step).
choices: real number

APPENDIX B

Preferences Dictionary

B.1 Introduction

In this section we give an overview of all preferences that apply to VPTissue as a command line or graphical application. At the present level of implementation, all preferences deal with viewers: graphical viewers, output viewers and log viewers.

Preference definitions come in two distinct flavors, each in a separate file namely `.simPT-cli-preferences.xml` and `.simPT-gui-preferences.xml`. The first, with keyword `cli` (i.e. Command-Line Interface) in the name, applies to the command line application, the second, with the keyword `gui` (i.e. Graphical User Interface) in the name, applies to the graphical application.

The workspace preferences act as defaults for those project preferences that do not have an actual value, but instead have a `$WORKSPACE$` label as a value. An actual value for a project preference overrides the workspace default for that preference.

The installed application defines a template for the `cli` and `gui` preferences that are used when creating a new workspace or when opening a workspace where the preferences are missing. When creating a new project or opening a project where the preferences are missing, the workspace preferences are copied to define the initial project preferences.

B.2 Preferences for graphics: colors_sizes

Options for the color and size of text of mesh objects.

arrow_color
choices: any color name acceptable to Qt4
arrow_size
choices: unsigned integer
background_color
default: white
choices: any color name acceptable to Qt4
cell_color
Cell corloring scheme to be used when generating images of the tissue.
choices: AuxinPIN1, ChemBlue, ChemGreen, Meinhardt, Size, Wortel
cell_number_size
choices: unsigned integer
cell_outline_color
choices: any color name acceptable to Qt4
node_magnification
choices: unsigned integer
node_number_size
choices: unsigned integer
outline_width
choices: unsigned integer
resize_stride
choices: unsigned integer
text_color
choices: any color name acceptable to Qt4

B.3 Preferences for graphics: visualization

Options for the view of mesh objects.

border_cells
choices: false, true
cells
choices: false, true
cell_axes
choices: false, true
cell_centers
choices: false, true
cell_numbers
choices: false, true

cell_strain
choices: false, true (currently no model implementation)
fluxes
choices: false, true
nodes
choices: false, true
node_numbers
choices: false, true
only_leaf_boundary
choices: false, true
tooltips
View of cell data (number, type, chemicals, area, etc.) by mouse pointer.
choices: false, true
walls
choices: false, true

B.4 Preferences for file format: bitmap_graphics

Options for output of bitmap-files. These options are used for BMP, JPEG and PNG export or post-processing.
size
Size of generated bitmaps. This node is optional.
x
Width of generated bitmaps.
default: 800
choices: unsigned integer
y
Height of generated bitmaps.
default: 600
choices: unsigned integer
source_window
Rectangular part of mesh to export. Coordinates in mesh-domain.
This node is optional. If it isn't specified, the source window will be the bounding box of the mesh.
min_x
X-coordinate of top-left corner of source window.
default: -
choices: any real number
min_y
Y-coordinate of top-left corner of source window.
default: -
choices: any real number
max_x

X-coordinate of bottom-right corner of source window.
default: -
choices: any real number

max_y
Y-coordinate of bottom-right corner of source window.
default: -
choices: any real number

B.5 Preferences for viewer: hdf5

Option for output of hdf5-files.

enabled_at_startup
The viewer is disabled (inactive) or enabled (active).
default: -
choices: false, true

stride
The viewer skips time steps and takes action at steps corresponding to multiples of the stride.
default: -
choices: unsigned integer

file
The name of the hdf5 output file containing the history of the simulation for that project. Note that each project lives in its own subdirectory, so it is perfectly fine to have the same filename, e.g., `leaf.h5` for all projects.
default: -
choices: any allowed filename with .h5 extension

B.6 Preferences for viewer: log

enabled_at_startup
The viewer is disabled (inactive) or enabled (active).
default: -
choices: false, true

B.7 Preferences for viewer: logwindow

enabled_at_startup
The viewer is disabled (inactive) or enabled (active).
default: -
choices: false, true

position

x
Position of logwindow in x-axis.
choices: unsigned integer

y
Position of logwindow in y-axis.
choices: unsigned integer

size

x
Size of logwindow in x-axis.
choices: unsigned integer

y
Size of logwindow in y-axis.
choices: unsigned integer

B.8 Preferences for viewer: qt

enabled_at_startup
The viewer is disabled (inactive) or enabled (active).
default: -
choices: false, true

stride
The viewer skips time steps and takes action at steps corresponding to multiples of the stride.
default: -
choices: unsigned integer

position

x
Position of logwindow in x-axis.
choices: unsigned integer

y
Position of logwindow in y-axis.
choices: unsigned integer

size

x
Size of logwindow in x-axis.
choices: unsigned integer

y
Size of logwindow in y-axis.
choices: unsigned integer

B.9 Preferences for file format: vector_graphics

Options for output of vector graphics-files. These options are used for PDF export or post-processing.

source_window

Rectangular part of mesh to export. Coordinates in mesh-domain.
This node is optional. If it isn't specified, the source window will be the bounding box of the mesh.

min_x
X-coordinate of top-left corner of source window.
default: -
choices: any real number

min_y
Y-coordinate of top-left corner of source window.
default: -
choices: any real number

max_x
X-coordinate of bottom-right corner of source window.
default: -
choices: any real number

max_y
Y-coordinate of bottom-right corner of source window.
default: -
choices: any real number

B.10 Preferences for viewer: xml

Option for output of xml-files.

enabled_at_startup

The viewer is disabled (inactive) or enabled (active).
default: -
choices: false, true

stride

The viewer skips time steps and takes action at steps corresponding to multiples of the stride.
default: -
choices: unsigned integer

gzip

The viewer is disabled (inactive) or enabled (active) for packing the xml-files.
default: -
choices: false, true

APPENDIX C

VPTissue HDF5 file specification

The VPTissue framework uses a consistently defined file format for data storage and interchange that is based on the HDF5 storage format for numerical data. Hierarchical Data Format 5 files are self-descriptive and well structured. This document describes the structure of HDF5 based storage for data files used in VPTissue and its ecosystem of related tools.

The internal data structure inside VPTissue can be summarized by the following statements:

- nodes are points in space
- cells are polygons with nodes connected by edges nodes
- walls (membranes) are defined as entities separating two cells
- cells know their nodes and walls (and hence their neighbouring cells)
- walls know the two cells they connect and their two end-nodes

This is a (simplified) variant of what's commonly called a *winged edge*[1] representation. We use these connections to define arrays in the HDF5 file that describe the tissue structure.

HDF5 is a standardized file format for storing general numerical data in a hierarchical structure. The two main types of entities in a HDF5 file are *groups* and *datasets*.

- Groups define the hierarchical structure of the data. They are analogous to directories or file folders in a (UNIX-like) file system.

[1] http://en.wikipedia.org/wiki/Winged_edge

- Datasets contain numerical data stored in general rank-n arrays of well-defined dimensions and type of data they store. They are the "files" in the file system analogy.

Navigating through a HDF5 file is also very analogous to browsing through a file system: The root of the hierarchy is denoted by `/` and subgroups and datasets are addressed by separating their names with a `/`. For example: a dataset called `nodes_xy` inside a subgroup `step_2` of the root group `/` has the "absolute" path `/step_2/nodes_xy`.

A HDF5 file is self-descriptive in the sense that it contains all the necessary information to read the stored information correctly without any other additional resources. For instance, datasets know the numeric type of data they contain. The format, however, does not define any semantics or interpretation of the data. A gentle overview of HDF5 can be found on Wikipedia[2]. More in-depth info and technical documentation on HDF5 can be found on the HDF Group website[3].

C.1 HDF5 file stucture

C.1.1 Root level

The root of the VPTissue file contains two datasets related to the time progression of the simulation. They are required and must be extendable, which means they can grow or shrink as more simulation steps are appended or unnecessary steps are being purged.

Name	Data type	Dimensions	Notes
`time_steps`	`double`	(#steps)	Time stamps in simulation specific time units
`time_steps_idx`	`int`	(#steps)	Index values of the simulation steps

Table C.1: Time steps

The important point to note is that the file contains a subset of all the steps performed by the simulator, depending on the write-stride. For example: if the stride is 3 and the simulator ran 10 steps starting from 0 we would end up having:

- `time_steps` : `[0.0, 0.3, 0.6, 0.9]`
- `time_steps_idx` : `[0, 3, 6, 9]`

The numbers in `time_steps_idx` translate which m-th saved state corresponds to which n-th simulator state. The state of the simulation at specific times is saved in separate `/step_{n}` subgroups of the `/` group. Note that in the case of stride being 1, n will have the same meaning as m. Although this is not true in a general case.

[2] https://en.wikipedia.org/wiki/Hierarchical_Data_Format

[3] https://www.hdfgroup.org/

C.1.2 Inside a /step_{n} group

For a given time step index n the `/step_{n}` group contains all the information about the geometry of the tissue, the values of all the attributes that define the geometry as well as the parameters used in the current simulation step. Table C.2 summarises all the datasets found inside a `/step_{n}` that are relevant to geometry and connectivity of cells:

Name	Data type	Dimensions	Notes
`nodes_id`	`int`	(#nodes)	IDs of all the nodes in the tissue
`nodes_xy`	`double`	(#nodes,2)	2D coordinates of nodes. In figure C.1 nodes are represented by yellow points labeled with black numbers.
`cells_id`	`int`	(#cells)	IDs of all the cells in the tissue
`cells_num_nodes`	`int`	(#cells)	Number of nodes (yellow points in C.1) used to define the polygon of each cell.
`cells_nodes`	`int`	(#cells,max(#nodes/cell))	Each row represents a cell. The elements in each row are indices of the nodes (as defined in `nodes_xy`) that define the polygon of the cell in counterclockwise order. As such, this dataset defines the geometry of all cells. For conveniece, rows that represent cells with less than maximal number of nodes are padded with -1 (which is an invalid node index).
`cells_num_walls`	`int`	(#cells)	Number of walls for each cell.
`cells_walls`	`int`	(#cells,max(#walls/cell))	IDs of walls in each cell.
`walls_id`	`int`	(#walls)	IDs of all the walls in the tissue
`walls_cells`	`int`	(#walls,2)	Indices of the two cells connected by a wall. The region outside the tissue is labeled by -1. All "outer" cells that lie on the boundary of the tissue have this special -1 cell among their neighbors. In figure C.1 walls are denoted by red labels.
`walls_nodes`	`int`	(#walls,2)	Each wall has two extreme nodes. The indices of these nodes are kept in this dataset. The order in which the nodes are specified is tied to the order in which the two neighboring cells are given in `walls_cells`. See red arrows in C.1 for a visual clarification of the orientation that is used.

Table C.2: Required datasets inside one step

Additionally, nodes, cells and walls can have an arbitrary number of named attributes with values of any type. These are defined by datasets with a common prefix name such as `nodes_attr_` concatenated with the name of the attribute.

Name	Data type	Dimensions	Notes
`nodes_attr_{name}`	*any*	(#nodes)	Datasets with values of node-based attributes
`cells_attr_{name}`	*any*	(#cells)	Datasets with values of cell-based attributes
`walls_attr_{name}`	*any*	(#walls)	Datasets with values of wall-based attributes

Table C.3: Optional datasets inside one step related to cell/node/wall attributes

The used data types are summarized in tabel C.4.

Name	HDF5 data type	Notes
`int`	`H5T_STD_I32LE`	or compatible, such as `H5T_NATIVE_INT`
`double`	`H5T_IEEE_F64LE`	or compatible, such as `H5T_NATIVE_DOUBLE`
`string`	`H5T_STRING`	Variable length, C-style null-terminated string

Table C.4: HDF5 data types

C.2 Tools

C.2.1 HDFView

HDF5 files contain data in binary form and are not human readable. HDFView[4] is a Java-based easy to use graphical tool to browse, create and modify HDF5 file. You can use it to quickly inspect VPTissue output for further processing.

C.2.2 Python

Python supports handling of HDF5 files through the `h5py`[5] package. Most probably you can install it using your system's package manager or with `pip`. Access to the data in HDF5 files is provided using `numpy`[6] arrays which makes working with `h5py` very straightforward.

The following Python script can be used to plot the example file used above (or any other file from VPTissue, since this script only uses a rather small subset of the available data).

[4] http://www.hdfgroup.org/hdf-java-html/hdfview/
[5] http://www.h5py.org/
[6] http://www.numpy.org/

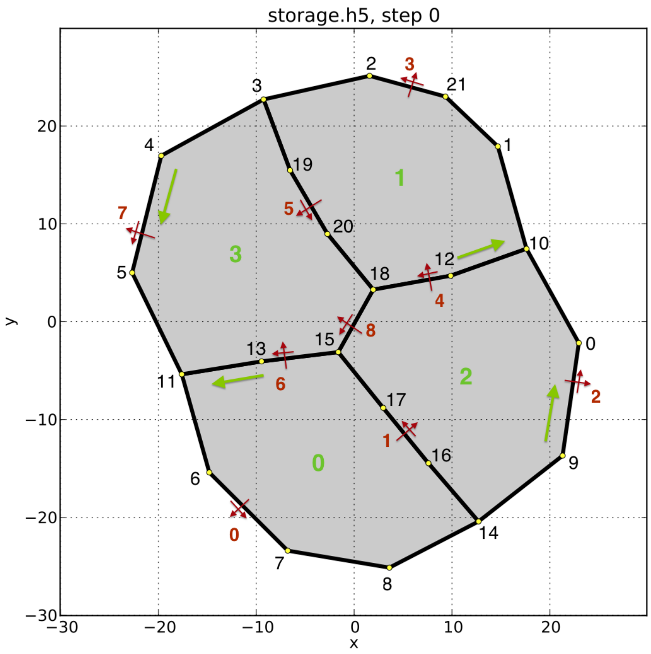

Figure C.1: A schematic representation of the geometry as used by VPTissue. Yellow points are the *nodes*, labeled by black numbers. Green numbers represent the labels of the four *cells* in this particular example. The green arrows show how nodes are connected to form a cell. For example: cell 1 is made up from nodes (12, 10, 1, 21, 2, 3, 19, 20, 18), in that precise order. Red labels represent the walls. For example: wall 5 connects cells 1 and 3, in that precise order.

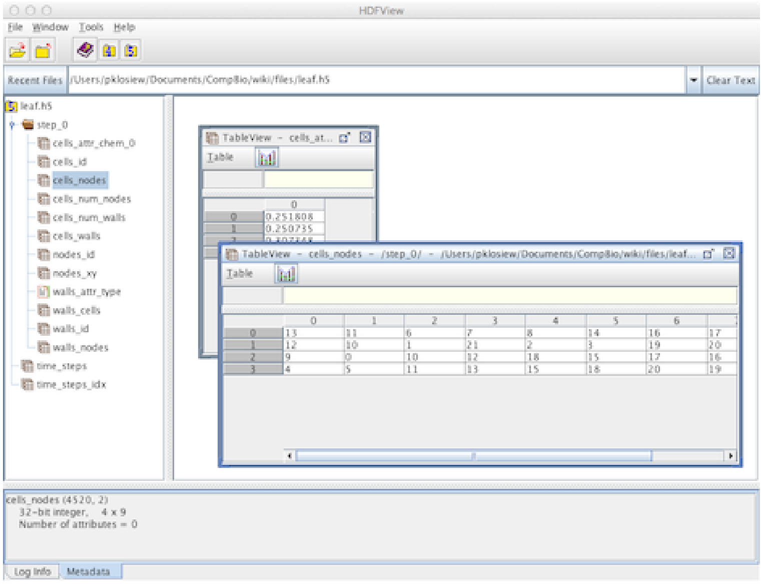

Figure C.2: A screenshot of the HDFView program while browsing the contents of a VPTissue results file.

```
import h5py
import argparse
import numpy as np
import matplotlib.cm as cm
import matplotlib.pyplot as plt
from matplotlib.patches import Polygon
from matplotlib.collections import PatchCollection

#==============================================================================
# Check for command line options
parser = argparse.ArgumentParser(description='Plot simPT HDF5 results')
parser.add_argument(
    'filename',
    action='store',
    help='simPT HDF5 file'
)
parser.add_argument(
    '--step', '-s',
    action='store',
    dest='step_idx',
    help='Index of simulation step to load'
)
args = parser.parse_args()

#==============================================================================
# Open & read the HDF5 file in read-only mode
tissue_file = h5py.File(args.filename, 'r')

if args.step_idx != None:
    step_idx = args.step_idx
```

```
else:
    # Take the last step if none was given
    step_idx = tissue_file['time_steps_idx'][-1]

# Read a subset of the tissue data from a particular step
# (after checking it actually exists)
step_grp_name = ('/step_{0}').format(step_idx)
if step_grp_name not in tissue_file:
    print ('Step with index {0} not found!').format(step_idx)
    exit(1)
else:
    step_grp = tissue_file[step_grp_name]

nodes_id = step_grp['nodes_id'][...]
nodes_xy = step_grp['nodes_xy'][...]

cells_id = step_grp['cells_id'][...]
cells_num_nodes = step_grp['cells_num_nodes'][...]
cells_nodes = step_grp['cells_nodes'][...]
cells_chem = step_grp['cells_attr_chem_0'][...]

# Close HDF5 file
tissue_file.close()

#==============================================================================
# Some minor processing of the data
num_nodes = nodes_id.shape[0]
num_cells = cells_id.shape[0]

print ('Number of nodes: {0}').format(num_nodes)
print ('Number of cells: {0}').format(num_cells)

# Dictionary that translates from node IDs to node index values
nodes_idx = {nodes_id[node_idx]: node_idx for node_idx in xrange(num_nodes)}

#==============================================================================
# Create the figure
fix,ax = plt.subplots()

# Create a list of polygons that represent the cells
polygons = []
for cell_idx in np.arange(num_cells):
    # IDs of the nodes that define the current cell ...
    cell_nodes_id = cells_nodes[cell_idx, :cells_num_nodes[cell_idx]]
    # ... are translated to node index values ...
    cell_nodes_idx = [nodes_idx[node_id] for node_id in cell_nodes_id]
    # ... and then used to extract appropriate node coordinates for this cell
    cell_nodes_xy = nodes_xy[cell_nodes_idx]

    # Create the cell's polygon and add it to the list of polygons to draw
    cell_poly = Polygon(cell_nodes_xy)
    polygons.append(cell_poly)

# Collection of patches to draw is created from the list of polygons
patch_coll = PatchCollection(polygons, cmap=cm.viridis)
```

```

# Set the array of per-polygon (cell) values used to color the cells
# (in this case, values of the first cell-based chemical saved as
# a cell-based attribute with the name 'chem_0')
patch_coll.set_array(cells_chem)

# Add the collection to draw to the axis
ax.add_collection(patch_coll)

# Create a legend-like color bar to show the range of cell based values
plt.colorbar(patch_coll)

# Set figure properties
# Adapt figure ranges to the tissue size
ax.set_xlim(np.min(nodes_xy[:, 0]) - 10, np.max(nodes_xy[:, 0]) + 10)
ax.set_ylim(np.min(nodes_xy[:, 1]) - 10, np.max(nodes_xy[:, 1]) + 10)
# Use a square grid
ax.set_aspect(1.)
ax.grid()
# Optional: In simPT the y-axis faces downwards, to make plots look
# similar to these from simPT you can just flip the y-axis with:
ax.invert_yaxis()

# Show the figure on screen
plt.show()
```

Running `python simPT_HDF5_example.py tissue.h5` gives figure C.3.

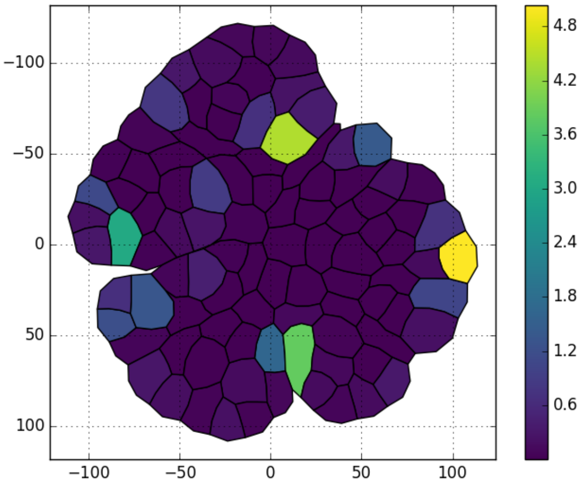

Figure C.3: A VPTissue HDF5 file plot with Python

C.2.3 Matlab

Matlab supports HDF5 files out of the box. See: MathWorks Documentation Center for a detailed overview[7]. Reading data from a VPTissue file (using the “High-Level” functions) is straightforward; a really basic example is shown in the following script.

```
leaf_file_name = 'your_simulation_result.h5';

time_steps = h5read(leaf_file_name, '/time_steps');
time_steps_idx = h5read(leaf_file_name, '/time_steps_idx');

num_cells = zeros(i,1);

for i = 1:length(time_steps)
    step_idx = time_steps_idx(i);
    cells_id = h5read(leaf_file_name, ['/step_', num2str(step_idx), '/cells_id']);
    num_cells(i) = length(cells_id);
end

plot(time_steps, num_cells, '.-');
xlabel('Time (sec)');
ylabel('Number of cells');
grid on;
```

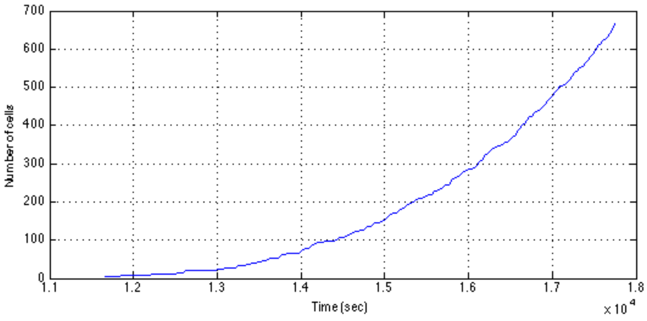

Figure C.4: Analysis of VPTissue HDF5 files in Matlab

C.2.4 ParaView

We have also developed a HDF5-based plugin for ParaView[8], which can be found in the `src/main/resources/paraview/` directory.

[7] http://www.mathworks.nl/help/matlab/hdf5-files.html

[8] http://www.paraview.org/

Bibliography

[1] R. M. H. Merks, Y. Van de Peer, D. Inzé, and G. T. S. Beemster, "Canalization without flux sensors: a traveling-wave hypothesis," *Trends in Plant Science*, vol. 12, pp. 384–390, 2007.

[2] R. M. H. Merks, M. Guravage, D. Inzé, and G. T. S. Beemster, "VirtualLeaf: an open-source framework for cell-based modeling of plant tissue growth and development.," *Plant physiology*, vol. 155, pp. 656–66, Mar. 2011.

[3] D. D. Vos, A. Dzurakhalov, D. Draelants, I. Bogaerts, S. Kalve, E. Prinsen, K. Vissenberg, W. Vanroose, J. Broeckhove, and G. T. S. Beemster, "Toward mechanistic models of plant organ growth," *Journal of Experimental Botany*, vol. 63, no. 9, pp. 3325–3337, 2012.

[4] J. J. Barton and L. R. Nackmann, *Scientific and Engineering C++*. Addison-Wesley Publishing Company, 1994.

[5] B. Stroustrup, *The C++ Programming Language, Fourth Edition C++11*. Pearson Education, Inc., Upper Saddle River, New Jersey, 2013.

[6] R. M. H. Merks and M. Guravage, *Building simulation models of developing plant organs in VirtualLeaf*, vol. 959 of *Methods in Molecular Biology*, ch. 23, pp. 333–352. Humana Press, Springer Protocols, 2013.

[7] H. Meinhardt, "Morphogenesis of lines and nets," *Differentiation*, vol. 6, pp. 117–123, 1976.

[8] R. Smith, S. Guyomarc'h, T. Mandel, D. Reinhardt, C. Kuhlemeier, and P. Prusinkiewicz, "A plausible model of phyllotaxis," *Proc Natl Acad Sci U S A*, vol. 103, pp. 1301–1306, 2006.

[9] D. Draelants, "Numerical analysis of pattern formation in auxin transport models," 2016. PhD thesis.

[10] D. De Vos, E. De Borger, J. Broeckhove, and G. Beemster, “Simulating leaf growth dynamics through metropolis-monte carlo based energy minimization,” *J Comput Sci*, vol. 9, pp. 107–111, 2015.

[11] D. De Vos, K. Vissenberg, J. Broeckhove, and G. Beemster, “Putting theory to the test: which regulatory mechanisms can drive realistic growth of a root?,” *PLoS Comput Biol*, vol. 10, p. e1003910, 2014.

[12] S. B. Lippman, J. Lajoie, and B. E. Moo, *C++ Primer, Fifth Edition C++11*. Addison Wesley, Inc., Upper Saddle River, New Jersey, 2013.

[13] E. Gamma, R. Helm, R. Johnson, and J. Vlissedes, *Design Patterns - Elements of Reusable Object Oriented Software*. Reading, Massachusetts: Addison-Wesley Publishing Company, Inc., 1995.

[14] B. Aiello and L. Sachs, *Configuration Management*. Addison Wesley, Inc., Upper Saddle River, New Jersey, 2011.

[15] J. Humble and D. Farley, *Continuous Delivery*. Addison Wesley, Upper Saddle River, New Jersey, 2011.
